# Design, Synthesis, and Biological Evaluations of Novel Thiazolo[4,5-d]pyrimidine Corticotropin Releasing Factor (CRF) Receptor Antagonists as Potential Treatments for Stress Related Disorders and Congenital Adrenal Hyperplasia (CAH)

**DOI:** 10.3390/molecules29153647

**Published:** 2024-08-01

**Authors:** Md Rabiul Islam, Christos Markatos, Ioannis Pirmettis, Minas Papadopoulos, Vlasios Karageorgos, George Liapakis, Hesham Fahmy

**Affiliations:** 1 Department of Pharmaceutical Science, College of Pharmacy & Allied Health Professions, South Dakota State University, Brookings, SD 57007, USA; mdrabiul.islam925@jacks.sdstate.edu; 2 Department of Pharmacology, School of Medicine, University of Crete, Heraklion, 71003 Crete, Greece; xristosmarkatos@gmail.com (C.M.); bkarageorgos@hotmail.com (V.K.); liapakig@uoc.gr (G.L.); 3 Institute of Nuclear & Radiological Sciences & Technology, Energy & Safety, National Centre for Scientific Research “Demokritos”, 15310 Athens, Greece; ipirme@rrp.demokritos.gr (I.P.); mspap@rrp.demokritos.gr (M.P.)

**Keywords:** thiazolo[4,5-d]pyrimidines, CRF receptor antagonists, congenital adrenal hyperplasia (CAH), antalarmin

## Abstract

Corticotropin-releasing factor (CRF) is a key neuropeptide hormone that is secreted from the hypothalamus. It is the master hormone of the HPA axis, which orchestrates the physiological and behavioral responses to stress. Many disorders, including anxiety, depression, addiction relapse, and others, are related to over-activation of this system. Thus, new molecules that may interfere with CRF receptor binding may be of value to treat neuropsychiatric stress-related disorders. Also, CRF_1_R antagonists have recently emerged as potential treatment options for congenital adrenal hyperplasia. Previously, several series of CRF_1_ receptor antagonists were developed by our group. In continuation of our efforts in this direction, herein we report the synthesis and biological evaluation of a new series of CRF_1_R antagonists. Representative compounds were evaluated for their binding affinities compared to antalarmin. Four compounds (**2**, **5**, **20**, and **21**) showed log IC_50_ values of −8.22, −7.95, −8.04, and −7.88, respectively, compared to −7.78 for antalarmin. This result indicates that these four compounds are superior to antalarmin by 2.5, 1.4, 1.7, and 1.25 times, respectively. It is worth mentioning that compound **2**, in terms of IC_50_, is among the best CRF_1_R antagonists ever developed in the last 40 years. The in silico physicochemical properties of the lead compounds showed good drug-like properties. Thus, further research in this direction may lead to better and safer CRF receptor antagonists that may have clinical applications, particularly for stress-related disorders and the treatment of congenital adrenal hyperplasia.

## 1. Introduction

**Figure 1:**
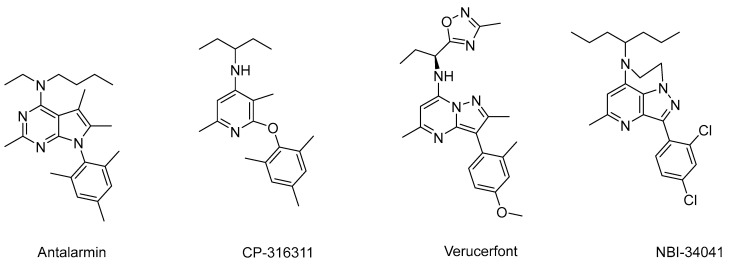
Important CRF_1_R antagonists.

Corticotropin-releasing factor (CRF) is a pivotal hormonal player in the physiology of stress. It was first discovered in the ovine hypothalamus in 1981 [1,2]. CRF is secreted from the hypothalamus region known as the paraventricular nucleus (PVN). It plays a major role in the regulation of the hypothalamus–pituitary–adrenocortical (HPA) axis. Despite variation in sequence, CRF peptides compromise 41 amino acids across all examined species, and they exhibit notable homologies between humans and other species with 76–95% similarities [3]. In response to stressful stimuli, hypothalamic CRF release triggers a cascade, prompting the secretion of corticotropin, which is also known as the adrenocorticotropic hormone (ACTH) from the anterior pituitary gland. Subsequent transport of corticotropin via the blood to the adrenal gland cortex instigates cortisol (hydrocortisone) release. Once cortisol reaches the normal physiological concentration, it then causes a classical negative feedback inhibition on the hypothalamus, which is very important for the regulation and homeostasis of the stress system [4]. Dysregulation of the HPA system occurs in many stress-related disorders where this negative feedback system may be impaired, resulting in elevated levels of CRF [5]. Depression is associated with CRF hypersecretion, and studies revealed that CRF mRNA has higher expression in the PVN of patients with depression and also found elevated CRF in the CSF of drug-free depressed patients [6,7] Several analogs of CRF exist in different species, and they include urocortin, sauvagine, and urotensin. They all exert their physiological and biological functions through binding to membrane-bound G-protein couple receptors (GPCRs). Two CRF receptor subtypes exist, including CRF_1_ and CRF_2_ [8,9,10]. The binding affinity of CRF to CRF_1_R is almost eight times higher relative to its binding to CRF_2_R [11]. Abnormally high levels of CRF are often implicated in a variety of stress-related diseases, including anxiety, depression, suicidal tendencies, Alzheimer’s disease, anxiety-induced relapses of drug addiction, and several other psychiatric and mental disorders [6,12]. CRF also modulates many other physiological and pathological processes of the cardiovascular, gastrointestinal, behavioral, immune, and reproductive systems [13,14,15,16,17,18,19,20,21,22,23,24,25]. The total economic burden of these diseases and disorders in the US exceeds USD 1 trillion annually. This heavy economic impediment highlights the urgency for new and effective therapeutic interventions. Thus, antagonists of the CRF receptors hold promise as potential drug candidates for ameliorating stress-related disorders such as anxiety, depression, and addictive disorders [12,26,27,28] as well as treating peripheral disorders such as Irritable Bowel Syndrome, inflammation, peptic ulcer, and many others [29,30,31,32]. Some of the recent studies demonstrated that CRF_1_R antagonists can prevent the expression of anxiety-like behavior in mice [33], improve the HPA axis negative feedback mechanism in stressed mice [34], and reduce stress-induced addictive behavior [35]. There are literally hundreds of non-peptide small-molecule CRF_1_ receptor antagonists available in the literature. Many of these compounds have demonstrated promise in animal models of stress-related disorders. Several molecules, including antalarmin, CP-326311, verucefont, and NBI-34041 (Figure 1), have been evaluated in multiple subclinical studies and also clinical studies in patients suffering from anxiety, depression, stress disorders, endometriosis, and drug addiction [36,37,38,39,40,41]. Unfortunately, none of them has been approved as a drug because they have failed in clinical trials for different reasons, including lack of proper efficacy and elevation of liver enzymes, which may indicate hepatic toxicity [42]. Because anxiety and depression are major problems affecting hundreds of millions of people and have been associated with chronic over-activation of the HPA axis, it was expected that these conditions would be the first to be treated by CRF_1_R antagonists.

However, the first clinical application of CRF_1_R antagonists was in the treatment of classic congenital adrenal hyperplasia. Yet, the potential of CRF_1_R antagonists as a treatment option for anxiety, depression, and other stress-related disorders is still an open chapter.

Recently, the recognition of CRF_1_R antagonists as a potential treatment option for congenital adrenal hyperplasia, also known as CAH [43], marks a significant advancement. Congenital adrenal hyperplasia is an autosomal recessive disorder, and it is most commonly associated with 21-hydroxylase enzyme deficiency (21OHD). This enzymatic defect leads to a decreased production of the endogenous glucocorticoid cortisol, resulting in a lack of negative feedback towards CRF secretion from the hypothalamus and ACTH from the anterior pituitary gland [44]. CAH due to 21OHD occurs in approximately 1:15,000 births, and it is associated with a myriad of devastating complications, such as atypical genitalia in female infants. Continued androgen excess during childhood and adolescence in females causes sexual precocity and virilization. Continued excessive androgen production during adulthood causes hirsutism and irregular menses in women. Both sexes with 21OHD also suffer from reduced fertility and psychiatric disorders [45]. Until recently, treatment of CAH mainly aimed to substitute the deficient steroid hormones. To suppress adrenal androgen synthesis, higher doses of glucocorticoids are required, which can often lead to growth suppression in children, iatrogenic Cushing syndrome, and metabolic disorders such as obesity, insulin resistance, and hypertension, which can increase cardiovascular risk [46,47,48].

**Figure 2:**
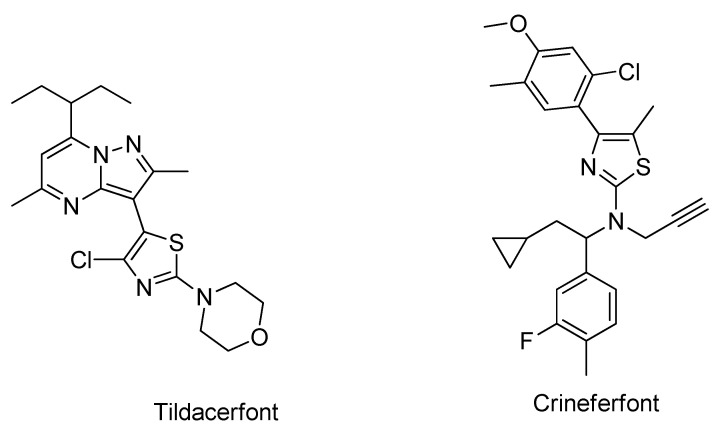
Structure of Tildacerfont and Crinecerfont.

Recently, two CRF_1_ receptor antagonists, tildacerfont and crinecerfont (Figure 2), were developed and are in clinical trials on Phase 2 and Phase 3, respectively. They have shown excellent promise in managing CAH by lowering elevated adrenal androgens [43,49,50], and this discovery has opened a new era for CRF_1_R antagonist researchers as potential drug candidates [51]. Both molecules, tildacerfont and crinecerfontare, are classified as orphan drugs [52]. Tildacerfont also showed promising effects in treating polycystic ovary syndrome. On 5 December 2023, Neurocrine Biosciences announced that it had received a breakthrough therapy designation from the U.S. Food and Drug Administration (FDA) for crinecerfont in congenital adrenal hyperplasia.

Hence, developing new molecules with diverse structural scaffolds as CRF_1_ receptor antagonists and further clinical investigations are still an active area of research that may shed more light on potential new treatment options for CAH. This new approach could help ameliorate the negative consequences of long-term supra physiological glucocorticoid treatment.

In the last decade, our group has published several papers describing the synthesis of several series of substituted pyrimidines and fused pyrimidine compounds that retained the main structural features of CRF_1_ receptor antagonists. Those series of compounds were evaluated, and several compounds have demonstrated promising binding affinity to the CRF_1_ receptors [53,54,55,56]. To continue our work in this direction, we have synthesized new analogs of our previously published lead compounds. In this manuscript, we report the synthesis, characterization, biological evaluation, and structural-activity relationship (SAR) of our most recent synthesized compounds.

## 2. Results and Discussion

### 2.1. Design Rationale

**Figure 3:**
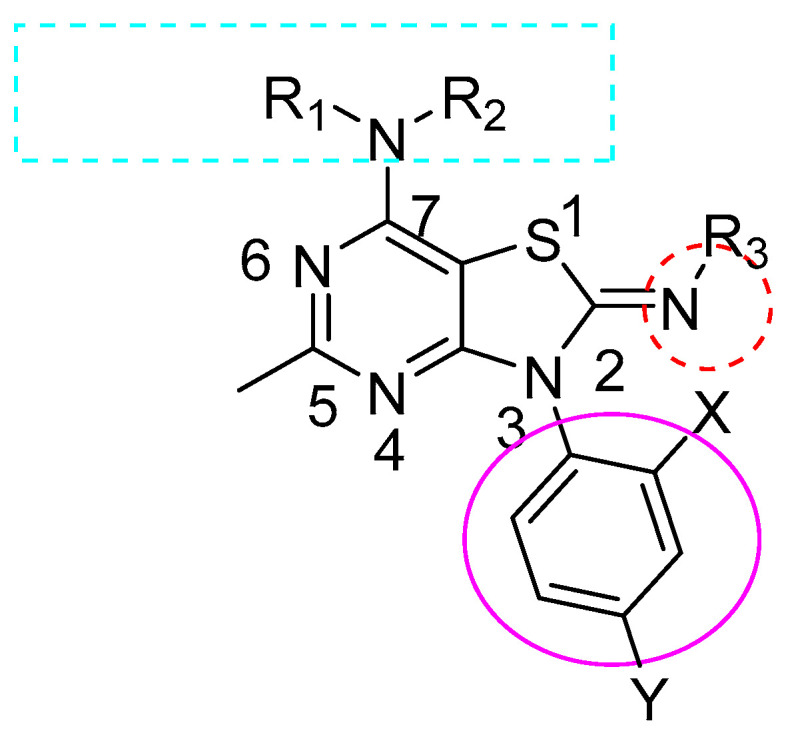
General structure of synthesized analogs.

The new set of analogs reported in this work was designed based on SAR studies of our previously prepared published compounds from the same research project [56], while keeping the basic pharmacophore. The general structure (Figure 3) was modified using 2,4-di substituted phenyl group at position-3. At least one side of the alkyl amino group at C-7 contained more than two carbons in the chain, and the N-CH3 group at the N-2 position had superior binding affinity to CRF1R.

### 2.2. Chemistry

**Figure 4:**
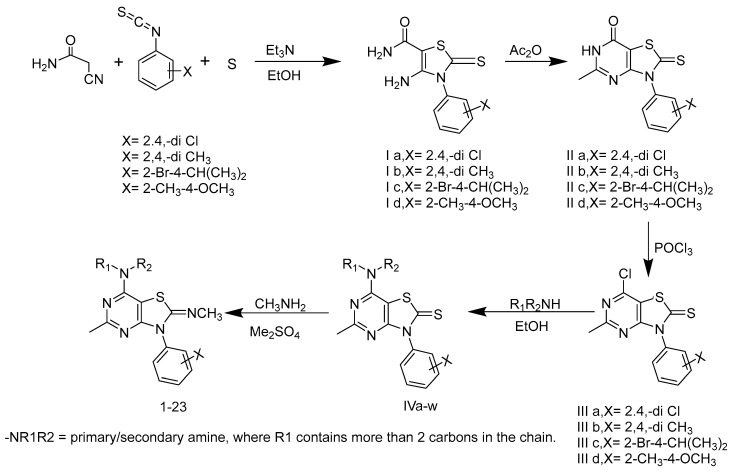
General synthetic scheme for intermediate and final target compounds.

The general synthetic scheme for the designed target compounds is described in Figure 4. The synthetic procedures for the synthesis of intermediates I–IV are reported in our previously published papers [53,54,55,57]. In brief, the selected 2,4-disubstituted phenyl isothiocyanates were allowed to react with cynoacetamide and sulfur in the presence of triethylamine following a Gewald-reaction to give the intermediate 4-amino-3-substituted phenyl-2-thioxo-2,3-dihydrothiazole-5-carboxamide (Ia-d) [58,59]. These intermediates (Ia-d) were heated in acetic anhydride at reflux temperature to undergo cyclization into the 5-methyl-3-substituted phenyl-2-thioxo-2,3-dihydrothiazolo[4,5-d]pyrimidin-7(7aH)-ones (IIa-d), as previously described by Fahmy et al. [60]. Compounds IIa-d were then subjected to a chlorination reaction using phosphorus oxychloride at reflux temperature [53,60,61] to produce the 7-chloro-5-methyl-3-substituted phenylthiazolo[4,5-d]pyrimidine-2(3H)-thione derivatives (IIIa-d). These chloro derivatives were then treated with two equivalents of the selected dialkylamine/alkylamine to give the 7-(dialkylamino/alkylamino)-5-methyl-3-substituted phenylbenzo[4,5-d]thiazole-2(3H)-thiones (IVa-w).

The final target compounds (**1**–**23**) were prepared from the 7-amino derivatives IVa-w by reaction with dimethyl sulphate, followed by reaction of the 2-methylthiazolium intermediate with methylamine to yield the 2-methylimino target compounds according to previously reported procedures [56]. By using different groups at three different positions, including substituted phenyl isothiocyanate at N-3, the alkylimino group at C-2, and the alkylamino group at C-7 (Figure 3), a series of final target compounds were synthesized as described in the methods section.

Structure details of target final compounds with different substitutions of general structure (Figure 3) and molecular weight of those compounds are listed in Table 1.

### 2.3. Biological Evaluation

An investigation of the specific binding was carried out in membrane homogenates extracted from human embryonic kidney (HEK 293) cells stably expressing CRF_1_ receptors, a standard methodology previously detailed in several of our published works [53,55]. This experimental approach closely adheres to the original Gkountelias et al. procedure [62].

**Figure 5:**
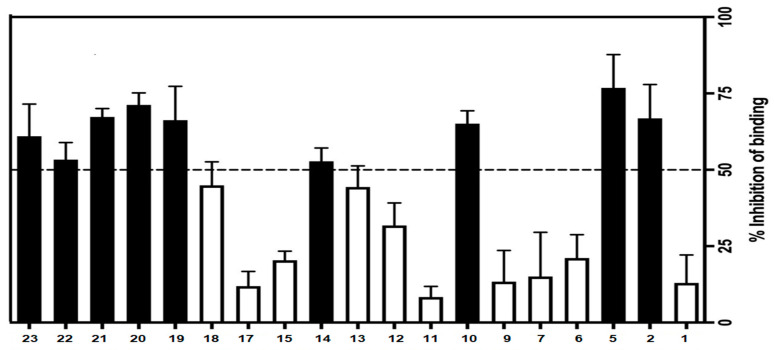
% inhibition of [^125^I]-Try^0^ sauvagine-specific binding by 100 nM of test compounds on membranes from HEK293 stably expressing human CRF_1_ receptors. In the absence of the test compound, the inhibition is 0%.

The CRF_1_ receptor binding affinities for final target compounds **1**–**23** were evaluated. First, the ability of the entire test compounds to inhibit the specific binding of the radiolabelled ligand [^125^I]-Tyr^0^ sauvagine to membranes from HEK 293 cells stably expressing the CRF_1_ receptors was evaluated at a single concentration of 100 nM. In this primary screening experiment, nine compounds (compounds **2**, **5**, **10**, **14**, and **19**–**23**) have shown their ability to inhibit more than 50% of [^125^I]-Tyr^0^ sauvagine-specific binding, while the remaining compounds showed the ability to inhibit [^125^I]-Tyr^0^ sauvagine specific binding but not more than 50% (Figure 5).

**Figure 6:**
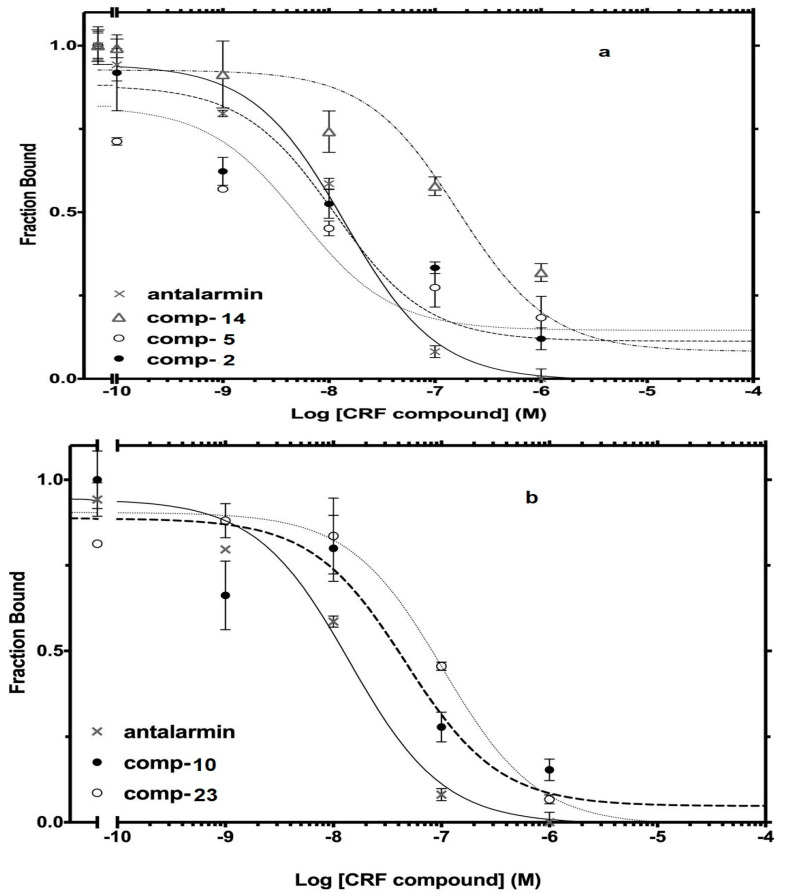

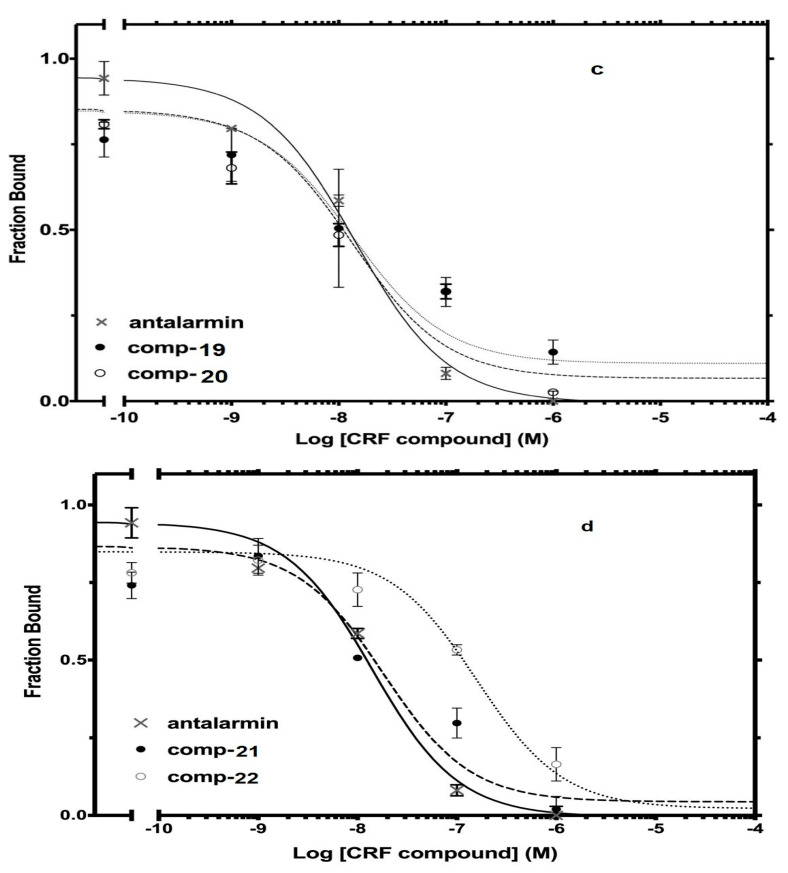
Competitive binding isotherms of compounds **2**, **5**, and **14** (**a**), compounds **10** and **23** (**b**), compounds **19** and **20** (**c**) and **21** and **22** (**d**) to human CRF_1_ receptors. Antalarmin was used as a standard drug.

The best nine compounds (lead compounds) resulting from the primary screening (compounds **2**, **5**, **10**, **14**, and **19**–**23**) were subjected to a second pharmacological characterization by determining their inhibitory ability (Log IC_50_) in competition experiments performed under equilibrium conditions in membranes from HEK 293 cells stably expressing CRF_1_ receptors. In the second assay, the inhibitory effects measured by the lead compounds were compared to those of the standard drug antalarmin. These nine lead compounds were found to inhibit [^125^I]-Tyr^0^ sauvagine binding to CRF_1_ receptors in a dose-dependent manner, with Log IC_50_ ± SE values of −8.22 ± 0.33, −7.95 ± 0.26, −7.51 ± 0.15, −6.84 ± 0.09, −7.39 ± 0.34, −8.04 ± 0.16, −7.88 ± 0.09, −7.04 ± 0.24, and −6.95 ± 0.12 for the compounds **2**, **5**, **10**, **14**, **19**, **20**, **21**, **22** and **23**, respectively, compared to the Log IC_50_ ± SE value of −7.78 ± 0.21 for the standard drug antalarmin (Figure 6). The corresponding IC_50_ (nM) values are shown in Table 2.

### 2.4. Structure-Activity Relationship (SAR) of the Lead Compounds

**Figure 7:**
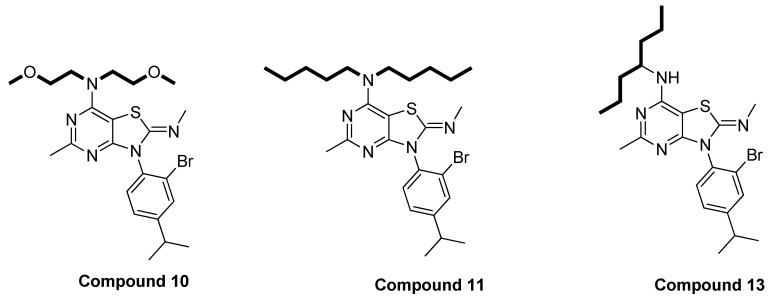
Structure of compound **11** with more than four carbons at C-7 versus other two compounds (**10** and **13**) with 3–4 carbons in the side chain at C-7 position.

The compounds prepared in this study were designed based on the SAR studies of compounds mentioned in a previously published paper [56]. This SAR aimed to narrow down the diversity of structures of compounds and to focus on definite features, hoping to obtain more potent compounds. In this series of compounds, two different positions (C-7 and N-3) were modified. The preliminary screening indicates that the groups on one side or both sides of the alkylamino group in the C-7 position should be 3–4 carbons in length for optimum inhibitory effects. Extending the carbon chain length at the C-7 position with more than four carbons can diminish the activity of the compounds in this class. For example, compound **11**, which contains five carbons on each side of the alkylamino group at the C-7 position, showed reduced activity compared to compounds **10** and **13** (Figure 7).

**Figure 8:**
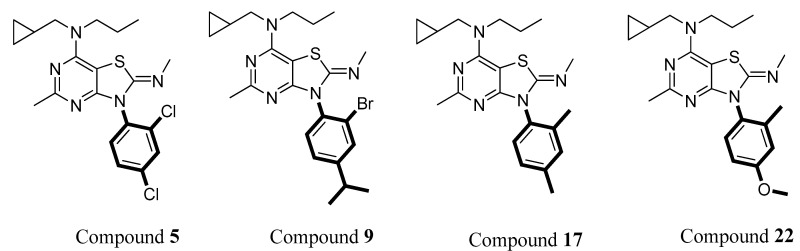
Representative compounds with different substituted phenyl groups at the N-3 position.

The study results also revealed that 2,4-dichlorophenyl and 4-methoxy-2-methylphenyl groups at the N-3 position showed better inhibitory effects than 2,4-dimethylphenyl and 2-bromo-4-isopropylphenyl. For example, compounds **5** and **22,** which have 2,4-dichlorophenyl and 4-methoxy-2-methylphenyl groups at the N-3 position, respectively, showed significantly higher inhibitory effects than compounds **9** and **17**, which contain 2-bromo-4-isopropylphenyl and 2,4-dimethylphenyl, respectively (Figure 8). Most of the compounds with a 4-methoxy-2-methylphenyl group at N-7 exhibited a better inhibitory effect, although the best active compound (compound **2**) contains 2,4-dichlorophenyl at N-3.

Based on the SAR studies of this series of compounds as well as studies of our previously published paper, it can be concluded that 2,4-dichlorophenyl or 4-methoxy-2-methylphenyl at the N-3 position, both sides of the alkylamino group at the C-7 position having 3–4 carbons, and a small group such as N-CH_3_ at the C-2 position can be optimum for this class of compound for better inhibitory effect.

### 2.5. Prediction of Physicochemical Parameters and BBB Permeability

In recent decades, in silico modeling of the physicochemical and pharmacokinetic properties of designed and synthesized compounds has been considered one of the key tools available in rational drug design and development [63]. The in silico ADME prediction permits the parallel optimization of compound efficacy and drug-like properties. This helps not only to improve the overall quality of drug candidates and the probability of their success but also to lower the cost.

To examine the possible drug-likeness of the best five compounds (compounds **2**, **5**, **19**, **20**, and **21**), in silico evaluation of some physicochemical properties such as molecular weight (MW), number of rotating bonds (RBs), number of hydrogen bond acceptors (HBAs), number of hydrogen bond donors (HBDs), calculated partition coefficient (clog P), violations of Lipinski’s rule (Vio LR), and BBB permeability were performed according to the methods described in Section 3.3.

From the five predictive models available to be used to predict the lipophilicity in the Swiss ADME software (http://www.swissadme.ch/, accessed on 30 March 2022), which are XLOGP3, WLOGP, MLOGP, SILICOS-IT, and iLOGP, we have only used MLOGP in our prediction of lipophilicity with the Swiss ADME software since it is the archetype of the topological method that is adopted from Moriguchi’s and Lipinki’s work [64]. The online “Light BBB” tool complies with most CNS drugs with an accuracy of 90% and a specificity of 0.94 [65].

The theoretical calculations of physicochemical parameters as well as the BBB permeability of the selected compounds as well as antalarmin are shown in Table 3. The theoretical results indicate that all five of the best compounds do not violate Lipinski’s rule, whereas the standard drug antalarmin has one violation.

### 2.6. Significance

CRF intricately regulates neuroendocrine functions and behavioral as well as autonomic adaptation in response to stress [66,67,68]. Chronic and overproduction of CRF could explain the pathogenesis of several severe chronic diseases, including anorexia, depression, anxiety, hypogonadism, peptic ulcers, irritable bowel syndrome, drug addiction, suicidal tendency, immunosuppression, and many other disorders [67,69].

Currently, different classes of antidepressant drugs are available for treating MDD. These drugs include tricyclic antidepressants (TCAs), monoamine oxidase inhibitors (MAOIs), selective serotonin reuptake inhibitors (SSRIs), serotonin-norepinephrine reuptake inhibitors (SNRIs), norepinephrine-dopamine reuptake inhibitors (NDRIs), and serotonin receptor antagonists with serotonin reuptake inhibition (SARI) [70]. Despite a large number of anxiolytics and anti-depressant drugs on the market, the number of patients with MDD has not only remained large but is also increasing. The annual economic burden of only MDD on society was USD 326 billion in 2018. More than 20% of patients with MDD exhibit treatment resistance to the current medications [71], and in some cases, the side effects of the current antidepressant medications may make the condition worse [72]. So, new antidepressant drugs that work through different mechanisms may represent other options for treating MDD. Several studies have revealed that CRF_1_R antagonists can be potential candidates for the treatment of MDD. CRFR antagonists can also be a novel alternative treatment option for abdominal and pelvic pain [73].

Perhaps the most important advance related to CRF_1_R antagonists was the fact that they have recently been recognized as a potential treatment option for congenital adrenal hyperplasia CAH [43], and two molecules were advanced to phase-II and phase-III clinical trials with highly promising results [74,75], with one receiving FDA approval and the second underway. In the Phase-III crinical trial, CRF_1_R antagonist crinecerfont is also a promising drug candidate for pediatric CAH [76]. The emerging therapies for CAH with CRF_1_R antagonists opened up a novel concept that can reduce the dose of glucocorticoids significantly, which helps to improve quality of life [77].

Both peptide and non-peptide molecules may be developed as CRF_1_R antagonists. However, most research focuses on small non-peptide molecules due to several reasons. Small-molecule non-peptide CRF1R antagonists are stable, cost-effective, easy to prepare, non-immunogenic, and have enhanced pharmacokinetic profiles and blood brain permeability because of their low molecular weight [78]. Peptide drugs in general suffer serious drawbacks, such as a lack of oral bioavailability and the risk of illiciting an immune response.

Some of the major pharmaceutical companies have developed many promising molecules, and some of them have advanced to clinical trials. Neurocrine Biosciences, Bristol Mayers Squibb, Pfizer, and GSK developed compounds that were advanced to clinical trials for major depression, suicidal tendency, irritable bowel syndrome, and alcoholism. But those compounds failed mainly due to a lack of efficacy or hepatotoxicity [42,79]. Some of the prominent CRF1R antagonists, including antalarmin and verucerfont (Figure 1), contain a bicyclic core ring system where the five-membered ring (pyrrolo/pyrazolo) has an un-modifiable methyl group at C-5 (antalarmin) or C-3 (verucerfont). To compare those compounds with our compounds that are presented in this paper, the best compounds reported in this paper (compounds **2**, **5**, and **19**–**21**) have N–CH_3_ group at C-2, which may facilitate metabolism through N-dealkylation followed by conjugation, and thus it may be less hepatotoxic than the other compounds that were advanced to clinical trials. However, further studies are needed to evaluate the hepatotoxicity of our compounds. Thus, the chemical scaffold we developed may potentially have an advantage over existing compounds that were advanced to clinical trials by having a modifiable C-2 position. The receptor binding studies clearly showed that some of our compounds showed superior binding affinity to CRF receptors, and the best one has 2.5-folds higher affinity than antalarmin. It is worth mentioning that, by comparing the binding kinetics of the most renowned CRF_1_ receptor antagonists evaluated by Fleck et al. [79], it appears that the best one, compound **2**, is possibly one of the top CRF_1_R antagonists ever synthesized in the history of small-molecule CRF_1_R antagonists in terms of binding affinity. In addition to this, preliminary predictions of drug-able properties demonstrated that most of our best compounds have excellent drug-able properties without violating any basic parameters of Lipinski’s rules of five.

## 3. Materials and Methods

### 3.1. Chemistry

#### 3.1.1. Materials and General Information

All chemicals used in the manuscript were purchased or sourced from chemical companies including Sigma-Aldrich (Burlington, MA, USA), Fisher (Waltham, MA, USA), Oakwood Chemicals (West Columbia, SC, USA), and TCI (Portland, OR, USA). Flash column chromatographic separation and purification were carried out using silica gel 40–60 mm, 60 Å as the stationary phase and different ratios of ethyl acetate and petroleum ether (hexanes) as the eluent. TLC plates (Sigma (Kawasaki, Japan) TLC-99577) were used for thin-layer chromatography. For visualization, the universal visualization systems of UV fluorescence at 254 nm or iodine vapor were used. Mel-Temp Laboratory apparatus was used for melting point determination. A Bruker (Billerica, MA, USA) Avance 600 MHz NMR spectrometer was utilized for ^1^H and ^13^C NMR spectroscopy. Deuterated solvents for NMR spectroscopy include DMSO-d6 and CDCl_3_. Order, multiplicity, number of protons, and signals of NMR were recorded as s (singlet), d (doublet), dd (doublet of doublet), t (triplet), dt (doublet of triplet), td (triplet of doublet), m (multiplet), br s (broad signal), q (quartet), quin (quintet), tquin (triplet of quintet), sxt (sextet), spt (septet). Chemical shifts were recorded relative to TMS, which was used as the internal standard. MestReNova 6.0.2 and Topspin 4.0.5 NMR software were used for NMR spectra analysis. Mass spectra were recorded using a Bruker Solari X MRMS (Magnetic Resonance Mass Spectrometry) instrument using the electro-spray ionization method, and Bruker Compass Data Analysis 4.2 software was utilized for data reporting.

#### 3.1.2. General Synthetic Procedure for Preparation of Intermediate Compounds (I–IV)

The intermediate compounds I–IV were obtained through four steps: First, the selected 2,4-disubstituted phenyl isothiocyanates were allowed to react with cynoacetamide and sulfur in the presence of triethylamine following the established Gewald-reaction to give the 4-amino-3-substituted phenyl-2-thioxo-2,3-dihydrothiazole-5-carboxamide compounds (Ia-d) [58,59]. Second, the thiazoline derivatives (Ia-d) were heated in acetic anhydride at reflux temperature, where they underwent cyclization into the 5-methyl-3-substituted phenyl-2-thioxo-2,3-dihydrothiazolo[4,5-d]pyrimidin-7(7aH)-ones (IIa-d), as previously described by Fahmy et al. [60]. Third, the cyclized compounds IIa-d were then subjected to a chlorination reaction using phosphorus oxychloride at reflux temperature [53,60,61] to produce the 7-chloro-5-methyl-3-substituted phenylthiazolo[4,5-d]pyrimidine-2(3H)-thione derivatives (IIIa-d). Fourth, these chloro derivatives were then treated with two equivalents of the selected dialkylamine/alkylamine to give the 7-(dialkylamino/alkylamino)-5-methyl-3-substituted phenylbenzo[4,5-d]thiazole-2(3H)-thiones (IVa-w) [53,60,61].

Since most of the intermediate compounds I-III were reported in our previously published papers, in this manuscript, we are reporting the characterization of only intermediates IVa-w (13 compounds).

##### 7-(*N*-Butyl-*N*-ethylamino)-2-thioxo-5-methyl-3-(2,4,-dichlorophenyl)thiazolo[4,5-d]pyrimidine (IVa)

Yield: Solid 67%; ^1^H NMR (600 MHz, Acetone) δ 7.79 (d, *J* = 2.2 Hz, 1H), 7.63 (dd, *J* = 8.5, 2.3 Hz, 1H), 7.58–7.56 (m, 1H), 3.71 (tt, *J* = 8.1, 4.1 Hz, 2H), 3.65–3.61 (m, 2H), 2.29 (d, *J* = 3.5 Hz, 3H), 1.71 (tt, *J* = 7.9, 6.9 Hz, 2H), 1.47–1.40 (m, 2H), 1.28 (t, *J* = 7.1 Hz, 3H), 0.99 (t, *J* = 7.4 Hz, 3H); ^13^C NMR (151 MHz, Acetone) δ 206.17, 189.44, 166.26, 160.33, 155.80, 136.73, 134.72, 134.43, 133.47, 130.95, 129.49, 96.59, 49.11, 44.50, 31.78, 25.77, 20.66, 14.25, 14.17.

##### 7-(*N*,*N*-Dipropylamino)-2-thioxo-5-methyl-3-(2,4-dichlorophenyl)thiazolo[4,5-d]pyrimidine (IVb)

Yield: Solid 70%; ^1^H NMR (600 MHz, CDCl_3_) δ 7.55 (d, *J* = 2.2 Hz, 1H), 7.38 (dd, *J* = 8.4, 2.3 Hz, 1H), 7.22–7.20 (m, 1H), 3.45–3.41 (m, 4H), 2.28 (s, 3H), 1.65–1.61 (m, 4H), 0.91 (dd, *J* = 9.3, 5.5 Hz, 6H).

##### 7-(*N*,*N*-Bis(2-methoxyethyl)amino)-2-thioxo-5-methyl-3-(2,4-dichlorophenyl)thiazolo[4,5-d]pyrimidine (IVc)

Yield: Solid 76%; ^1^H NMR (600 MHz, CDCl_3_) δ 7.61 (d, *J* = 2.3 Hz, 1H), 7.44 (dd, *J* = 8.4, 2.3 Hz, 1H), 7.29–7.26 (m, 1H), 3.90–3.85 (m, 4H), 3.63 (t, *J* = 5.7 Hz, 4H), 3.37 (s, 6H), 2.35 (s, 3H); ^13^C NMR (151 MHz, CDCl_3_) δ 188.59, 165.60, 159.47, 155.36, 136.36, 133.96, 132.85, 131.65, 130.67, 128.48, 96.78, 71.04, 59.12, 49.54, 25.70.

##### 7-(*N*,*N*-Dipentylamino)-2-thioxo-5-methyl-3-(2,4-dichlorophenyl)thiazolo[4,5-d]pyrimidine (IVd)

Yield: Solid 57%; ^1^H NMR (600 MHz, CDCl_3_) δ 7.52 (d, *J* = 2.3 Hz, 1H), 7.34 (dd, *J* = 8.5, 2.3 Hz, 1H), 7.20–7.17 (m, 1H), 3.51–3.37 (m, 4H), 2.26 (d, *J* = 1.2 Hz, 3H), 1.69–1.49 (m, 4H), 1.33–1.24 (m, 6H), 0.92–0.88 (m, 2H), 0.84 (tt, *J* = 7.2, 2.4 Hz, 6H); ^13^C NMR (151 MHz, CDCl_3_) δ 188.73, 165.72, 159.33, 155.01, 136.34, 133.99, 132.95, 131.65, 130.68, 128.46, 96.10, 49.27, 28.93, 25.73, 22.67, 22.51, 14.10.

##### 7-(*N*-Propyl-*N*-(cyclopropylmethyl))amino-2-thioxo-5-methyl-3-(2,4-dichlorophenyl)thiazolo[4,5-d]pyrimidine (IVe)

Yield: Solid 74%; ^1^H NMR (600 MHz, CDCl_3_) δ 7.55 (d, *J* = 2.3 Hz, 1H), 7.39–7.36 (m, 1H), 7.23–7.20 (m, 1H), 3.52–3.44 (m, 4H), 2.28 (d, *J* = 3.6 Hz, 3H), 1.69–1.62 (m, 2H), 1.08–1.01 (m, 1H), 0.92 (t, *J* = 7.4 Hz, 3H), 0.54–0.49 (m, 2H), 0.29–0.24 (m, 2H); ^13^C NMR (151 MHz, CDCl_3_) δ 188.85, 165.70, 159.37, 155.25, 136.40, 133.98, 132.87, 131.60, 130.72, 128.47, 96.28, 53.15, 50.99, 25.73, 22.32, 11.09, 10.29, 3.84.

##### 7-(*N*-Pentan-3-yl)amino-2-thioxo-5-methyl-3-(2,4-dichlorophenyl)thiazolo[4,5-d]pyrimidine (IVf)

Yield: Solid 62%;^1^H NMR (600 MHz, CDCl_3_) δ 7.63 (d, *J* = 2.2 Hz, 1H), 7.46 (dd, *J* = 8.5, 2.3 Hz, 1H), 7.30–7.28 (m, 1H), 4.18 (ddd, *J* = 183.6, 78.0, 45.8 Hz, 2H), 2.38 (s, 3H), 1.74–1.66 (m, 2H), 1.58–1.50 (m, 2H), 0.97 (td, *J* = 7.4, 3.5 Hz, 6H).

##### 7-(*N*-Heptan-4-yl)amino-2-thioxo-5-methyl-3-(2,4-dichlorophenyl)thiazolo[4,5-d]pyrimidine (IVg)

Yield: Solid 65%; ^1^H NMR (600 MHz, CDCl_3_) δ 7.55 (s, 1H), 7.38 (d, *J* = 7.5 Hz, 1H), 7.22 (d, *J* = 8.0 Hz, 1H), 4.07 (s, 1H), 2.30 (s, 3H), 1.55–1.27 (m, 8H), 0.87 (s, 6H); ^13^C NMR (151 MHz, CDCl_3_) δ 188.65, 166.62, 155.16, 136.56, 133.99, 132.53, 131.58, 130.78, 128.52, 51.96, 37.90, 25.65, 19.07, 14.06, 14.04.

##### 7-(*N*,*N*-Dipropylamino)-2-thioxo-5-methyl-3-(2-bromo-4-isopropylphenyl)thiazolo[4,5-d]pyrimidine (IVh)

Yield: Solid 81%; ^1^H NMR (600 MHz, CDCl_3_) δ 7.56 (d, *J* = 1.8 Hz, 1H), 7.29 (dd, *J* = 8.1, 1.8 Hz, 1H), 7.18–7.15 (m, 1H), 3.47–3.40 (m, 4H), 2.92 (dq, *J* = 13.8, 6.9 Hz, 1H), 2.29 (s, 3H), 1.67–1.60 (m, 4H), 1.25 (d, *J* = 7.0 Hz, 6H), 0.91 (t, *J* = 7.4 Hz, 6H); ^13^C NMR (151 MHz, CDCl_3_) δ 188.97, 165.67, 159.59, 155.08, 152.16, 133.31, 131.84, 130.25, 127.02, 122.47, 96.22, 50.97, 33.84, 25.83, 23.70, 23.66, 22.06, 11.16.

##### 7-(*N*,*N*-Bis(2-methoxyethyl)amino)-2-thioxo-5-methyl-3-(2-bromo-4-isopropylphenyl)thiazolo[4,5-d]pyrimidine (IVi)

Yield: semisolid 77%; ^1^H NMR (600 MHz, CDCl_3_) δ 7.54 (d, *J* = 1.9 Hz, 1H), 7.28 (dd, *J* = 8.2, 1.8 Hz, 1H), 7.16–7.14 (m, 1H), 3.80 (t, *J* = 5.7 Hz, 4H), 3.56 (t, *J* = 5.7 Hz, 4H), 3.29 (s, 6H), 2.95–2.86 (m, 1H), 2.27 (d, *J* = 7.5 Hz, 3H), 1.23 (d, *J* = 7.0 Hz, 6H); ^13^C NMR (151 MHz, CDCl_3_) δ 188.75, 165.54, 159.74, 155.32, 152.18, 133.26, 131.80, 130.28, 127.02, 122.47, 96.80, 71.12, 59.11, 49.51, 33.82, 25.78, 23.71, 23.67.

##### 7-(*N*,*N*-Dipentylamino)-2-thioxo-5-methyl-3-(2-bromo-4-isopropylphenyl)thiazolo[4,5-d]pyrimidine (IVj)

Yield: Semisolid 57%; ^1^H NMR (600 MHz, CDCl_3_) δ 7.53 (d, *J* = 1.7 Hz, 1H), 7.27 (dd, *J* = 8.1, 1.8 Hz, 1H), 7.15 (d, *J* = 8.1 Hz, 1H), 3.55–3.36 (m, 4H), 2.90 (dt, *J* = 13.8, 6.9 Hz, 1H), 2.26 (d, *J* = 9.3 Hz, 3H), 1.63–1.25 (m, 12H), 1.22 (d, *J* = 7.0 Hz, 6H), 0.85 (dd, *J* = 8.4, 5.9 Hz, 6H); ^13^C NMR (151 MHz, CDCl_3_) δ 188.88, 165.64, 159.60, 155.00, 152.09, 133.37, 131.79, 130.29, 126.99, 122.49, 96.16, 77.41, 77.20, 76.99, 49.22, 33.83, 28.94, 25.80, 23.71, 23.67, 22.66, 22.50, 14.09.

##### 7-(*N*-Propyl-*N*-(cyclopropylmethyl))amino-2-thioxo-5-methyl-3-(2-bromo-4-isopropylphenyl)thiazolo[4,5-d]pyrimidine (IVk)

Yield: Solid 78%; ^1^H NMR (600 MHz, CDCl_3_) δ 7.55 (d, *J* = 1.8 Hz, 1H), 7.29 (dd, *J* = 8.1, 1.8 Hz, 1H), 7.18–7.15 (m, 1H), 3.51–3.45 (m, 4H), 2.92 (dt, *J* = 13.8, 6.9 Hz, 1H), 2.29 (s, 3H), 1.70–1.62 (m, 2H), 1.25 (d, *J* = 7.0 Hz, 6H), 1.09–1.02 (m, 1H), 0.92 (t, *J* = 7.4 Hz, 3H), 0.53–0.49 (m, 2H), 0.27 (q, *J* = 4.8 Hz, 2H); ^13^C NMR (151 MHz, CDCl_3_) δ 188.99, 165.62, 159.64, 155.22, 152.17, 133.30, 131.84, 130.25, 127.03, 122.47, 96.37, 53.13, 50.98, 33.84, 25.82, 23.70, 23.66, 22.34, 11.11, 10.31, 3.84.

##### 7-(*N*-Pentan-3-yl)amino-2-thioxo-5-methyl-3-(2-bromo-4-isopropylphenyl)thiazolo[4,5-d]pyrimidine (IVl)

Yield: Solid 58%; ^1^H NMR (600 MHz, CDCl_3_) δ 7.56 (d, *J* = 1.8 Hz, 1H), 7.30 (dd, *J* = 8.1, 1.8 Hz, 1H), 7.18–7.16 (m, 1H), 5.27–4.09 (m, 1H), 3.84 (s, 1H), 2.92 (dq, *J* = 13.8, 6.9 Hz, 1H), 2.32 (s, 3H), 1.66–1.58 (m, 2H), 1.51–1.43 (m, 2H), 1.25 (d, *J* = 7.0 Hz, 6H), 0.90 (td, *J* = 7.4, 4.5 Hz, 6H).

##### 7-(*N*-Heptan-4-yl)amino-2-thioxo-5-methyl-3-(2-bromo-4-isopropylphenyl)thiazolo[4,5-d]pyrimidine (IVm)

Yield: Solid 55%; ^1^H NMR (600 MHz, CDCl_3_) δ 7.56 (d, *J* = 1.8 Hz, 1H), 7.30 (dd, *J* = 8.1, 1.8 Hz, 1H), 7.19–7.16 (m, 1H), 4.04 (s, 1H), 2.92 (hept, *J* = 6.9 Hz, 1H), 2.32 (s, 3H), 1.56–1.29 (m, 8H), 1.25 (d, *J* = 6.9 Hz, 6H), 0.87 (td, *J* = 7.3, 4.2 Hz, 6H).

##### 7-(*N*-Butyl-*N*-ethylamino)-2-thioxo-5-methyl-3-(2,4-dimethylphenyl)thiazolo[4,5-d]pyrimidine (IVn)

Yield: Solid 79%; ^1^H NMR (600 MHz, CDCl_3_) δ 7.22 (s, 1H), 7.19 (d, *J* = 8.0 Hz, 1H), 7.06 (dd, *J* = 7.9, 3.6 Hz, 1H), 3.64 (q, *J* = 7.1 Hz, 2H), 3.56–3.51 (m, 2H), 2.41 (s, 3H), 2.35 (s, 3H), 2.04 (s,3H), 1.67 (tt, *J* = 7.9, 6.8 Hz, 2H), 1.45–1.38 (m, 2H), 1.27 (t, *J* = 7.1 Hz, 3H), 1.00 (t, *J* = 7.4 Hz, 3H); ^13^C NMR (151 MHz, CDCl_3_) δ 188.94, 165.77, 159.86, 154.91, 139.75, 135.89, 132.99, 132.10, 128.27, 127.99, 96.27, 48.44, 43.72, 31.15, 31.14, 25.86, 21.43, 20.03, 17.66, 13.93.

##### 7-(*N*,*N*-Dipropylamino)-2-thioxo-5-methyl-3-(2,4-dimethylphenyl)thiazolo[4,5-d]pyrimidine (IVo)

Yield: Solid 74%; ^1^H NMR (600 MHz, CDCl_3_) δ 7.21 (d, *J* = 0.4 Hz, 1H), 7.19 (d, *J* = 8.0 Hz, 1H), 7.06 (dd, *J* = 7.9, 3.1 Hz, 1H), 3.54–3.48 (m, 4H), 2.40 (d, *J* = 5.3 Hz, 3H), 2.35 (s, 3H), 2.04 (d, *J* = 4.8 Hz, 3H), 1.74–1.67 (m, 4H), 0.99 (t, *J* = 7.4 Hz, 6H); ^13^C NMR (151 MHz, CDCl_3_) δ 188.91, 165.71, 159.86, 155.09, 139.74, 135.88, 132.99, 132.09, 128.26, 127.98, 96.28, 50.92, 25.86, 22.04, 21.42, 17.66, 11.13.

##### 7-(*N*,*N*-Bis(2-methoxyethyl)amino)-2-thioxo-5-methyl-3-(2,4-dimethylphenyl)thiazolo[4,5-d]pyrimidine (IVp)

Yield: Solid 82%; ^1^H NMR (600 MHz, CDCl_3_) δ 7.22 (dd, *J* = 2.6, 2.0 Hz, 1H), 7.19 (ddd, *J* = 4.9, 1.6, 0.5 Hz, 1H), 7.06–7.04 (m, 1H), 3.91–3.86 (m, 4H), 3.64 (t, *J* = 5.6 Hz, 4H), 3.38 (s, 6H), 2.41 (s, 3H), 2.35 (s, 3H), 2.04 (s, 3H); ^13^C NMR (151 MHz, CDCl_3_) δ 188.80, 165.61, 160.02, 155.31, 139.81, 135.89, 132.91, 132.11, 128.25, 128.01, 96.89, 59.09, 49.47, 25.81, 21.42, 17.66.

##### 7-(*N*-Propyl-*N*-(cyclopropylmethyl))amino-2-thioxo-5-methyl-3-(2,4-dimethylphenyl)thiazolo[4,5-d]pyrimidine (IVq)

Yield: Solid 76%; ^1^H NMR (600 MHz, CDCl_3_) δ 7.14 (s, 1H), 7.11 (d, *J* = 8.0 Hz, 1H), 7.00–6.97 (m, 1H), 3.48 (dd, *J* = 15.8, 7.4 Hz, 4H), 2.33 (s, 3H), 2.28 (s, 3H), 1.96 (s,3H), 1.69–1.62 (m, 2H), 1.08–1.01 (m, 1H), 0.92 (t, *J* = 7.4 Hz, 3H), 0.51 (q, *J* = 5.4 Hz, 2H), 0.27 (q, *J* = 5.0 Hz, 2H); ^13^C NMR (151 MHz, CDCl_3_) δ 188.97, 165.67, 159.91, 155.22, 139.80, 135.92, 133.00, 132.14, 128.30, 128.03, 96.48, 53.14, 50.98, 25.86, 22.36, 21.47, 17.70, 11.12, 10.34, 3.85.

##### 7-(*N*-Pentan-3-yl)amino-2-thioxo-5-methyl-3-(2,4-dimethylphenyl)thiazolo[4,5-d]pyrimidine (IVr)

Yield: Solid 58%; ^1^H NMR (600 MHz, CDCl_3_) δ 7.15 (s, 1H), 7.12 (d, *J* = 7.9 Hz, 1H), 6.98 (t, *J* = 6.5 Hz, 1H), 4.06–3.58 (m, 1H), 2.33 (s, 3H), 2.31 (s, 3H), 1.97 (d, *J* = 9.5 Hz, 3H), 1.66–1.58 (m, 2H), 1.51–1.43 (m, 2H), 0.90 (td, *J* = 7.4, 2.8 Hz, 6H); ^13^C NMR (151 MHz, CDCl_3_) δ 188.89, 166.37, 159.42, 155.07, 139.98, 135.94, 132.64, 132.19, 128.29, 128.08, 54.75, 27.83, 25.66, 21.46, 17.70, 10.23.

##### 7-(*N*-Heptan-4-yl)amino-2-thioxo-5-methyl-3-(2,4-dimethylphenyl)thiazolo[4,5-d]pyrimidine (IVs)

Yield: Solid 61%; ^1^H NMR (600 MHz, CDCl_3_) δ 7.14 (s, 1H), 7.11 (d, *J* = 7.9 Hz, 1H), 7.00–6.98 (m, 1H), 4.36–3.70 (m, 1H), 2.33 (s, 3H), 2.30 (s, 3H), 1.98 (s, 3H), 1.56–1.49 (m, 2H), 1.45–1.28 (m, 6H), 0.86 (td, *J* = 7.3, 2.8 Hz, 6H); ^13^C NMR (151 MHz, CDCl_3_) δ 189.84, 167.45, 160.44, 150.55, 139.98, 135.95, 132.64, 132.39, 132.19, 128.29, 128.08, 37.90, 25.63, 21.46, 19.09, 17.71, 14.07, 14.05.

##### 7-(*N*-Butyl-N-ethyl)amino-2-thioxo-5-methyl-3-(2-methyl-4-methoxylphenyl)thiazolo[4,5-d]pyrimidine (IVt)

Yield: Solid 73%; ^1^H NMR (600 MHz, CDCl_3_) δ 7.09 (d, *J* = 8.5 Hz, 1H), 6.91 (dt, *J* = 8.5, 2.8 Hz, 2H), 3.85 (s, 3H), 3.64 (q, *J* = 7.2 Hz, 2H), 3.56–3.51 (m, 2H), 2.36 (s, 3H), 2.05 (s, 3H), 1.67 (tt, *J* = 7.9, 6.7 Hz, 2H), 1.42 (dq, *J* = 14.8, 7.5 Hz, 2H), 1.27 (t, *J* = 7.1 Hz, 3H), 1.00 (t, *J* = 7.4 Hz, 3H); ^13^C NMR (151 MHz, CDCl_3_) δ 189.25, 165.78, 160.19, 159.95, 154.93, 137.68, 129.57, 128.25, 116.42, 112.45, 96.19, 55.32, 48.45, 43.72, 31.13, 25.87, 20.03, 18.07, 13.93.

##### 7-(*N*,*N*-Dipropyl)amino-2-thioxo-5-methyl-3-(2-methyl-4-methoxylphenyl)thiazolo[4,5-d]pyrimidine (IVu)

Yield: Solid 70%; ^1^H NMR (600 MHz, CDCl_3_) δ 7.01 (dd, *J* = 8.5, 2.7 Hz, 1H), 6.85–6.84 (m, 1H), 6.83–6.80 (m, 1H), 3.78 (s, 3H), 3.47–3.39 (m, 4H), 2.28 (s, 3H), 1.97 (s,, 3H), 1.67–1.59 (m, 4H), 0.91 (t, *J* = 7.4 Hz, 6H); ^13^C NMR (151 MHz, CDCl_3_) δ 189.24, 165.73, 160.21, 159.97, 155.12, 137.69, 129.59, 128.27, 116.43, 112.47, 96.24, 55.35, 50.96, 25.89, 22.07, 18.09, 11.16.

##### 7-(*N*-Propyl-*N*-(cyclopropylmethyl))amino-2-thioxo-5-methyl-3-(2-methyl-4-methoxylphenyl)thiazolo[4,5-d]pyrimidine (IVv)

Yield: Solid 82%; ^1^H NMR (600 MHz, CDCl_3_) δ 7.09 (d, *J* = 8.5 Hz, 1H), 6.92 (d, *J* = 2.6 Hz, 1H), 6.90 (dd, *J* = 8.5, 2.8 Hz, 1H), 3.84 (s, 3H), 3.56 (dd, *J* = 15.8, 7.4 Hz, 4H), 2.36 (s, 3H), 2.05 (s, 3H), 1.77–1.70 (m, 2H), 1.16–1.09 (m, 1H), 1.00 (t, *J* = 7.4 Hz, 3H), 0.61–0.56 (m, 2H), 0.34 (q, *J* = 4.8 Hz, 2H); ^13^C NMR (151 MHz, CDCl_3_) δ 189.26, 165.69, 160.21, 160.04, 155.27, 137.68, 129.59, 128.27, 116.43, 112.48, 96.37, 55.36, 53.12, 50.96, 25.90, 22.36, 18.10, 11.12, 10.34, 3.85.

##### 7-(*N*-Pentan-3-yl)amino-2-thioxo-5-methyl-3-(2-methyl-4-methoxylphenyl)thiazolo[4,5-d]pyrimidine (IVw)

Yield: Solid 55%; ^1^H NMR (600 MHz, CDCl_3_) δ 7.10 (d, *J* = 8.5 Hz, 1H), 6.92 (d, *J* = 2.8 Hz, 1H), 6.90 (dd, *J* = 8.6, 2.8 Hz, 1H), 4.97–4.12 (m, 1H), 3.85 (s, 3H), 2.39 (s, 3H), 2.06 (s, 3H), 1.74–1.65 (m, 2H), 1.58–1.50 (m, 2H), 0.97 (td, *J* = 7.4, 3.2 Hz, 6H); ^13^C NMR (151 MHz, CDCl_3_) δ 189.20, 166.62, 160.30, 155.31, 137.73, 129.60, 127.92, 116.48, 112.53, 55.36, 44.95, 27.85, 25.85, 18.10, 10.22.

#### 3.1.3. General Synthetic Procedure for the Preparation of Final Targeted Compounds

1 mmole solution of the selected **IV** in 20 mL of acetonitrile was allowed to react at reflux temperature with 3.0 mmoles of dimethyl sulfate for a period of ten hours. Additional dimethyl sulfate (3.0 mmol) was then added, and the mixture was stirred under reflux while the reaction was monitored by TLC till completion. After the reaction mixture was left to cool down to room temperature, triethylamine (12 equivalents) was added, followed by the addition of methylamine hydrochloride (12 equivalents), and the reaction mixture was continuously stirred for ten hours. After confirming the reaction completion by TLC, the solvent was removed under reduced pressure. Water was then added to the residue, and the product was extracted with ethyl acetate. After several extractions, the ethyl acetate was combined, washed with water and brine solution, and then dried over anhydrous sodium sulfate. Finally, the ethyl acetate was removed by evaporation in a rotary evaporator under reduced pressure. The remaining residue was then subjected to chromatographic purification using flash column chromatography. The solvent mixture used was ethyl acetate and hexane. Finally, the pure final 2-alkylamino compounds (**1**–**23**) were characterized by a variety of spectroscopic tools, including proton and carbon NMR and high-resolution mass spectroscopy, as listed below.

##### 7-(*N*-Butyl-*N*-ethylamino)-2-(methylimino)-5-methyl-3-(2,4-dichlorophenyl)-2,3-dihydrothiazolo[4,5-d]pyrimidine (**1**)

Yield: Solid 44%; MP: 66 °C; ^1^H NMR (600 MHz, CDCl_3_) δ 7.44 (d, *J* = 2.3 Hz, 1H), 7.25 (dd, *J* = 8.4, 2.3 Hz, 1H), 7.22–7.19 (m, 1H), 3.53 (dt, *J* = 8.3, 7.1 Hz, 2H), 3.47–3.43 (m, 2H), 2.95 (s, 3H), 2.20 (s, 3H), 1.58–1.53 (m, 2H), 1.33–1.27 (m, 2H), 1.14 (t, *J* = 7.1 Hz, 3H), 0.89 (t, *J* = 7.4 Hz, 3H); ^13^C NMR (151 MHz, CDCl_3_) δ 164.34, 158.49, 155.15, 153.45, 135.34, 134.54, 133.05, 132.34, 130.56, 128.31, 89.08, 48.19, 43.50, 40.38, 31.44, 25.82, 20.13, 14.40, 14.06; HRMS (ES^+^) calculated C_19_H_24_Cl_2_N_5_S for [M + H]^+^ 424.1123; found: 424.1128.

##### 7-(*N*,*N*-Dipropylamino)-2-(methylimino)-5-methyl-3-(2,4-dichlorophenyl)-2,3-dihydrothiazolo[4,5-d]pyrimidine (**2**)

Yield: Solid 48%; MP: 66 °C; ^1^H NMR (600 MHz, CDCl_3_) δ 7.44 (d, *J* = 2.2 Hz, 1H), 7.26 (dd, *J* = 8.4, 2.3 Hz, 1H), 7.21 (d, *J* = 8.4 Hz, 1H), 3.44–3.40 (m, 4H), 2.95 (s, 3H), 2.20 (s, 3H), 1.62–1.56 (m, 4H), 0.87 (t, *J* = 7.4 Hz, 6H); ^13^C NMR (151 MHz, CDCl_3_) δ 164.27, 158.44, 155.30, 153.55, 135.36, 134.49, 132.94, 132.25, 130.58, 128.32, 89.10, 50.73, 40.32, 25.78, 22.37, 11.18..; HRMS (ES^+^) calculated for C_19_H_24_Cl_2_N_5_S [M + H]^+^: 424.1123; found: 424.1133.

##### 7-(*N*,*N*-Bis(2-methoxyethyl)amino)-2-(methylimino)-5-methyl-3-(2,4-dichlorophenyl)-2,3-dihydrothiazolo[4,5-d]pyrimidine (**3**)

Yield: Solid 50%; MP: 88–89 °C; ^1^H NMR (600 MHz, CDCl_3_) δ 7.48 (d, *J* = 2.3 Hz, 1H), 7.30 (dd, *J* = 8.4, 2.3 Hz, 1H), 7.24–7.22 (m, 1H), 3.81 (t, *J* = 5.9 Hz, 4H), 3.56 (t, *J* = 5.8 Hz, 4H), 3.31 (s, 6H), 2.97 (s, 3H), 2.22 (s, 3H); ^13^C NMR (151 MHz, CDCl_3_) δ 164.27, 158.65, 155.28, 153.14, 135.43, 134.44, 132.85, 132.14, 130.69, 130.54, 128.28, 89.64, 71.79, 59.18, 49.48, 40.44, 25.82; HRMS (ES^+^) calculated for C_19_H_24_Cl_2_N_5_O_2_S [M + H]^+^: 456.1022; found: 456.1028.

##### 7-(*N*,*N*-Dipentylamino)-2-(methylimino)-5-methyl-3-(2,4-dichlorophenyl)-2,3-dihydrothiazolo[4,5-d]pyrimidine (**4**)

Yield: Solid 49%; MP: 72–74 °C; ^1^H NMR (600 MHz, CDCl_3_) δ 7.46–7.43 (m, 1H), 7.27–7.24 (m, 1H), 7.21 (d, *J* = 8.5 Hz, 1H), 3.52–3.40 (m, 4H), 2.97–2.94 (m, 3H), 2.20 (s, 3H), 1.61–1.43 (m, 4H), 1.33–0.89 (m, 8H), 0.84 (ddd, *J* = 7.5, 5.4, 3.3 Hz, 6H); ^13^C NMR (151 MHz, CDCl_3_) δ 164.30, 158.49, 155.25, 153.46, 135.32, 134.54, 133.06, 132.31, 130.56, 128.30, 89.08, 49.01, 40.36, 29.00, 25.75, 22.53, 14.14; HRMS (ES^+^) calculated for C_23_H_32_Cl_2_N_5_S [M + H]^+^: 480.1749; found: 480.1754.

##### 7-(*N*-Propyl-*N*-(cyclopropylmethyl))amino-2-(methylimino)-5-methyl-3-(2,4-dichlorophenyl)-2,3-dihydrothiazolo[4,5-d]pyrimidine (**5**)

Yield: Solid 52%; MP: 54–56 °C; ^1^H NMR (600 MHz, CDCl_3_) δ 7.47 (d, *J* = 2.2 Hz, 1H), 7.29 (dd, *J* = 8.4, 2.2 Hz, 1H), 7.23 (d, *J* = 8.4 Hz, 1H), 3.51 (dd, *J* = 9.1, 6.7 Hz, 2H), 3.45 (d, *J* = 6.7 Hz, 2H), 2.98 (s, 3H), 2.22 (s, 3H), 1.67–1.61 (m, 2H), 1.08–1.01 (m, 1H), 0.90 (t, *J* = 7.4 Hz, 3H), 0.50–0.46 (m, 2H), 0.24 (q, *J* = 4.9 Hz, 2H); ^13^C NMR (151 MHz, CDCl_3_) δ 164.25, 158.53, 155.44, 153.50, 135.37, 134.49, 132.96, 132.26, 130.58, 128.32, 89.31, 52.91, 50.67, 40.35, 25.78, 22.56, 11.14, 10.64, 3.77; HRMS (ES^+^) calculated for C_20_H_24_Cl_2_N_5_S [M + H]^+^: 436.1123; found: 436.1131.

##### 7-(*N*-Pentan-3-yl)amino-2-(methylimino)-5-methyl-3-(2,4-dichlorophenyl)-2,3-dihydrothiazolo[4,5-d]pyrimidine (**6**)

Yield: Solid 44%; MP: 88–89 °C; ^1^H NMR (600 MHz, CDCl_3_) δ 7.49 (d, *J* = 2.3 Hz, 1H), 7.31 (dd, *J* = 8.4, 2.3 Hz, 1H), 7.25 (d, *J* = 8.4 Hz, 1H), 3.93 (s, 1H), 2.99 (s, 3H), 2.79 (dd, *J* = 21.2, 17.3 Hz, 1H), 2.26 (s, 3H), 1.64–1.56 (m, 2H), 1.48–1.41 (m, 2H), 0.89 (td, *J* = 7.2, 3.2 Hz, 6H); ^13^C NMR (151 MHz, CDCl_3_) δ 165.26, 157.86, 155.41, 153.07, 135.54, 134.47, 132.66, 132.15, 130.63, 128.36, 89.81, 54.14, 40.85, 27.88, 25.79, 10.13, 10.12; HRMS (ES^+^) calculated for C_18_H_22_Cl_2_N_5_S [M + H]^+^: 410.0967; found: 410.0977.

##### 7-(*N*-Heptan-4-yl)amino-2-(methylimino)-5-methyl-3-(2,4-dichlorophenyl)-2,3-dihydrothiazolo[4,5-d]pyrimidine (**7**)

Yield: Solid 42%; MP: 106 °C; ^1^H NMR (600 MHz, CDCl_3_) δ 7.48 (d, *J* = 2.3 Hz, 1H), 7.30 (dd, *J* = 8.4, 2.3 Hz, 1H), 7.24 (d, *J* = 8.5 Hz, 1H), 4.12 (s, 1H), 2.97 (s, 3H), 2.25 (s, 3H), 1.53–1.46 (m, 2H), 1.42–1.25 (m, 7H), 0.86 (td, *J* = 7.2, 3.0 Hz, 6H); ^13^C NMR (151 MHz, CDCl_3_) δ 165.27, 157.81, 155.32, 153.08, 135.51, 134.49, 132.70, 132.18, 130.62, 128.34, 89.72, 60.43, 40.86, 38.43, 38.11, 25.80, 19.06, 19.04, 14.15, 14.13; HRMS (ES^+^) calculated for C_20_H_26_Cl_2_N_5_S [M + H]^+^: 438.1280; found: 438.1288.

##### 7-(*N*,*N*-Dipropylamino)-2-(methylimino)-5-methyl-3-(2-bromo-4-isopropylphenyl)-2,3-dihydrothiazolo[4,5-d]pyrimidine (**8**)

Yield: Solid 55%; MP: 60–62 °C; ^1^H NMR (600 MHz, CDCl_3_) δ 7.44 (s, 1H), 7.17–7.14 (m, 2H), 3.44–3.35 (m, 4H), 2.94 (s, 3H), 2.83–2.77 (m, 1H), 2.17 (s, 3H), 1.60–1.53 (m, 4H), 1.15 (d, *J* = 7.0 Hz, 6H), 0.83 (t, *J* = 7.4 Hz, 6H); ^13^C NMR (151 MHz, CDCl_3_) δ 164.18, 158.91, 155.29, 153.73, 150.91, 133.53, 131.75, 131.23, 126.99, 123.41, 89.12, 53.67, 50.75, 40.50, 33.80, 25.82, 23.77, 22.47, 11.25; HRMS (ES^+^) calculated for C_22_H_31_BrN_5_S [M + H]^+^: 476.1478; found: 476.1484.

##### 7-(*N*-Propyl-*N*-(cyclopropylmethyl))amino-2-(methylimino)-5-methyl-3-(2-bromo-4-isopropylphenyl)-2,3-dihydrothiazolo[4,5-d]pyrimidine (**9**)

Yield: Solid 50%; MP: 83–84 °C; ^1^H NMR (600 MHz, CDCl_3_) δ 7.48 (d, *J* = 1.6 Hz, 1H), 7.20 (dd, *J* = 8.1, 1.7 Hz, 1H), 7.18 (d, *J* = 8.1 Hz, 1H), 3.53–3.47 (m, 2H), 3.46–3.40 (m, 2H), 2.98 (s, 3H), 2.87–2.81 (m, 1H), 2.21 (s, 3H), 1.67–1.60 (m, 2H), 1.19 (d, *J* = 7.1 Hz, 6H), 1.07–1.00 (m, 1H), 0.88 (dd, *J* = 13.8, 6.3 Hz, 3H), 0.49–0.44 (m, 2H), 0.23 (q, *J* = 4.8 Hz, 2H); ^13^C NMR (151 MHz, CDCl_3_) δ 164.18, 158.94, 155.42, 153.92, 150.99, 133.41, 131.78, 131.01, 126.99, 123.32, 89.36, 52.90, 50.66, 40.54, 33.79, 25.88, 23.75, 23.72, 22.59, 11.18, 10.68, 3.81; HRMS (ES^+^) calculated for C_23_H_31_BrN_5_S [M + H]^+^: 488.1478; found: 488.1487.

##### 7-(*N*,*N*-Bis(2-methoxyethyl)amino)-2-(methylimino)-5-methyl-3-(2-bromo-4-isopropylphenyl)-2,3-dihydrothiazolo[4,5-d]pyrimidine (**10**)

Yield: Solid 47%; MP: 76–77 °C; ^1^H NMR (600 MHz, CDCl_3_) δ 7.49 (d, *J* = 1.8 Hz, 1H), 7.22 (dd, *J* = 8.2, 1.8 Hz, 1H), 7.20–7.17 (m, 1H), 3.81 (t, *J* = 5.9 Hz, 4H), 3.56 (t, *J* = 5.8 Hz, 4H), 3.30 (s, 6H), 2.98 (s, 3H), 2.85 (dt, *J* = 18.3, 6.9 Hz, 1H), 2.22 (s, 3H), 1.20 (d, *J* = 6.9 Hz, 6H); ^13^C NMR (151 MHz, CDCl_3_) δ 164.23, 159.02, 155.24, 153.73, 151.13, 147.88, 133.21, 131.74, 130.97, 127.07, 123.23, 89.65, 71.71, 58.78, 49.29, 40.53, 33.77, 26.13, 23.79; HRMS (ES^+^) calculated for C_22_H_31_BrN_5_O_2_S [M + H]^+^: 508.13763554; found: 508.1384.

##### 7-(*N*,*N*-Dipentylamino)-2-(methylimino)-5-methyl-3-(2-bromo-4-isopropylphenyl)-2,3-dihydrothiazolo[4,5-d]pyrimidine (**11**)

Yield: Solid 60%; ^1^H NMR (600 MHz, CDCl_3_) δ 7.49 (d, *J* = 1.6 Hz, 1H), 7.24–7.21 (m, 1H), 7.20 (dd, *J* = 8.1, 1.6 Hz, 1H), 3.53–3.42 (m, 4H), 3.00–2.98 (m, 3H), 2.86 (dt, *J* = 13.8, 6.9 Hz, 1H), 2.22 (d, *J* = 0.7 Hz, 3H), 1.63–1.45 (m, 4H), 1.34–1.24 (m, 6H), 1.20 (d, *J* = 7.0 Hz, 6H), 0.92–0.89 (m, 2H), 0.88–0.84 (m, 6H); ^13^C NMR (151 MHz, CDCl_3_) δ 164.22, 158.85, 155.21, 154.07, 150.97, 133.39, 131.81, 130.94, 127.01, 123.27, 89.12, 48.94, 40.54, 33.77, 28.99, 28.94, 28.79, 26.25, 25.88, 25.85, 23.72, 23.69, 22.72, 22.51, 16.92, 14.12; HRMS (ES^+^) calculated for C_26_H_39_BrN_5_S [M + H]^+^: 532.2104; found: 532.2107.

##### 7-(*N*-Pentan-3-yl)amino-2-(methylimino)-5-methyl-3-(2-bromo-4-isopropylphenyl)-2,3-dihydrothiazolo[4,5-d]pyrimidine (**12**)

Yield: Solid 48%; MP: 77–78 °C; ^1^H NMR (600 MHz, CDCl_3_) δ 7.49 (d, *J* = 1.6 Hz, 1H), 7.22 (dd, *J* = 8.1, 1.7 Hz, 1H), 7.20 (d, *J* = 8.0 Hz, 1H), 3.93 (s, 1H), 2.98 (s, 3H), 2.88–2.83 (m, 1H), 2.25 (s, 3H), 1.62–1.53 (m, 2H), 1.47–1.39 (m, 2H), 1.19 (d, *J* = 7.0 Hz, 6H), 0.88 (td, *J* = 7.5, 4.6 Hz, 6H); ^13^C NMR (151 MHz, CDCl_3_) δ 165.19, 158.22, 155.37, 153.64, 151.23, 133.09, 131.83, 130.93, 127.02, 123.27, 89.88, 53.87, 40.99, 33.78, 27.86, 25.85, 23.71, 23.69, 10.17, 10.14; HRMS (ES^+^) calculated for C_21_H_29_BrN_5_S [M + H]^+^: 462.1321; found: 462.1329.

##### 7-(*N*-Heptan-4-yl)amino-2-(methylimino)-5-methyl-3-(2-bromo-4-isopropylphenyl)-2,3-dihydrothiazolo[4,5-d]pyrimidine (**13**)

Yield: Solid 46%; MP: 65–66 °C; ^1^H NMR (600 MHz, CDCl_3_) δ 7.49 (d, *J* = 1.4 Hz, 1H), 7.22–7.19 (m, 2H), 4.12 (s, 1H), 2.98 (s, 3H), 2.87–2.82 (m, 1H), 2.24 (s, 3H), 1.52–1.44 (m, 2H), 1.40–1.27 (m, 6H), 1.19 (d, *J* = 6.9 Hz, 6H), 0.85 (td, *J* = 7.2, 4.3 Hz, 6H); ^13^C NMR (151 MHz, CDCl_3_) δ 165.19, 158.18, 155.28, 153.64, 151.16, 133.13, 131.87, 131.82, 130.98, 130.93, 130.88, 127.06, 127.01, 126.96, 123.29, 89.74, 41.03, 38.07, 33.78, 26.22, 26.04, 25.77, 23.92, 23.72, 23.53, 19.07, 14.17; HRMS (ES^+^) calculated for C_23_H_33_BrN_5_S [M + H]^+^: 490.1634; found: 490.1639.

##### 7-(*N*-Butyl-*N*-ethylamino)-2-(methylimino)-5-methyl-3-(2,4-dimethylphenyl)-2,3-dihydrothiazolo[4,5-d]pyrimidine (**14**)

Yield: Semisolid 44%; ^1^H NMR (600 MHz, CDCl_3_) δ 7.27 (s, 1H), 7.23 (dd, *J* = 8.5, 1.7 Hz, 2H), 3.77 (q, *J* = 7.1 Hz, 2H), 3.71–3.68 (m, 2H), 3.19 (s, 3H), 2.47 (s, 3H), 2.44 (s, 3H), 2.22 (s, 3H), 1.83–1.77 (m, 2H), 1.57–1.50 (m, 2H), 1.38 (dd, *J* = 8.5, 5.6 Hz, 3H), 1.13 (t, *J* = 7.4 Hz, 3H); ^13^C NMR (151 MHz, CDCl_3_) δ 164.38, 159.39, 155.18, 154.31, 138.65, 136.38, 133.07, 132.08, 132.00, 129.17, 127.88, 89.08, 48.21, 43.52, 40.63, 40.60, 31.56, 25.70, 20.21, 17.96, 17.53, 14.29; HRMS (ES^+^) calculated for C_21_H_30_N_5_S [M + H]^+^: 384.2216; found: 384.2229.

##### 7-(*N*,*N*-Dipropylamino)-2-(methylimino)-5-methyl-3-(2,4-dimethylphenyl)-2,3-dihydrothiazolo[4,5-d]pyrimidine (**15**)

Yield: Semisolid 42%; ^1^H NMR (600 MHz, CDCl_3_) δ 7.27 (s, 1H), 7.26–7.22 (m, 2H), 3.71–3.63 (m, 4H), 3.20 (s, 3H), 2.47 (s, 3H), 2.45 (s, 3H), 2.23 (s, 3H), 1.87–1.80 (m, 4H), 1.14–1.08 (m, 6H); ^13^C NMR (151 MHz, CDCl_3_) δ 164.33, 159.42, 155.36, 154.21, 138.63, 136.39, 133.09, 132.03, 129.16, 127.88, 89.12, 60.37, 50.83, 40.59, 26.01, 22.56, 21.42, 21.04, 17.98, 14.36, 11.31; HRMS (ES^+^) calculated for C_21_H_29_N_5_NaS [M + Na]^+^: 406.2035; found: 406.2048.

##### 7-(*N*,*N*-Bis(2-methoxyethyl)amino)-2-(methylimino)-5-methyl-3-(2,4-dimethylphenyl)-2,3-dihydrothiazolo[4,5-d]pyrimidine (**16**)

Yield: Solid 30%; MP: 103–104 °C; ^1^H NMR (600 MHz, CDCl_3_) δ 7.07 (s, 1H), 7.04 (d, *J* = 8.1 Hz, 1H), 7.01 (d, *J* = 7.9 Hz, 1H), 3.81 (t, *J* = 5.9 Hz, 4H), 3.56 (t, *J* = 5.9 Hz, 4H), 3.31 (s, 6H), 2.96 (s, 3H), 2.27 (s, 3H), 2.21 (s, 3H), 1.99 (s, 3H); ^13^C NMR (151 MHz, CDCl_3_) δ 164.30, 159.46, 155.22, 154.07, 138.79, 136.26, 132.75, 132.04, 128.93, 127.89, 89.60, 71.75, 59.09, 49.28, 40.60, 25.90, 21.37, 17.88; HRMS (ES^+^) calculated for C_21_H_29_N_5_NaO_2_S [M + Na]^+^: 438.1934; found: 438.1936.

##### 7-(*N*-Propyl-*N*-(cyclopropylmethyl))amino-2-(methylimino)-5-methyl-3-(2,4-dimethylphenyl)-2,3-dihydrothiazolo[4,5-d]pyrimidine (**17**)

Yield: Solid 50%; MP: 61–63 °C; ^1^H NMR (600 MHz, CDCl_3_) δ 7.01 (s, 1H), 7.00–6.96 (m, 2H), 3.50–3.44 (m, 2H), 3.41 (d, *J* = 6.7 Hz, 2H), 2.93 (s, 3H), 2.21 (s, 3H), 2.18 (s, 3H), 1.96 (s, 3H), 1.60 (dq, *J* = 15.1, 7.3 Hz, 2H), 1.04–0.97 (m, 1H), 0.85 (t, *J* = 7.5 Hz, 3H), 0.45–0.40 (m, 2H), 0.20 (q, *J* = 4.9 Hz, 2H); ^13^C NMR (151 MHz, CDCl_3_) δ 164.28, 159.43, 155.45, 154.25, 138.67, 136.38, 133.03, 132.04, 129.12, 127.89, 89.31, 52.97, 50.74, 40.62, 25.98, 22.70, 21.43, 17.96, 11.26, 10.82, 3.91, 3.90; HRMS (ES^+^) calculated for C_22_H_30_N_5_S [M + H]^+^: 396.2216; found: 396.2227.

##### 7-(*N*-Pentan-3-yl)amino-2-(methylimino)-5-methyl-3-(2,4-dimethylphenyl)-2,3-dihydrothiazolo[4,5-d]pyrimidine (**18**)

Yield: Solid 48%; MP: 66 °C; ^1^H NMR (600 MHz, CDCl_3_) δ 7.04 (s, 1H), 7.02–6.98 (m, 2H), 4.24 (s, 1H), 3.93 (s, 1H), 2.94 (s, 3H), 2.23 (s, 6H), 1.99 (s, 3H), 1.54 (td, *J* = 13.5, 6.6 Hz, 2H), 1.39 (dtd, *J* = 12.4, 7.3, 5.1 Hz, 2H), 0.85 (td, *J* = 7.4, 4.2 Hz, 6H); HRMS (ES^+^) calculated for C_20_H_28_N_5_S [M + H]^+^: 370.2059; found: 370.2072.

##### 7-(*N*-Heptan-4-yl)amino-2-(methylimino)-5-methyl-3-(2,4-dimethylphenyl)-2,3-dihydrothiazolo[4,5-d]pyrimidine (**19**)

Yield: Semisolid 46%; ^1^H NMR (600 MHz, CDCl_3_) δ 7.05 (d, *J* = 0.7 Hz, 1H), 7.03–6.99 (m, 2H), 4.22–3.99 (m, 2H), 2.95 (s, 3H), 2.24 (s, 3H), 2.23 (s, 3H), 2.00 (s, 3H), 1.54–1.24 (m, 8H), 0.84 (td, *J* = 7.2, 3.4 Hz, 6H); ^13^C NMR (151 MHz, CDCl_3_) δ 165.25, 158.60, 155.26, 154.07, 138.84, 136.33, 132.67, 132.06, 128.97, 127.88, 89.66, 60.43, 41.04, 38.19, 38.17, 25.94, 21.37, 19.11, 19.09, 17.92, 14.19, 14.17; HRMS (ES^+^) calculated for C_22_H_32_N_5_S [M + H]^+^: 398.2372; found: 398.2383.

##### 7-(*N*-Butyl-N-ethylamino)-2-(methylimino)-5-methyl-3-(2-methyl-4-methoxylphenyl)-2,3-dihydrothiazolo[4,5-d]pyrimidine (**20**)

Yield: Semisolid 36%; ^1^H NMR (600 MHz, CDCl_3_) δ 7.02 (d, *J* = 8.6 Hz, 1H), 6.74 (d, *J* = 2.2 Hz, 1H), 6.71 (dd, *J* = 8.6, 2.6 Hz, 1H), 3.65 (s, 3H), 3.52 (dd, *J* = 14.1, 6.9 Hz, 2H), 3.47–3.41 (m, 2H), 2.94 (s, 3H), 2.20 (s, 3H), 1.98 (s, 3H), 1.55 (dt, *J* = 15.2, 7.7 Hz, 2H), 1.33–1.25 (m, 2H), 1.13 (t, *J* = 7.1 Hz, 3H), 0.88 (t, *J* = 7.5 Hz, 3H); ^13^C NMR (151 MHz, CDCl_3_) δ 164.34, 159.60, 159.43, 155.13, 154.57, 138.03, 130.26, 128.30, 116.37, 112.41, 88.99, 55.25, 48.16, 43.47, 40.56, 31.50, 25.97, 20.15, 18.28, 14.44, 14.09; HRMS (ES^+^) calculated for C_21_H_30_N_5_OS [M + H]^+^: 400.2165; found: 400.2176.

##### 7-(*N*,*N*-Dipropylamino)-2-(methylimino)-5-methyl-3-(2-methyl-4-methoxylphenyl)-2,3-dihydrothiazolo[4,5-d]pyrimidine (**21**)

Yield: Semisolid 51%; ^1^H NMR (600 MHz, CDCl_3_) δ 7.01 (d, *J* = 8.6 Hz, 1H), 6.73 (d, *J* = 2.7 Hz, 1H), 6.70 (dd, *J* = 8.6, 2.8 Hz, 1H), 3.65 (d, *J* = 4.8 Hz, 3H), 3.45–3.37 (m, 4H), 2.93 (s, 3H), 2.19 (s, 3H), 1.97 (d, *J* = 6.4 Hz, 3H), 1.57 (dq, *J* = 15.1, 7.4 Hz, 4H), 0.85 (t, *J* = 7.5 Hz, 6H); ^13^C NMR (151 MHz, CDCl_3_) δ 164.27, 159.60, 159.44, 155.29, 154.48, 138.01, 130.25, 128.29, 116.34, 112.39, 89.01, 55.23, 50.75, 40.51, 25.95, 22.47, 18.26, 11.24; HRMS (ES^+^) calculated for C_21_H_30_N_5_OS [M + H]^+^: 400.2165; found: 400.2176.

##### 7-(*N*-Propyl-*N*-(cyclopropylmethyl))amino-2-(methylimino)-5-methyl-3-(2-methyl-4-methoxylphenyl)-2,3-dihydrothiazolo[4,5-d]pyrimidine (**22**)

Yield: Solid 54%; MP: 89–91 °C; ^1^H NMR (600 MHz, CDCl_3_) δ 7.02 (d, *J* = 8.6 Hz, 1H), 6.74 (d, *J* = 2.8 Hz, 1H), 6.71 (dd, *J* = 8.6, 2.9 Hz, 1H), 3.65 (s, 3H), 3.51–3.46 (m, 2H), 3.42 (d, *J* = 6.7 Hz, 2H), 2.94 (s, 3H), 2.20 (s, 3H), 1.98 (s, 3H), 1.61 (dq, *J* = 15.1, 7.4 Hz, 2H), 1.05–0.98 (m, 1H), 0.86 (t, *J* = 7.5 Hz, 3H), 0.46–0.41 (m, 2H), 0.21 (q, *J* = 4.8 Hz, 2H); ^13^C NMR (151 MHz, CDCl_3_) δ 164.24, 159.60, 159.50, 155.41, 154.48, 138.02, 130.25, 128.28, 116.36, 112.41, 89.22, 55.26, 52.93, 50.69, 40.57, 25.95, 22.64, 18.28, 11.21, 10.76, 3.86, 3.84; HRMS (ES^+^) calculated for C_22_H_30_N_5_OS [M + H]^+^: 412.2165; found: 412.2175.

##### 7-(*N*-Pentan-3-yl)amino-2-(methylimino)-5-methyl-3-(2-methyl-4-methoxylphenyl)-2,3-dihydrothiazolo[4,5-d]pyrimidine (**23**)

Yield: Solid 48%; MP: 41 °C; ^1^H NMR (600 MHz, CDCl_3_) δ 7.03 (d, *J* = 8.6 Hz, 1H), 6.74 (d, *J* = 2.9 Hz, 1H), 6.70 (dd, *J* = 8.6, 2.9 Hz, 1H), 4.35 (d, *J* = 7.5 Hz, 1H), 4.01–3.94 (m, 1H), 3.64 (s, 3H), 2.92 (s, 3H), 2.22 (s, 3H), 1.98 (s, 3H), 1.57–1.48 (m, 2H), 1.41–1.32 (m, 2H), 0.88–0.78 (m, 6H); ^13^C NMR (151 MHz, CDCl_3_) δ 165.19, 159.67, 158.65, 155.40, 154.37, 138.06, 130.24, 128.02, 116.35, 112.37, 89.79, 60.40, 40.93, 27.83, 25.92, 21.02, 18.22, 14.25, 10.25; HRMS (ES^+^) calculated for C_20_H_28_N_5_OS [M + H]^+^: 386.2009; found: 386.2021.

### 3.2. Biological Evaluation

In this protocol, a radiolabeled agonist, which is [^125^I]-Tyr^0^-sauvagine is used. The cell culture medium that was used to grow these cells consisted of DMEM/F12 (1:1) supplemented with 10% bovine calf serum, 3.15 g/L glucose, and 300 mg/mL of the antibiotic geneticin. The cells were then incubated at a temperature of 37 °C with 5% CO_2_. After a suitable time was given to grow the expected number of cells to the required cell density, the cells were washed with phosphate-buffered saline (PBS) at a neutral pH of 7.2–7.3. This was followed by treatment with 2 mM EDTA containing PBS (PBS/EDTA). The suspension of cells was pitted under centrifugation at a speed of 1000× *g* for a period of 5 min, after which the resulting pellets were allowed to be homogenized in 1.5 mL of buffer H with the following composition: 20 mM HEPES, 10 mM MgCl_2_, 2 mM EGTA, 0.2 mg/mL bacitracin antibiotic, and 0.93 mg/mL aprotinin at pH 7.2 at 4 °C. The homogenizer used was a Janke & Kunkel IKA Ultra Turrax T25 homogenizer (IKA Werke GmbH & Co. KG, Staufen, Germany). The homogenates were then put into a centrifuge to be centrifuged at a speed of 16,000× *g*, for a period of 10 min, at 4 °C. The resulting membrane pellets were again suspended in a homogenizer in 1 mL of buffer B, which is buffer H containing BSA (0.1%) at a pH of 7.2 at 20 °C. After that, the membrane suspensions were diluted using buffer B and 50 μL aliquots of the suspensions and 20–25 pM of the radiolabeled ligand [^125^I]-Tyr^0^sauvagine with (test) and then without (positive control) the compounds to be evaluated at a single concentration of 100 nM.

A secondary assay was carried out to determine the competitive binding affinities of the best compounds chosen from the first assay. In this competitive binding assay, different and increasing concentrations of the test compounds were used. The final volume of the tubes was adjusted to 0.2 mL using buffer B (the composition mentioned earlier). The test mixtures were then incubated at a temperature of 21 °C for a period of two hours. Then it was filtered through presoaked Whatman 934AH filter papers (in a solution of 0.3% polyethylene imine for 1 h at a temperature of 4 °C). The filter papers were then washed with a solution of 0.5 mL of ice-cold PBS containing 0.01% Triton X-100, and the washing process was repeated three times at pH 7.1. To measure the radioactivity, a gamma radiation counter was utilized. The quantity of membranes used was adjusted to make sure that the specific binding is always equal to or less than 10% of the total concentration of the added radiolabeled sauvagine. The specific [^125^I]-Tyr^0^-sauvagine binding was defined as the total binding minus the non-specific binding in the presence of 1000 nM of the standard antagonist Antalarmin. Non-linear regression analysis was used for data analysis, and Log IC_50_ values for the test compounds in comparison to the standard antagonist antalarmin were calculated by fitting the data from the competition studies into a one-site competition model. The statistical analysis was performed using Prism 4.0 (GraphPad Software, San Diego, CA, USA).

### 3.3. Physicochemical Properties and ADME Analysis

The physicochemical properties and BBB permeability of our designed and synthesized compounds were predicted using the Swiss ADME online tool (http://www.swissadme.ch/, accessed on 30 March 2022) and Light BBB (http://bioanalysis.cau.ac.kr:7030/, accessed on 30 March 2022), respectively. The structures of compounds were drawn using ChemDraw Ultra 12.0 software.

### 3.4. Statistical Analysis

Statistical analysis was performed using Prism 4.0 (Graph Pad Software, San Diego, CA, USA). Non-linear regression analysis was used in the competitive binding studies. Log IC_50_ values were calculated by fitting the data from the competition studies to a one-site competition model.

## 4. Conclusions

Based on SAR studies of our previously published work, a series of 2,3-dihydrothiazolo[4,5-d]pyrimidine derivatives were synthesized. Some representative compounds were evaluated for their binding affinity to the CRF_1_ receptors as a part of our ongoing research to develop CRF_1_ receptor antagonists with superior binding affinity. These compounds were designed to have a basic pharmacophore for optimum binding affinity to CRF_1_ receptors, including a substituted phenyl at position N-3, a methyl group at position C-5, and a dialkylamino group at position C-7. The binding affinity of newly synthesized compounds was evaluated in two different experiments. The compounds were screened in a primary assay by measuring their ability to inhibit the specific binding of [^125^I]-Tyr^0^ sauvagine to membranes from HEK 293 cells stably expressing the CRF_1_ receptors. A secondary assay was performed by determining the competitive binding affinity of select compounds in comparison to the standard drug, antalarmin. Nine compounds (compounds **2**, **5**, **10**, **14**, and **19**–**23**) from the synthesized compounds have shown their ability to inhibit more than 50% of [^125^I]-Tyr^0^ sauvagine binding. These nine compounds were able to inhibit [^125^I]-Tyr^0^ sauvagine binding to CRF_1_ receptors in a dose-dependent manner, with Log IC_50_ ± SE values of −8.22 ± 0.33, −7.95 ± 0.26, −7.51 ± 0.15, −6.84 ± 0.09, −7.39 ± 0.34, −8.04 ± 0.16, −7.88 ± 0.09, −7.04 ± 0.24, and −6.95 ± 0.12 for the compounds **2**, **5**, **10**, **14**, **19**, **20**, **21**, **22**, and **23**, respectively, compared to the Log IC_50_ ± SE value of −7.78 ± 0.21 for the standard drug antalarmin.

Four compounds (compounds **2**, **5**, **20**, **21**) from the synthesized compounds have shown better inhibitory effects than antalarmin. The best compound (compound **2**) is 2.5 times more potent than antalarmin in terms of IC_50,_ which is a great achievement. It can be argued that compound **2** may be one of the top small-molecule non-peptide CRF_1_R antagonists (in terms of IC_50_) ever developed. In silico prediction of physicochemical properties showed that all of our best lead compounds (compounds **2**, **5**, **19**, **20**) are predicted to have better drug-like properties (no violation of Lipinski’s rule) than the standard drug antalarmin (one violation of Lipinski’s rule), and these compounds are also predicted to have the ability to cross BBB. It can be concluded that this study resulted in the development of four compounds with excellent binding affinity and inhibitory effects and better druggable properties. This excellent binding affinity is not only compared to our previous compounds [53,54], but also compared to any other compound developed in the last 40 years of the history of small molecule CRF_1_R antagonists. Thus, further research in this direction may lead to the development of compounds that can be drug candidates for stress-related disorders and congenital adrenal hyperplasia.

## Figures and Tables

**Table 1:** List of substitutions of the final target compounds (**1**–**23**).

No	R_1_	R_2_	X/Y
**1**	C_2_H_5_	C_4_H_9_	2,4-di Cl
**2**	C_3_H_7_	C_3_H_7_	2,4-di Cl
**3**	C_2_H_4_-O-CH_3_	C_2_H_4_-O-CH_3_	2,4-di Cl
**4**	C_5_H_11_	C_5_H_11_	2,4-di Cl
**5**	c-propylmethyl	C_3_H_7_	2,4-di Cl
**6**	C_2_H_5_-CH-C_2_H_5_	H	2,4-di Cl
**7**	C_3_H_7_-CH-C_3_H_7_	H	2,4-di Cl
**8**	C_3_H_7_	C_3_H_7_	2-Br-4-CH(CH_3_)_2_
**9**	c-propylmethyl	C_3_H_7_	2-Br-4-CH(CH_3_)_2_
**10**	C_2_H_4_-O-CH_3_	C_2_H_4_-O-CH_3_	2-Br-4-CH(CH_3_)_2_
**11**	C_5_H_11_	C_5_H_11_	2-Br-4-CH(CH_3_)_2_
**12**	C_2_H_5_-CH-C_2_H_5_	H	2-Br-4-CH(CH_3_)_2_
**13**	C_3_H_7_-CH-C_3_H_7_	H	2-Br-4-CH(CH_3_)_2_
**14**	C_2_H_5_	C_4_H_9_	2,4-di CH_3_
**15**	C_3_H_7_	C_3_H_7_	2,4-diCH_3_
**16**	C_2_H_4_-O-CH_3_	C_2_H_4_-O-CH_3_	2,4-di CH_3_
**17**	c-propylmethyl	C_3_H_7_	2,4-di CH_3_
**18**	C_2_H_5_-CH-C_2_H_5_	H	2,4-di CH_3_
**19**	C_3_H_7_-CH-C_3_H_7_	H	2,4-di CH_3_
**20**	C_2_H_5_	C_4_H_9_	2-CH_3_-4-OCH_3_
**21**	C_3_H_7_	C_3_H_7_	2-CH_3_-4-OCH_3_
**22**	c-propylmethyl	C_3_H_7_	2-CH_3_-4-OCH_3_
**23**	C_2_H_5_-CH-C_2_H_5_	H	2-CH_3_-4-OCH_3_

**Table 2:** IC_50_ (nM) values of the lead compounds in comparison to antalarmin.

Comp	Anta	2	5	10	14	19	20	21	22	23
IC_50_ (nM)	16.63	6.10	11.12	31.05	144.2	41.11	9.16	13.12	91.62	111.4

**Table 3:** Physicochemical parameters and BBB permeability of the best five compounds.

Comp	MW	RB	HBA	HBD	cLogP	Vio LR	BBB Permeability
**2**	424.39	6	3	0	3.72	0	Permeable
**5**	436.40	6	3	0	3.94	0	Permeable
**19**	397.58	7	3	1	3.41	0	Permeable
**20**	399.55	7	4	0	2.64	0	permeable
**21**	399.55	7	4	0	2.64	0	Permeable
Antalarmin	378.55	6	2	0	4.33	1	Permeable

## Data Availability

Data are contained within the article and Supplementary Materials.

## Supplementary Materials

The following supporting information can be downloaded at: https://www.mdpi.com/article/10.3390/molecules29153647/s1, Compounds characterization: ^1^H NMR, ^13^C NMR, and HRMS spectrum of all the target compounds.
