# Supplementary material for: Design, Synthesis, and Biological Evaluations of Novel Thiazolo[4,5-d]pyrimidine Corticotropin Releasing Factor (CRF) Receptor Antagonists as Potential Treatments for Stress Related Disorders and Congenital Adrenal Hyperplasia (CAH)
Source: Molecules. 2024 Aug 1;29(15):3647. doi: 10.3390/molecules29153647 (PMC11314199; doi:10.3390/molecules29153647)
Supplement: Supplementary file 1 [file molecules-29-03647-s001.zip › molecules-3092761-supplementary.pdf]

Rabiul-5311
PROTON

Compound 1_proton NMR

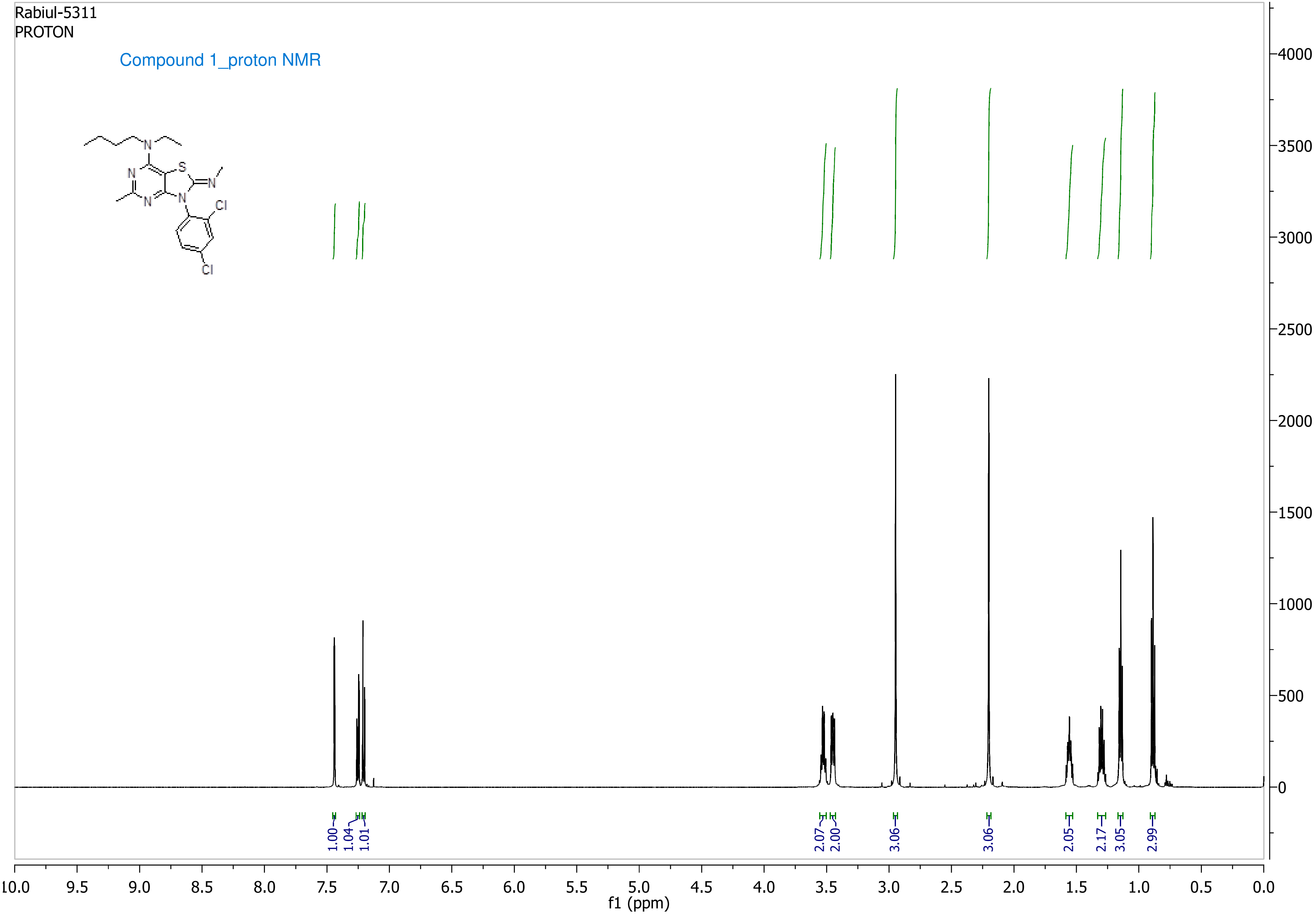

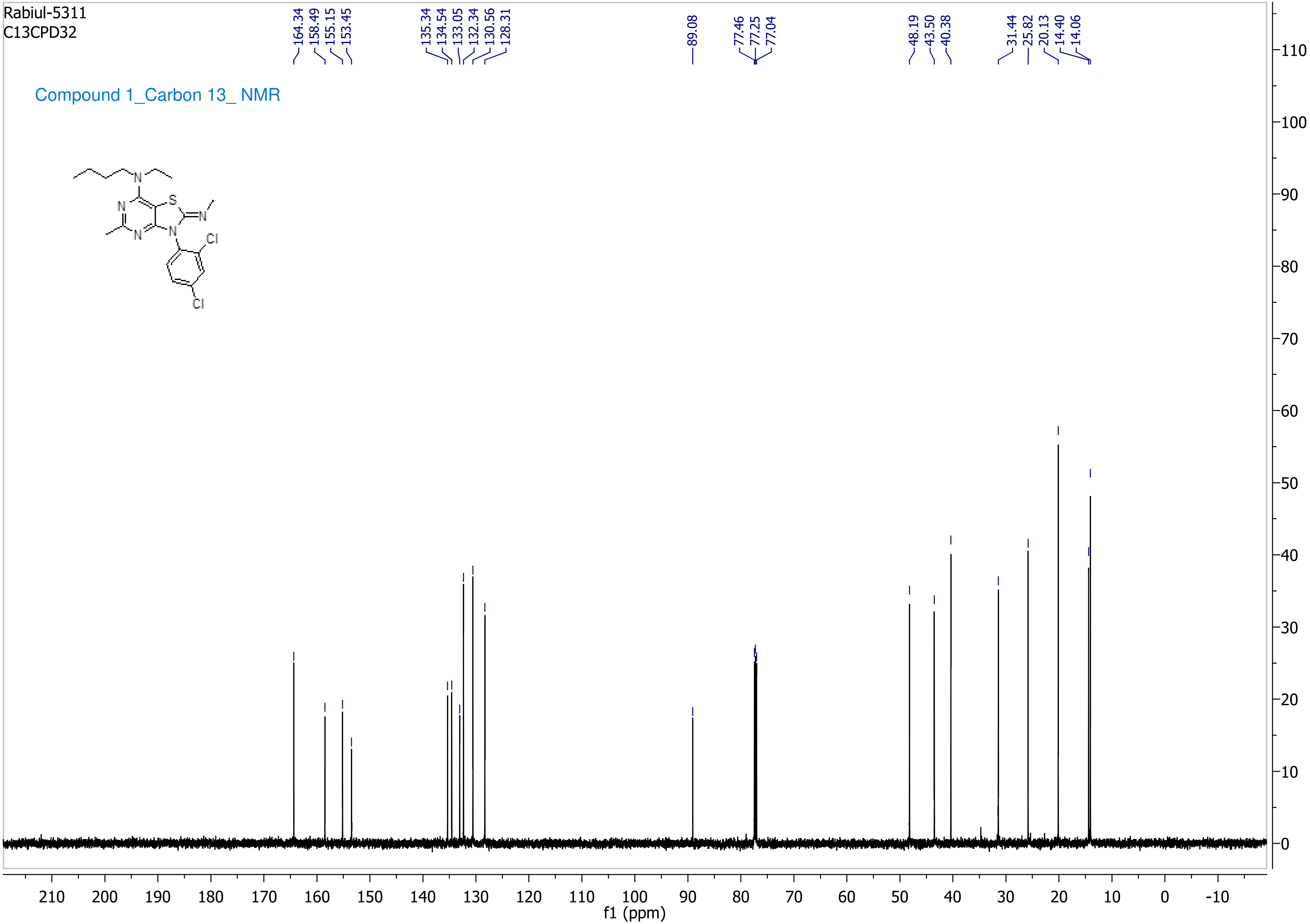

Rabiul-5311
C13CPD32
Compound 1_Carbon 13_ NMR
164.34
158.49
155.15
153.45
135.34
134.54
133.05
132.34
130.56
128.31
89.08
77.46
77.25
77.04
48.19
43.50
40.38
31.44
25.82
20.13
14.40
14.06
N
S
N
N
N
Cl
Cl
f1 (ppm)

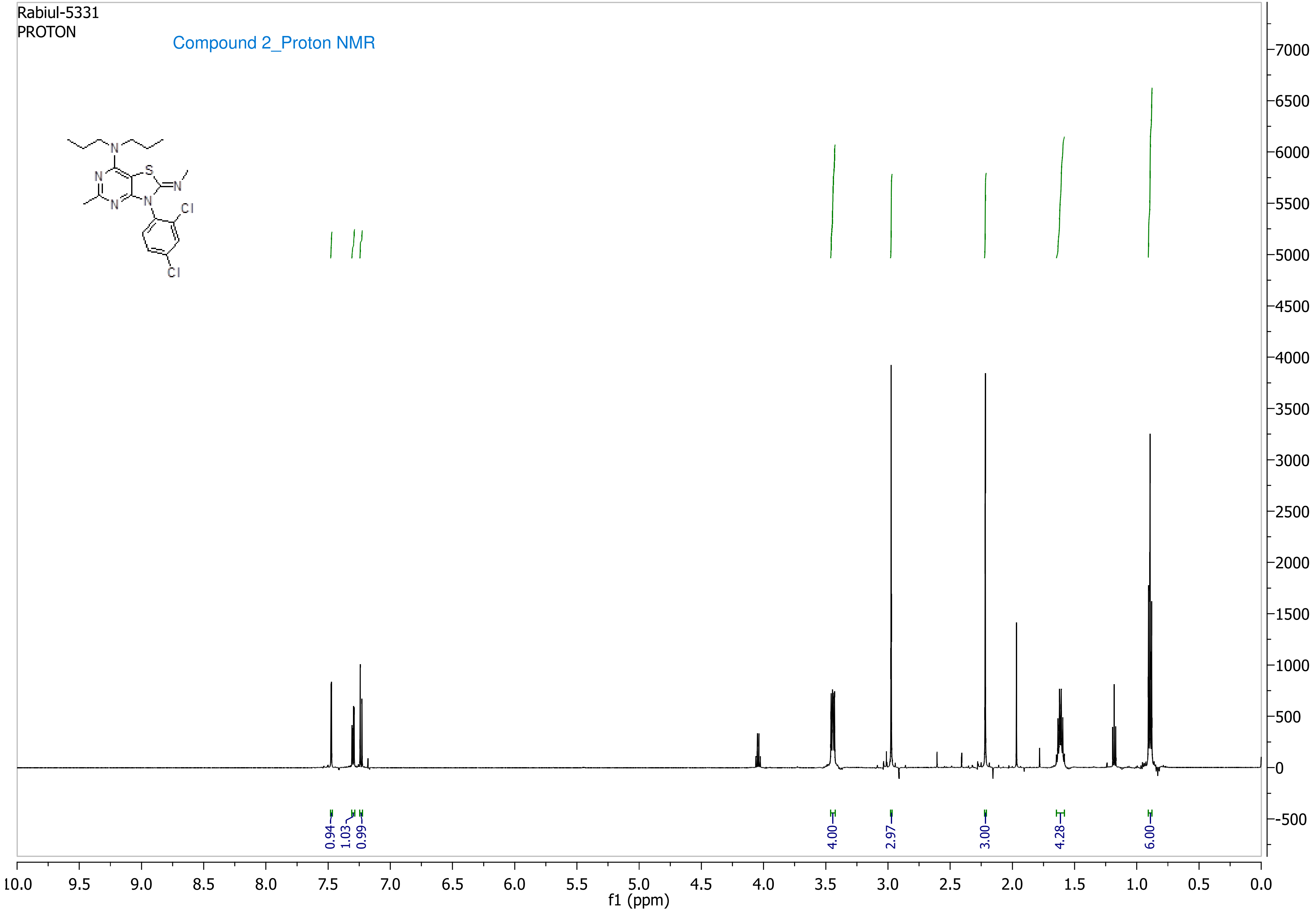
Rabiul-5331
PROTON
Compound 2_Proton NMR
0.94
1.03
0.99
4.00
2.97
3.00
4.28
6.00
f1 (ppm)

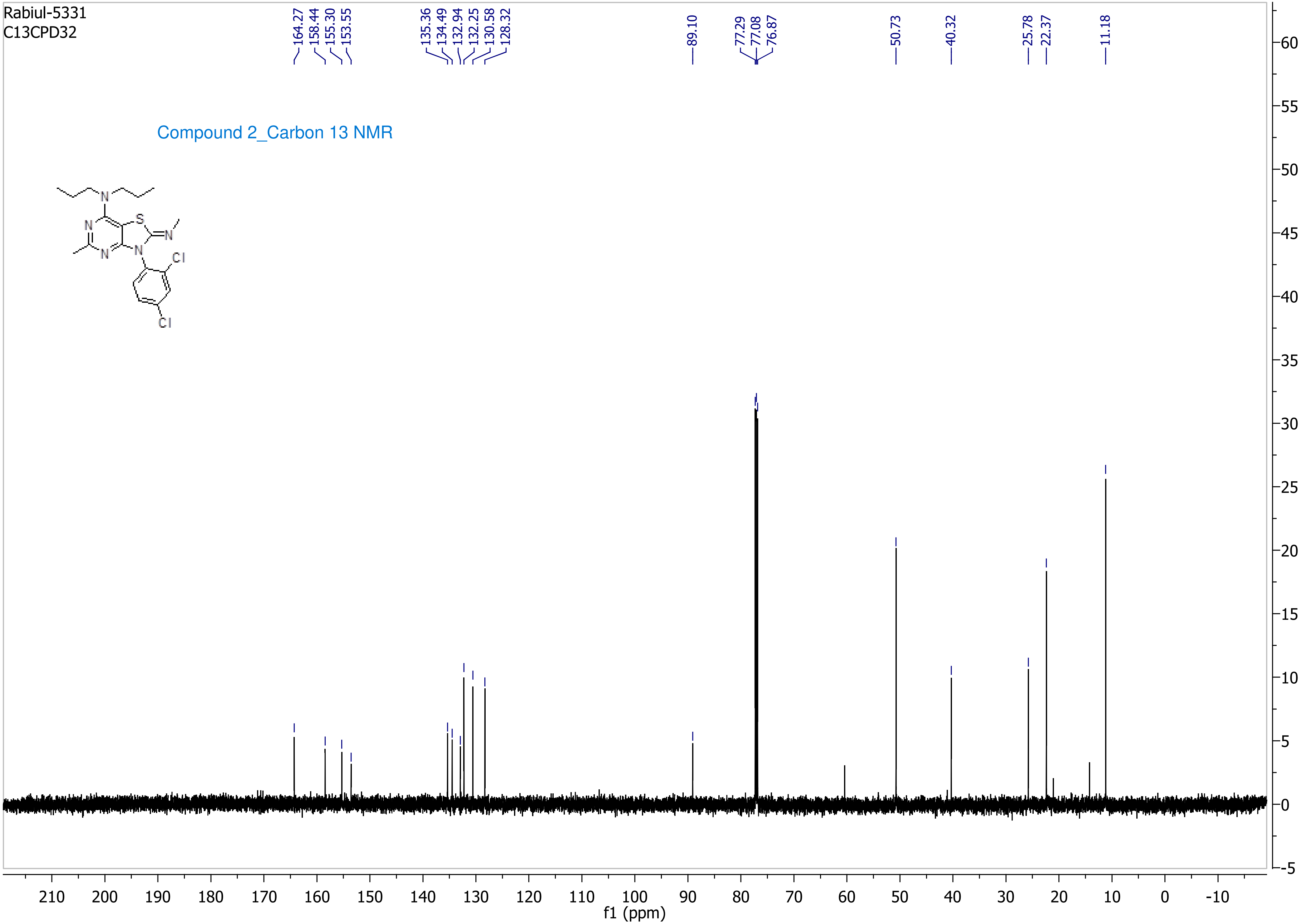
Rabiul-5331
C13CPD32
164.27
158.44
155.30
153.55
135.36
134.49
132.94
132.25
130.58
128.32
89.10
77.29
77.08
76.87
50.73
40.32
25.78
22.37
11.18
Compound 2_Carbon 13 NMR
N
S
N
N
N
Cl
Cl
f1 (ppm)

UB Mass Spectrometry Facility - SmartFormula Report

Analysis Info

Analysis Name	D:\Data\IC_3-21-19\5331_000001.d	Acquisition Date	3/21/2019 5:32:27 PM
Method	Bruker_11052015	Operator	
Sample Name	5331	Instrument	solariX
Comment			

Acquisition Parameters

Acquisition Mode	Single MS	Nebulizer gas flow rate	1.2 bar
Polarity	Positive	Drying Gas Temperature	180.0 °C
Broadband Low Mass	147.4 m/z	Drying Gas Flow Rate	4.2 L/min
Broadband High Mass	1500.0 m/z	Calibration Date	Thu Sep 27 10:56:48 2018
Time of Flight to Detector	0.001 sec	Data Acquisition Size	1048576
Ion Accumulation Time	0.100 sec	Data Processing Size (SI)	2097152
Acquired Scans	20	Apodization	Full-Sine
Capillary	6000.0 V		

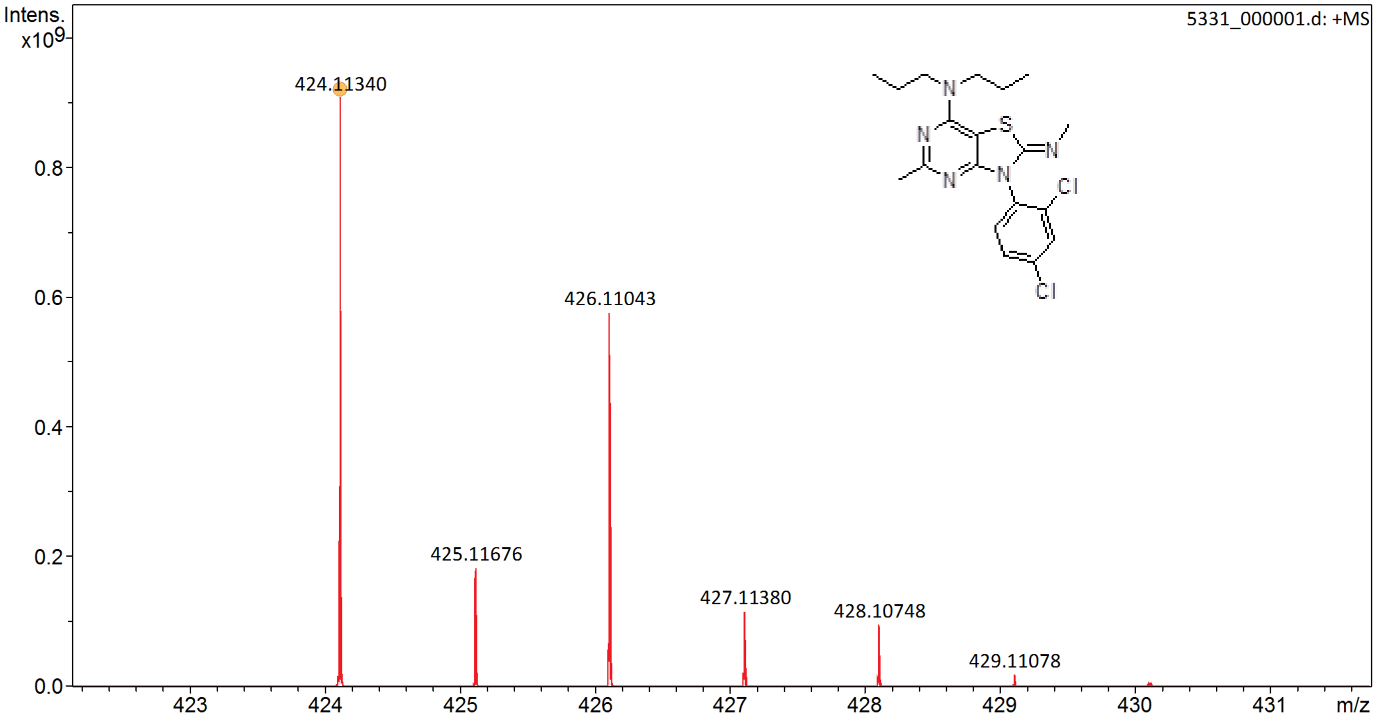

Adduct	Meas. m/z	#	Ion Formula	Score	m/z	err [ppm]	Mean err [ppm]	mSigma	rdb	e⁻ Conf	N-Rule
M+H	424.113399	1	C19H24Cl2N5S	100.00	424.112399	-2.4	-2.5	26.0	9.5	even	ok
M+Na	446.095180	1	C19H23Cl2N5NaS	100.00	446.094343	-1.9	-1.9	15.7	9.5	even	ok

Rabiul-5341
PROTON

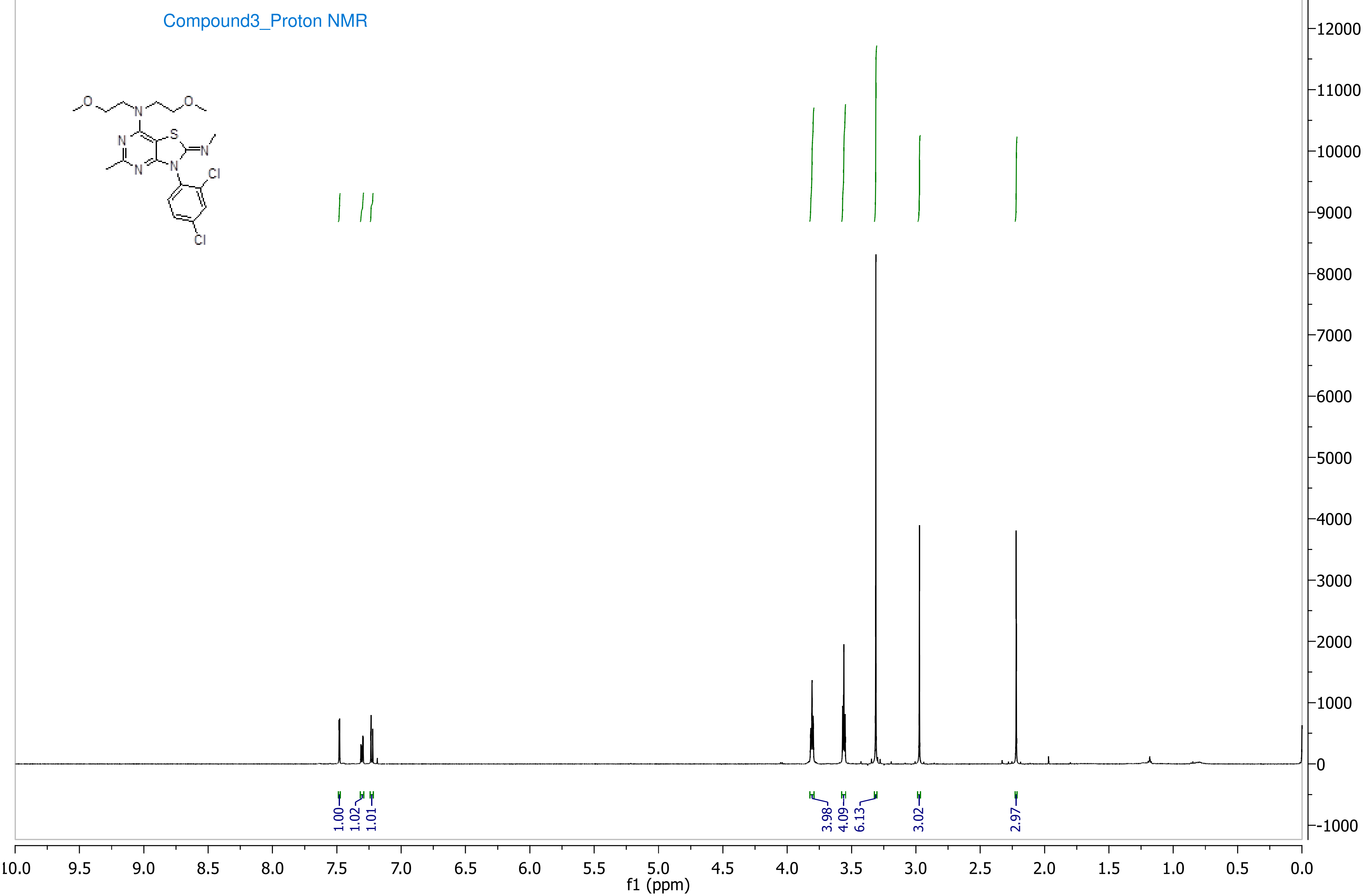

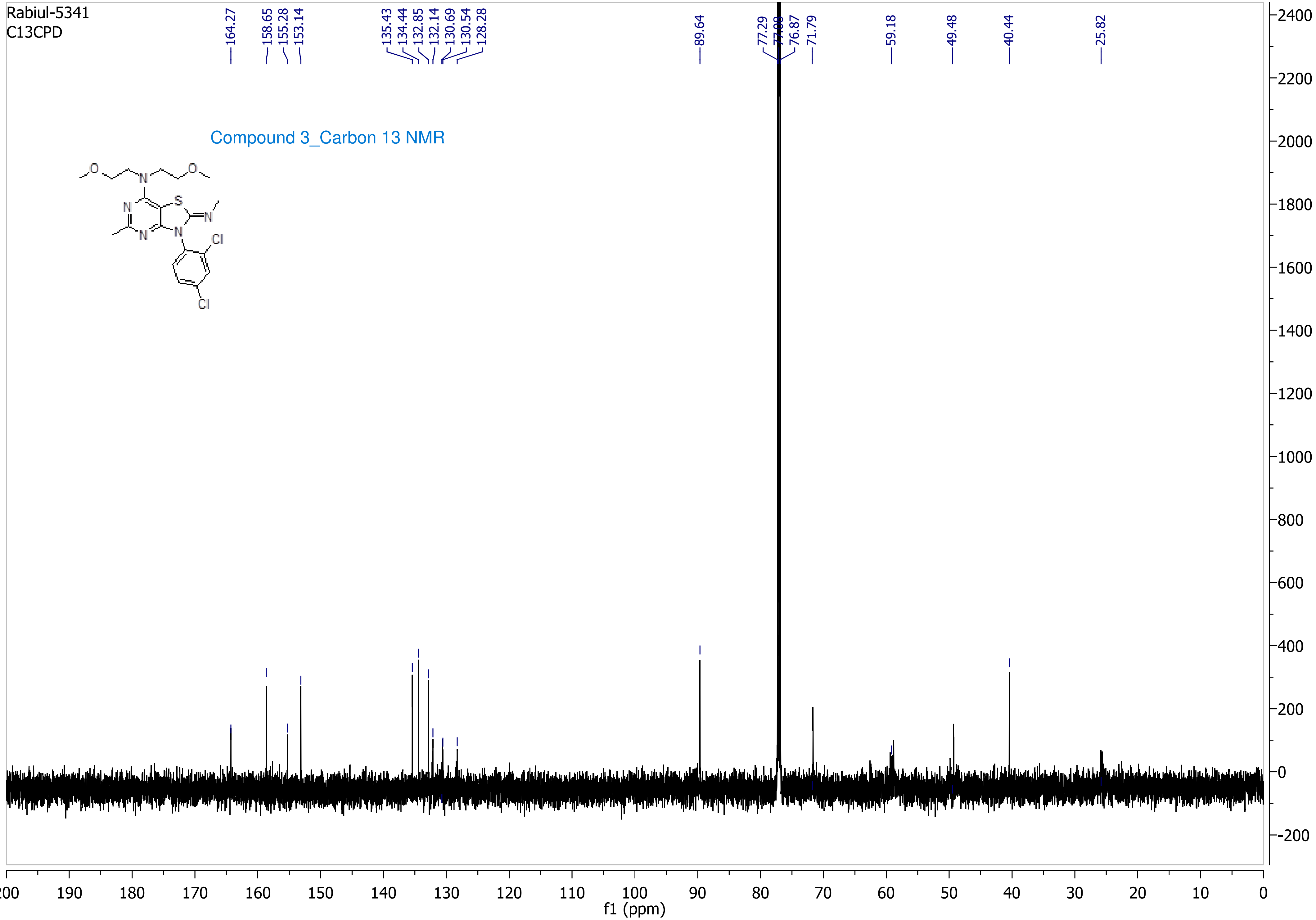

Rabiul-5341
C13CPD
164.27
158.65
155.28
153.14
135.43
134.44
132.85
132.14
130.69
130.54
128.28
89.64
77.29
76.87
71.79
59.18
49.48
40.44
25.82
Compound 3_Carbon 13 NMR
N
O
S
Cl
f1 (ppm)

Compound 3_MS

UB Mass Spectrometry Facility - SmartFormula Report

Analysis Info

Analysis Name	D:\Data\IC_3-21-19\5341_000001.d	Acquisition Date	3/21/2019 5:47:59 PM
Method	Bruker_11052015	Operator	
Sample Name	5341	Instrument	solariX
Comment			

Acquisition Parameters

Acquisition Mode	Single MS	Nebulizer gas flow rate	1.2 bar
Polarity	Positive	Drying Gas Temperature	180.0 °C
Broadband Low Mass	147.4 m/z	Drying Gas Flow Rate	4.2 L/min
Broadband High Mass	1500.0 m/z	Calibration Date	Thu Sep 27 10:56:48 2018
Time of Flight to Detector	0.001 sec	Data Acquisition Size	1048576
Ion Accumulation Time	0.100 sec	Data Processing Size (SI)	2097152
Acquired Scans	20	Apodization	Full-Sine
Capillary	6000.0 V		

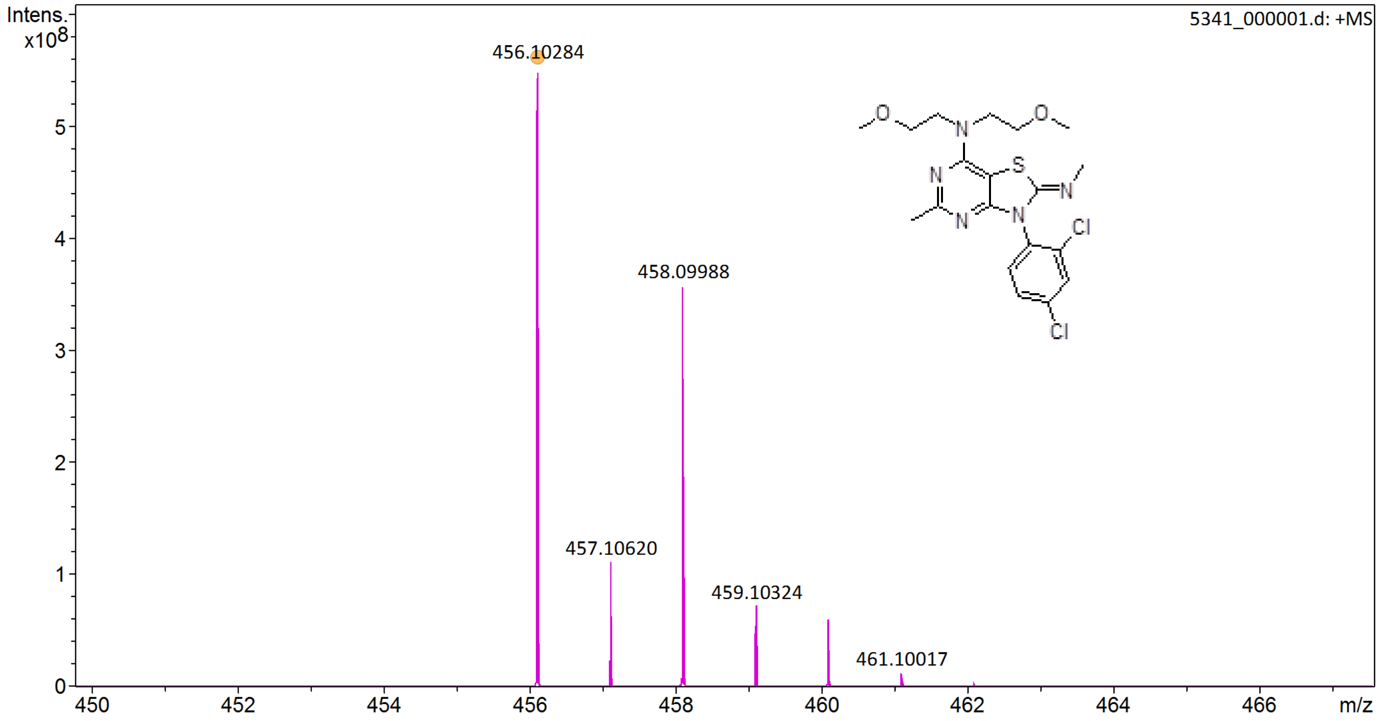

Adduct	Meas. m/z	#	Ion Formula	Score	m/z	err [ppm]	Mean err [ppm]	mSigma	rdb	e⁻ Conf	N-Rule
M+H	456.102845	1	C19H24Cl2N5O2S	100.00	456.102228	-1.4	-1.4	22.0	9.5	even	ok

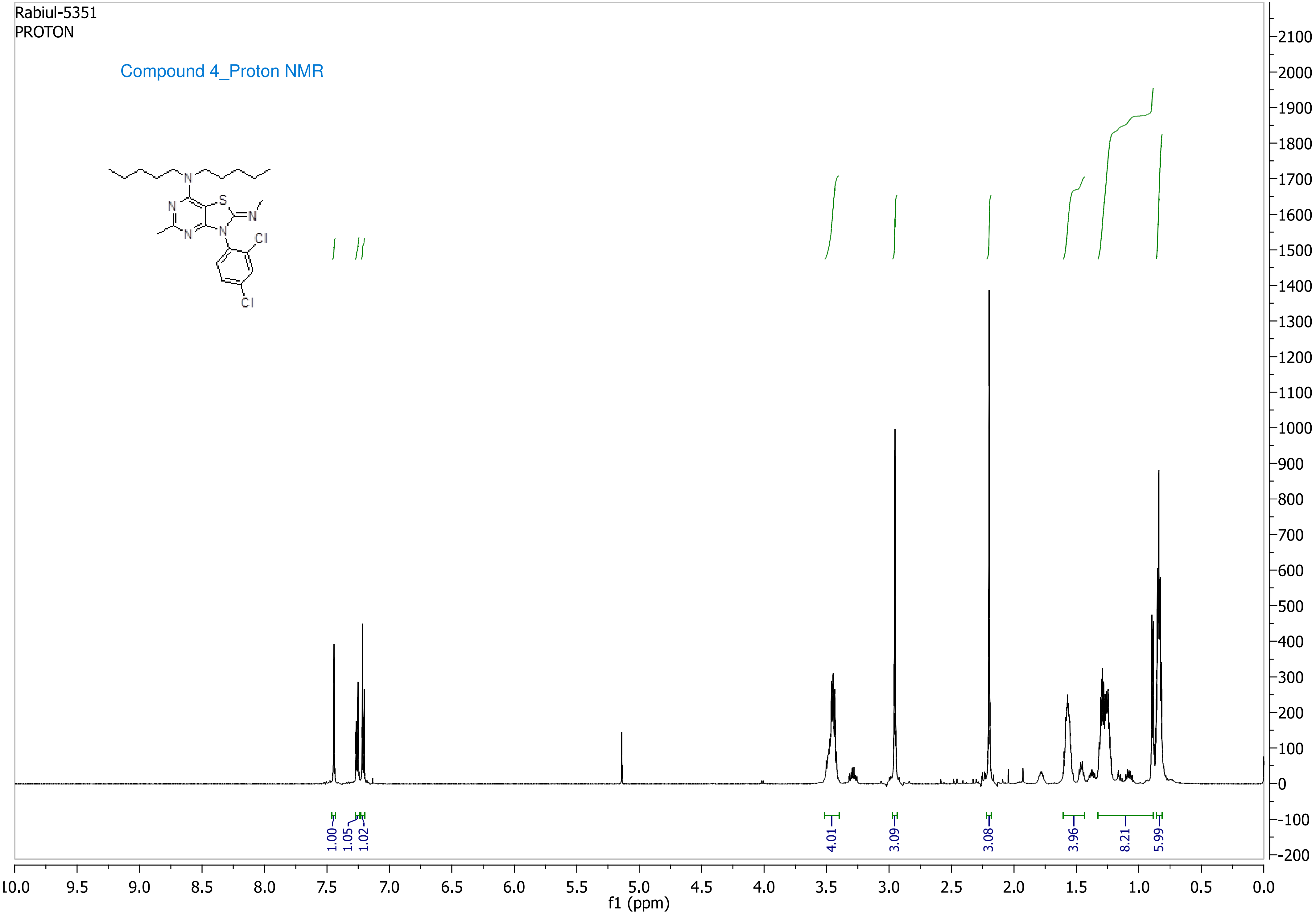
Rabiul-5351
PROTON
Compound 4_Proton NMR
N
S
N
N
N
N
Cl
Cl
1.00
1.05
1.02
4.01
3.09
3.08
3.96
8.21
5.99
f1 (ppm)

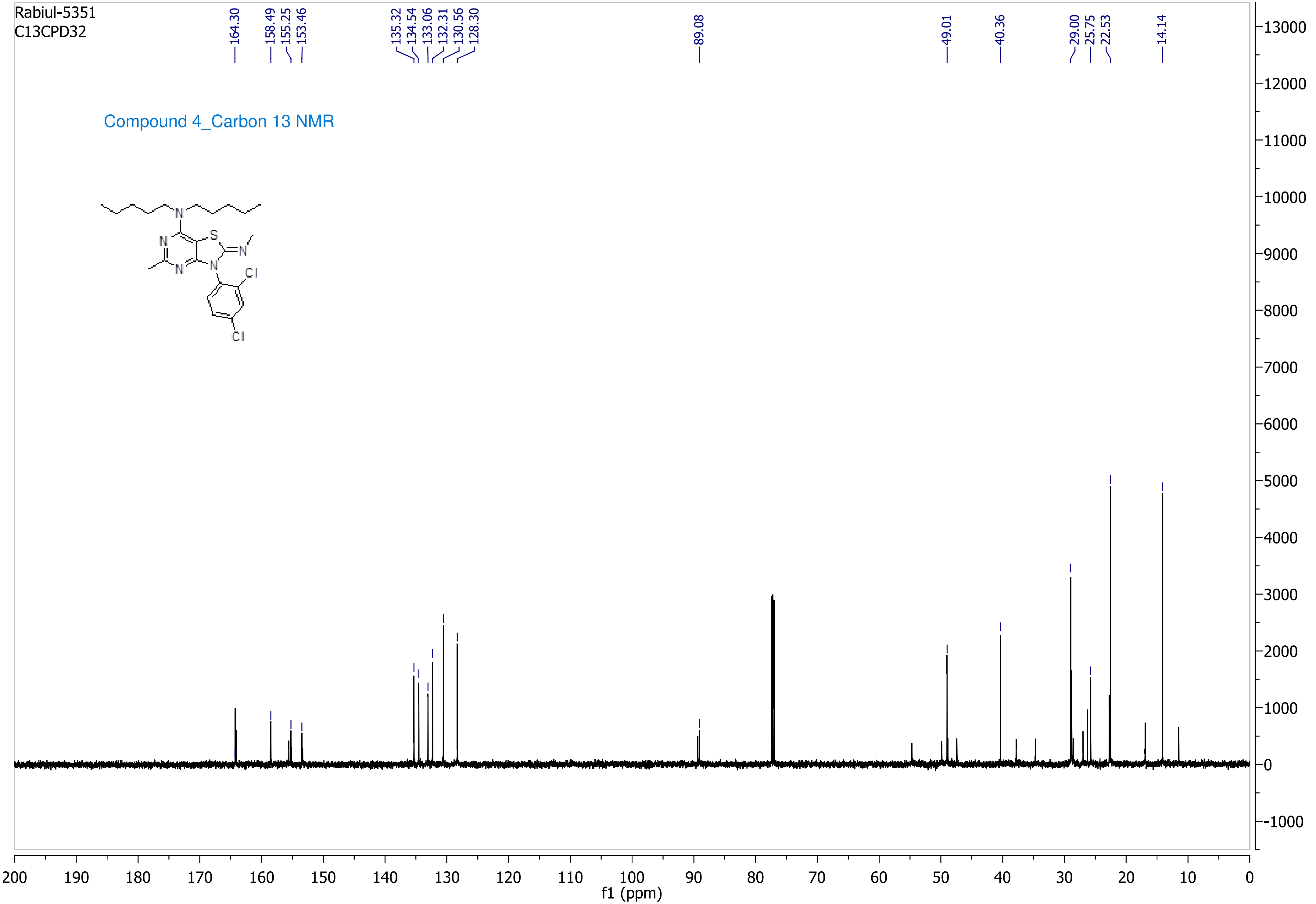

Rabiul-5351
C13CPD32
Compound 4_Carbon 13 NMR
164.30
158.49
155.25
153.46
135.32
134.54
133.06
132.31
130.56
128.30
89.08
49.01
40.36
29.00
25.75
22.53
14.14
N
S
N
N
N
Cl
Cl
13000
12000
11000
10000
9000
8000
7000
6000
5000
4000
3000
2000
1000
0
-1000
200 190 180 170 160 150 140 130 120 110 100 90 80 70 60 50 40 30 20 10 0
f1 (ppm)

Compound 4_MS

UB Mass Spectrometry Facility - SmartFormula Report

Analysis Info

Analysis Name	D:\Data\IC_3-21-19\5351_000001.d	Acquisition Date	3/21/2019 5:51:19 PM
Method	Bruker_11052015	Operator	
Sample Name	5351	Instrument	solariX
Comment			

Acquisition Parameters

Acquisition Mode	Single MS	Nebulizer gas flow rate	1.2 bar
Polarity	Positive	Drying Gas Temperature	180.0 °C
Broadband Low Mass	147.4 m/z	Drying Gas Flow Rate	4.2 L/min
Broadband High Mass	1500.0 m/z	Calibration Date	Thu Sep 27 10:56:48 2018
Time of Flight to Detector	0.001 sec	Data Acquisition Size	1048576
Ion Accumulation Time	0.100 sec	Data Processing Size (SI)	2097152
Acquired Scans	20	Apodization	Full-Sine
Capillary	6000.0 V		

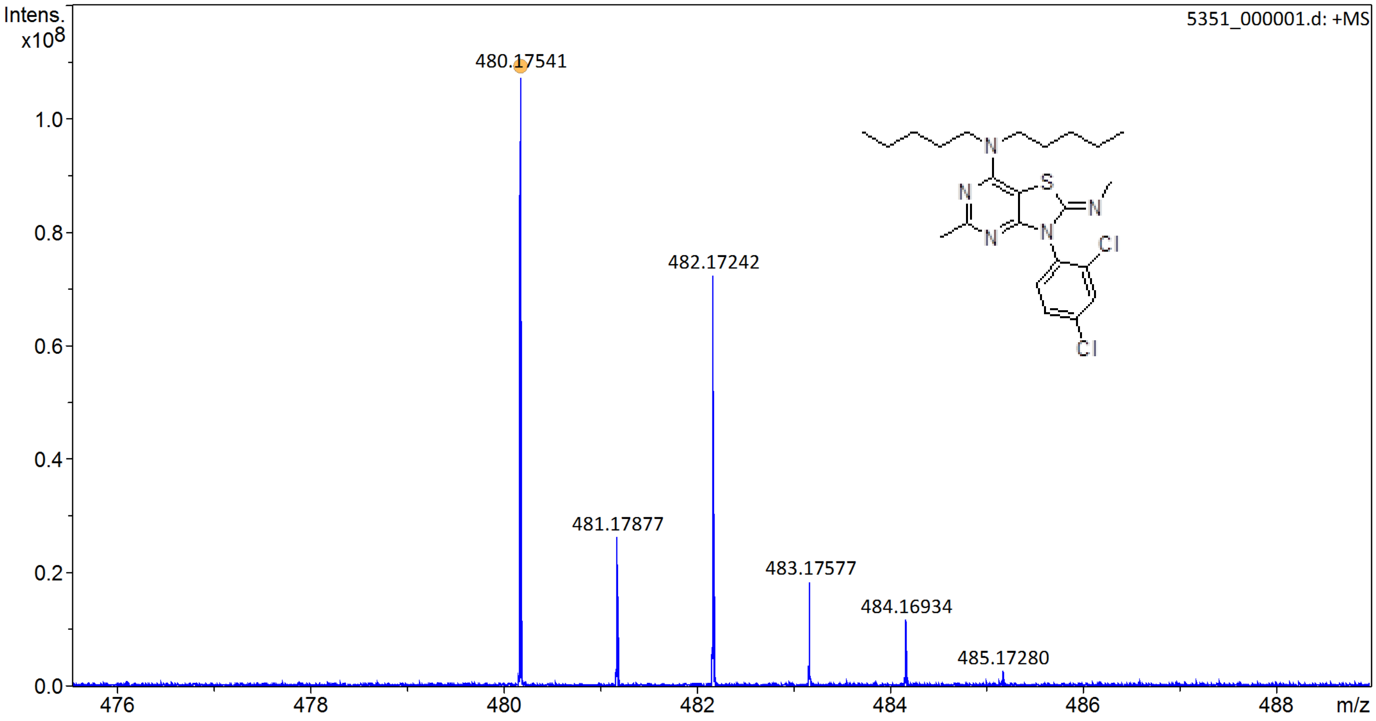

Adduct	Meas. m/z	#	Ion Formula	Score	m/z	err [ppm]	Mean err [ppm]	mSigma	rdb	e¯ Conf	N-Rule
M+H	480.175406	1	C23H32Cl2N5S	100.00	480.174999	-0.8	-0.9	14.1	9.5	even	ok
M+Na	502.157142	1	C23H31Cl2N5NaS	100.00	502.156943	-0.4	-0.4	23.7	9.5	even	ok

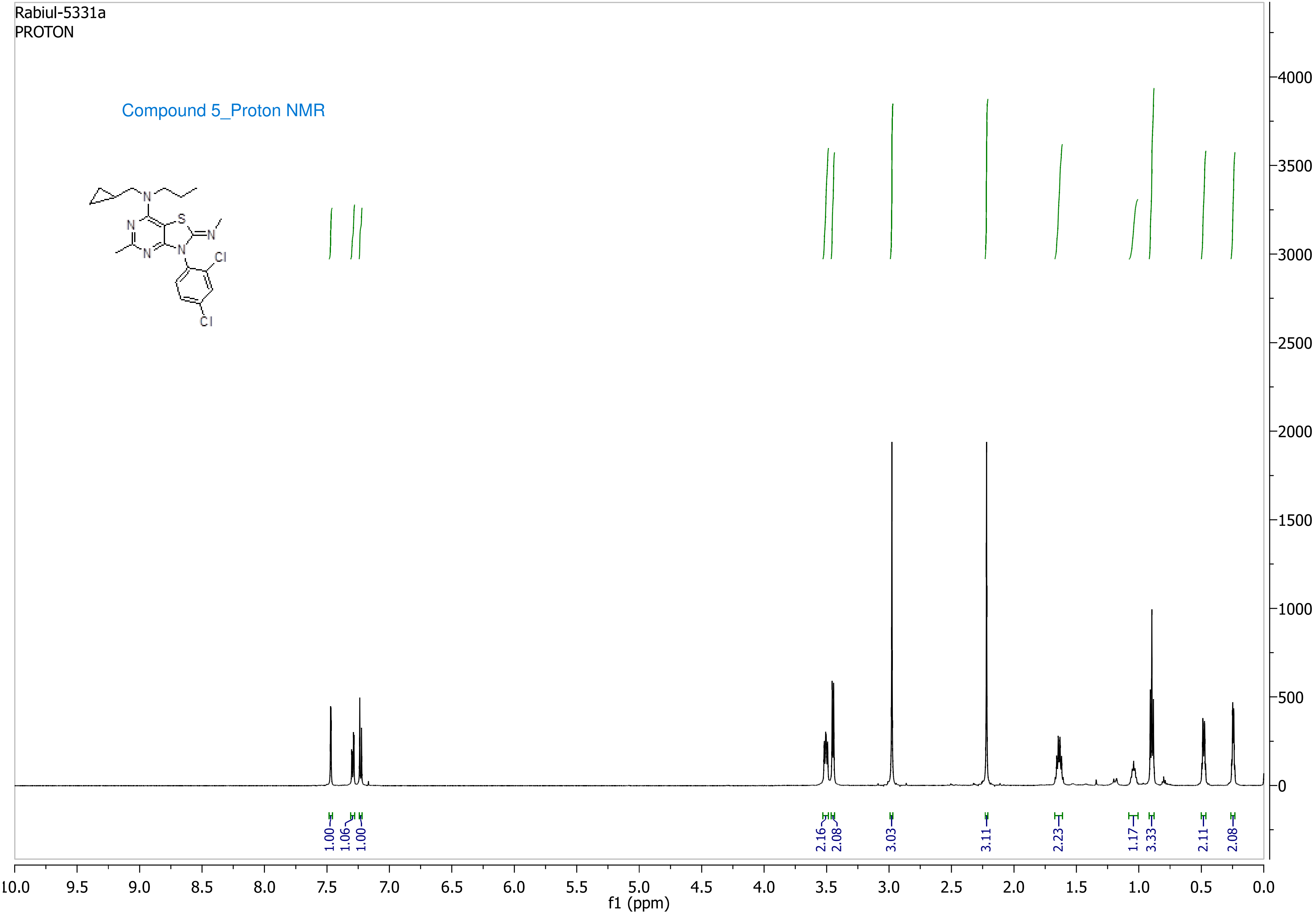
Rabiul-5331a
PROTON
Compound 5_Proton NMR
N
S
N
N
N
N
Cl
Cl
1.00
1.06
1.00
2.16
2.08
3.03
3.11
2.23
1.17
3.33
2.11
2.08
10.0 9.5 9.0 8.5 8.0 7.5 7.0 6.5 6.0 5.5 5.0 4.5 4.0 3.5 3.0 2.5 2.0 1.5 1.0 0.5 0.0
f1 (ppm)
4000
3500
3000
2500
2000
1500
1000
500
0

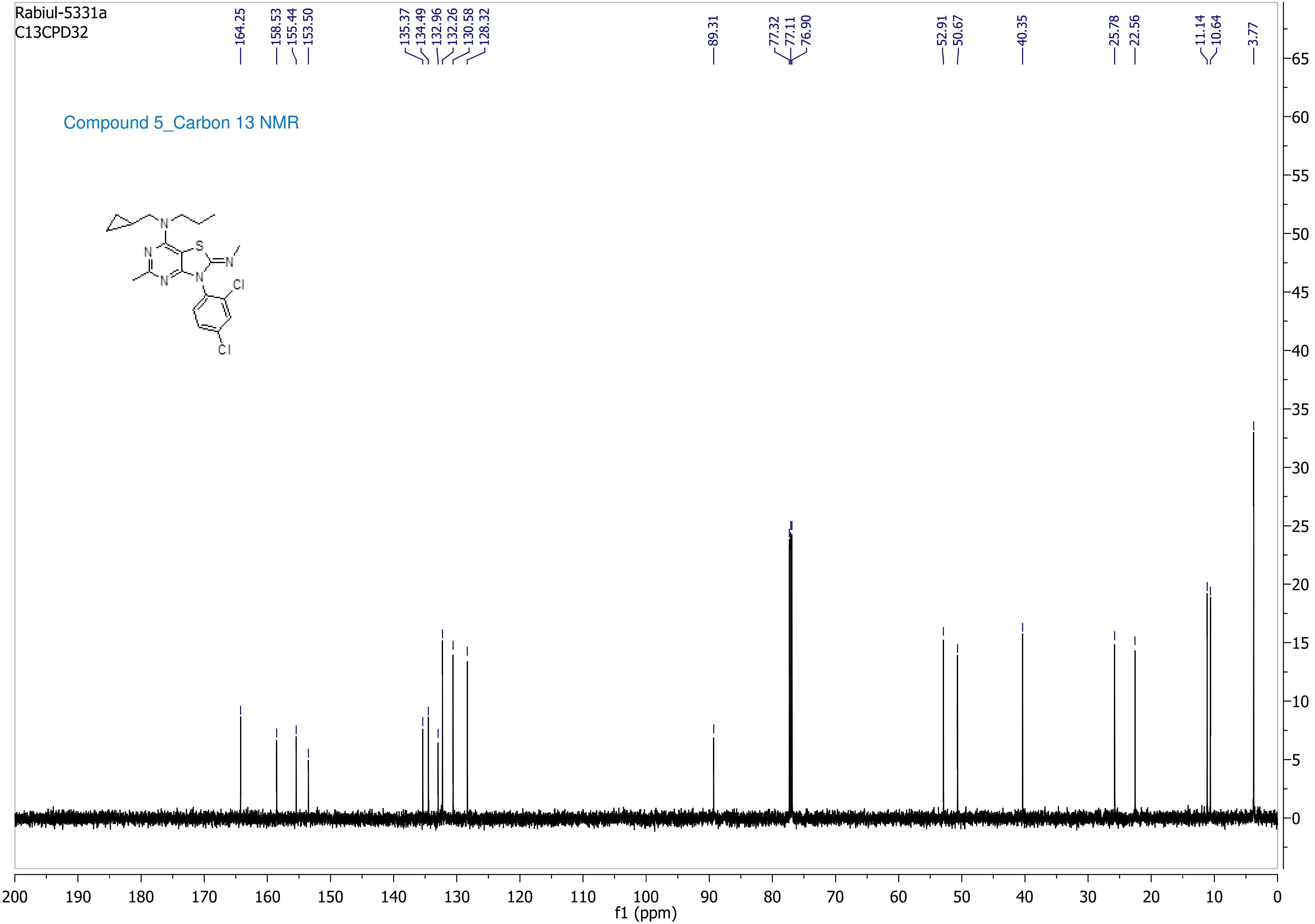

Rabiul-5331a
C13CPD32
Compound 5_Carbon 13 NMR
164.25
158.53
155.44
153.50
135.37
134.49
132.96
132.26
130.58
128.32
89.31
77.32
77.11
76.90
52.91
50.67
40.35
25.78
22.56
11.14
10.64
3.77
N
S
N
N
N
N
Cl
Cl
f1 (ppm)

Compound 5_MS

UB Mass Spectrometry Facility - SmartFormula Report

Analysis Info

Analysis Name	D:\Data\IC_3-21-19\5331a_000001.d	Acquisition Date	3/21/2019 7:13:29 PM
Method	Bruker_11052015	Operator	
Sample Name	5331a	Instrument	solariX
Comment			

Acquisition Parameters

Acquisition Mode	Single MS	Nebulizer gas flow rate	1.2 bar
Polarity	Positive	Drying Gas Temperature	180.0 °C
Broadband Low Mass	147.4 m/z	Drying Gas Flow Rate	4.2 L/min
Broadband High Mass	1500.0 m/z	Calibration Date	Thu Sep 27 10:56:48 2018
Time of Flight to Detector	0.001 sec	Data Acquisition Size	1048576
Ion Accumulation Time	0.100 sec	Data Processing Size (SI)	2097152
Acquired Scans	20	Apodization	Full-Sine
Capillary	6000.0 V		

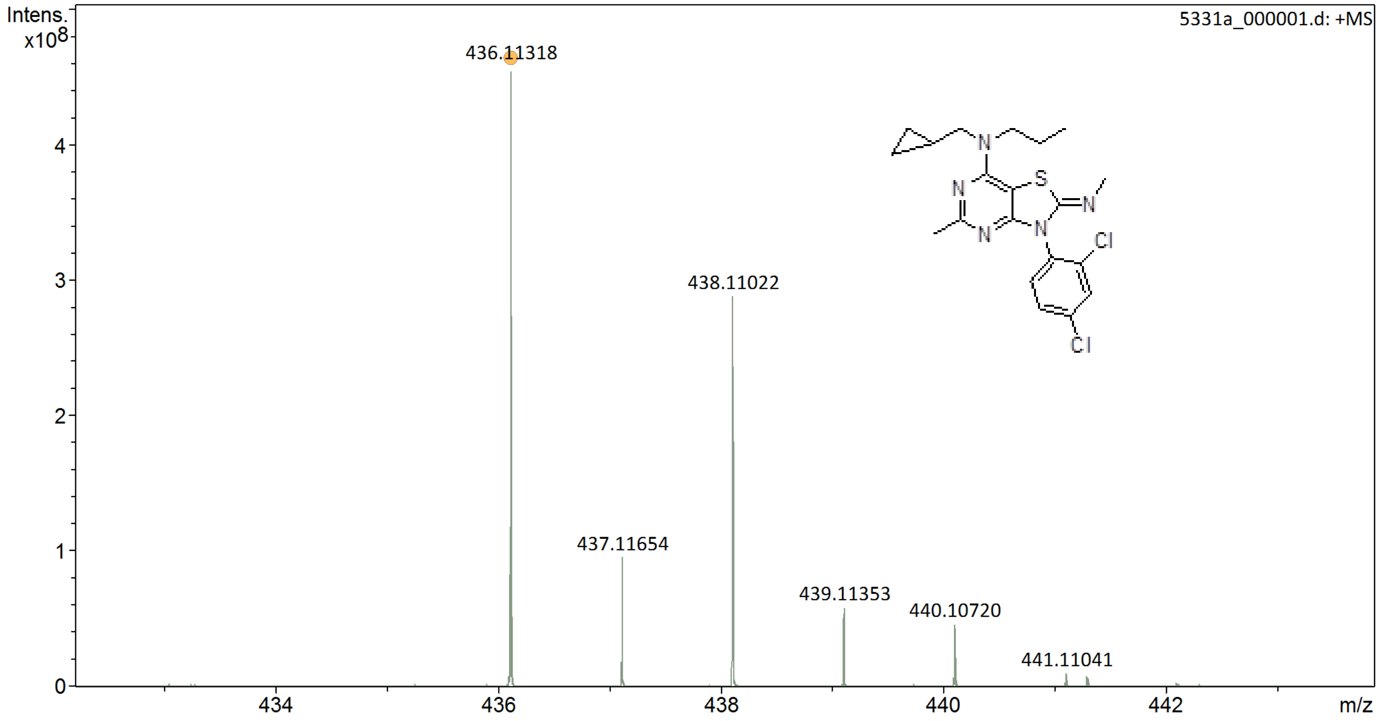

Adduct	Meas. m/z	#	Ion Formula	Score	m/z	err [ppm]	Mean err [ppm]	mSigma	rdb	e⁻ Conf	N-Rule
M+H	436.113182	1	C20H24Cl2N5S	100.00	436.112399	-1.8	-1.9	30.4	10.5	even	ok
M+Na	458.094930	1	C20H23Cl2N5NaS	100.00	458.094343	-1.3	-1.4	18.5	10.5	even	ok

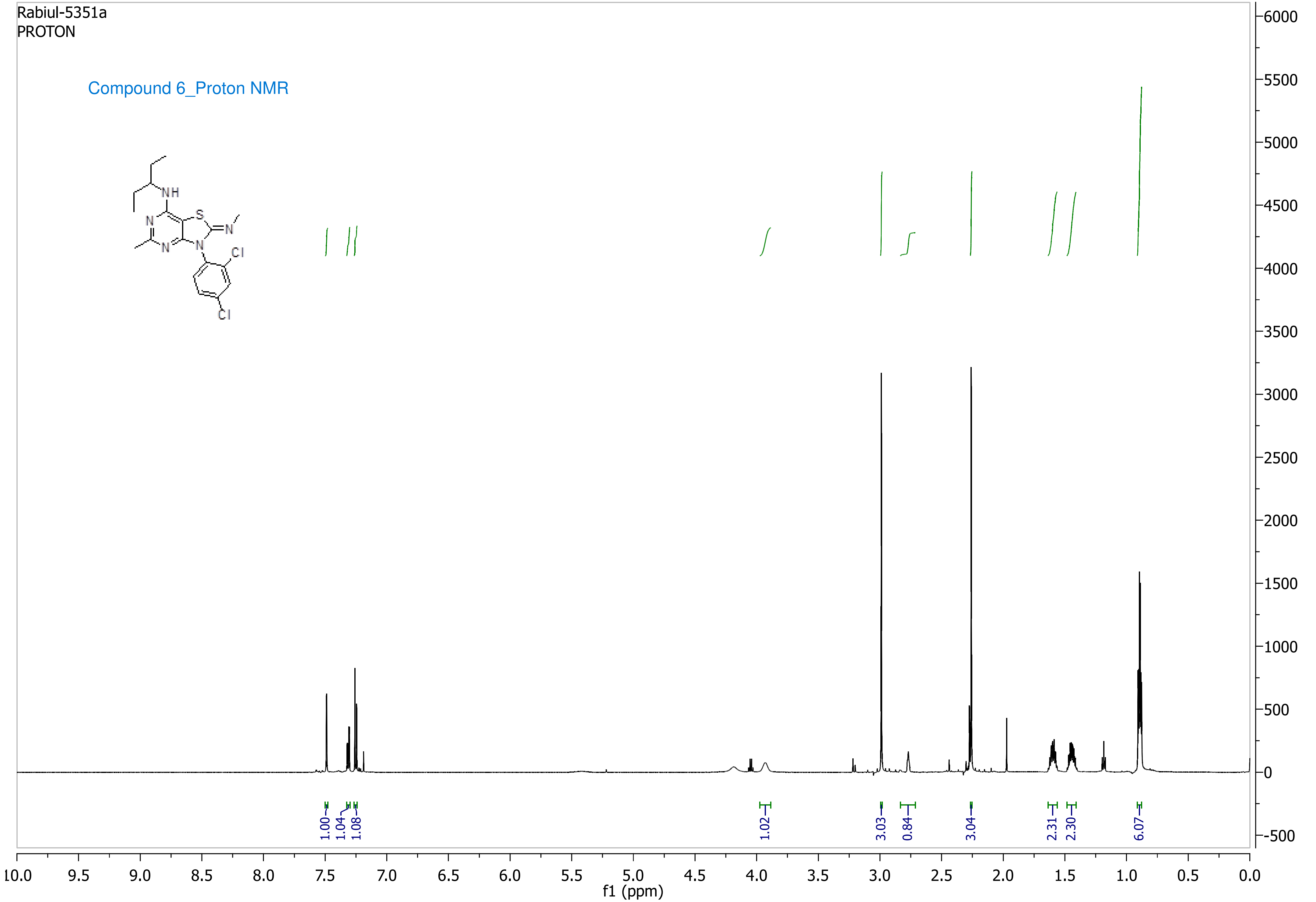

Rabiul-5351a
PROTON
Compound 6_Proton NMR
NH
S
N
N
N
N
Cl
Cl
1.00
1.04
1.08
1.02
3.03
0.84
3.04
2.31
2.30
6.07
f1 (ppm)

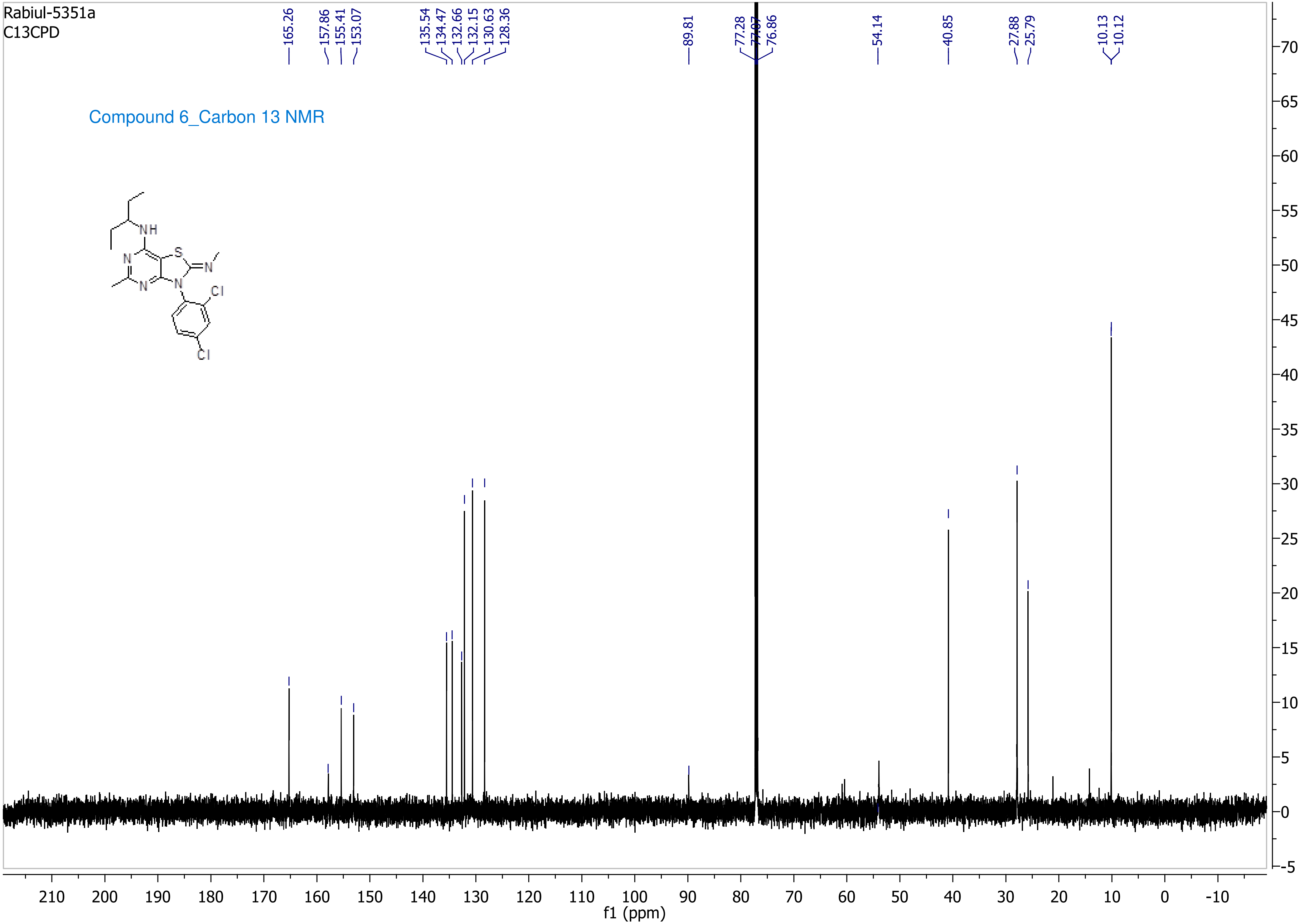

Rabiul-5351a
C13CPD
165.26
157.86
155.41
153.07
135.54
134.47
132.66
132.15
130.63
128.36
89.81
77.28
77.07
76.86
54.14
40.85
27.88
25.79
10.13
10.12
Compound 6_Carbon 13 NMR
NH
N
S
N
N
N
Cl
Cl
f1 (ppm)

Compound 6_MS

UB Mass Spectrometry Facility - SmartFormula Report

Analysis Info

Analysis Name	D:\Data\IC_3-21-19\5351a_000001.d	Acquisition Date	3/21/2019 7:10:30 PM
Method	Bruker_11052015	Operator	
Sample Name	5351a	Instrument	solariX
Comment			

Acquisition Parameters

Acquisition Mode	Single MS	Nebulizer gas flow rate	1.2 bar
Polarity	Positive	Drying Gas Temperature	180.0 °C
Broadband Low Mass	147.4 m/z	Drying Gas Flow Rate	4.2 L/min
Broadband High Mass	1500.0 m/z	Calibration Date	Thu Sep 27 10:56:48 2018
Time of Flight to Detector	0.001 sec	Data Acquisition Size	1048576
Ion Accumulation Time	0.100 sec	Data Processing Size (SI)	2097152
Acquired Scans	20	Apodization	Full-Sine
Capillary	6000.0 V		

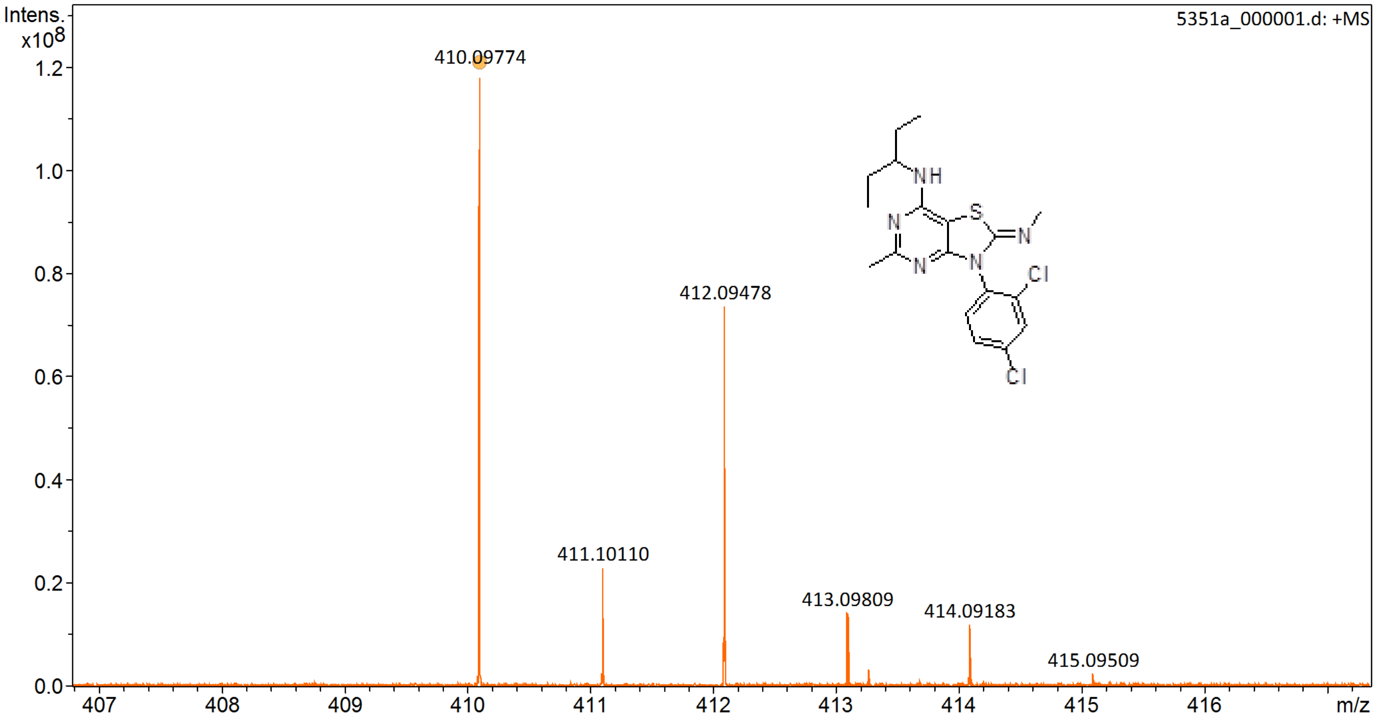

Adduct	Meas. m/z	#	Ion Formula	Score	m/z	err [ppm]	Mean err [ppm]	mSigma	rdb	e⁻ Conf	N-Rule
M+H	410.097742	1	C18H22Cl2N5S	100.00	410.096749	-2.4	-2.5	36.2	9.5	even	ok
M+Na	432.079523	1	C18H21Cl2N5NaS	100.00	432.078693	-1.9	-1.6	30.2	9.5	even	ok

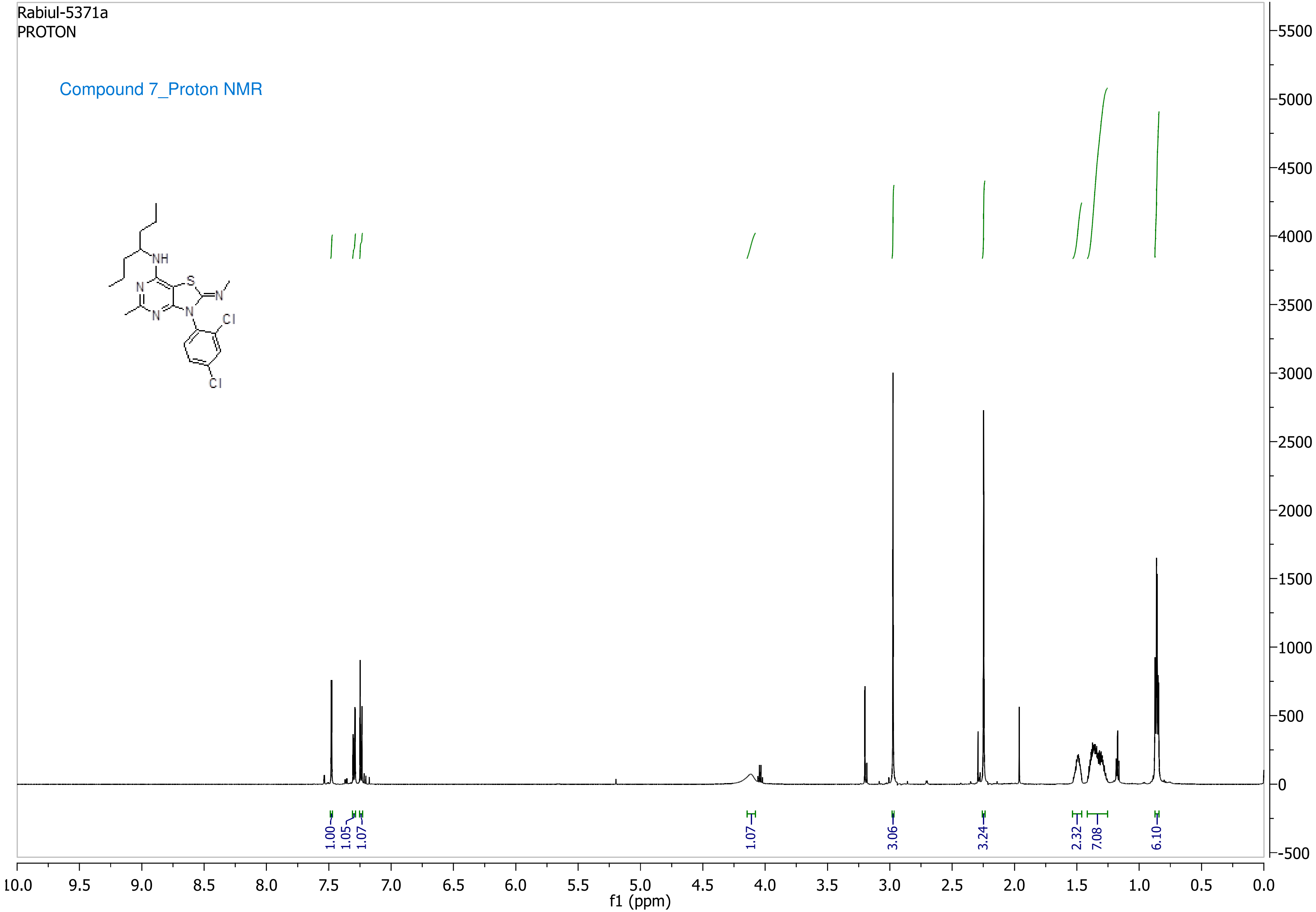
Rabiul-5371a
PROTON
Compound 7_Proton NMR
NH
N
N
S
N
N
Cl
Cl
1.00
1.05
1.07
1.07
3.06
3.24
2.32
7.08
6.10
10.0
9.5
9.0
8.5
8.0
7.5
7.0
6.5
6.0
5.5
5.0
4.5
4.0
3.5
3.0
2.5
2.0
1.5
1.0
0.5
0.0
f1 (ppm)
5500
5000
4500
4000
3500
3000
2500
2000
1500
1000
500
0
-500

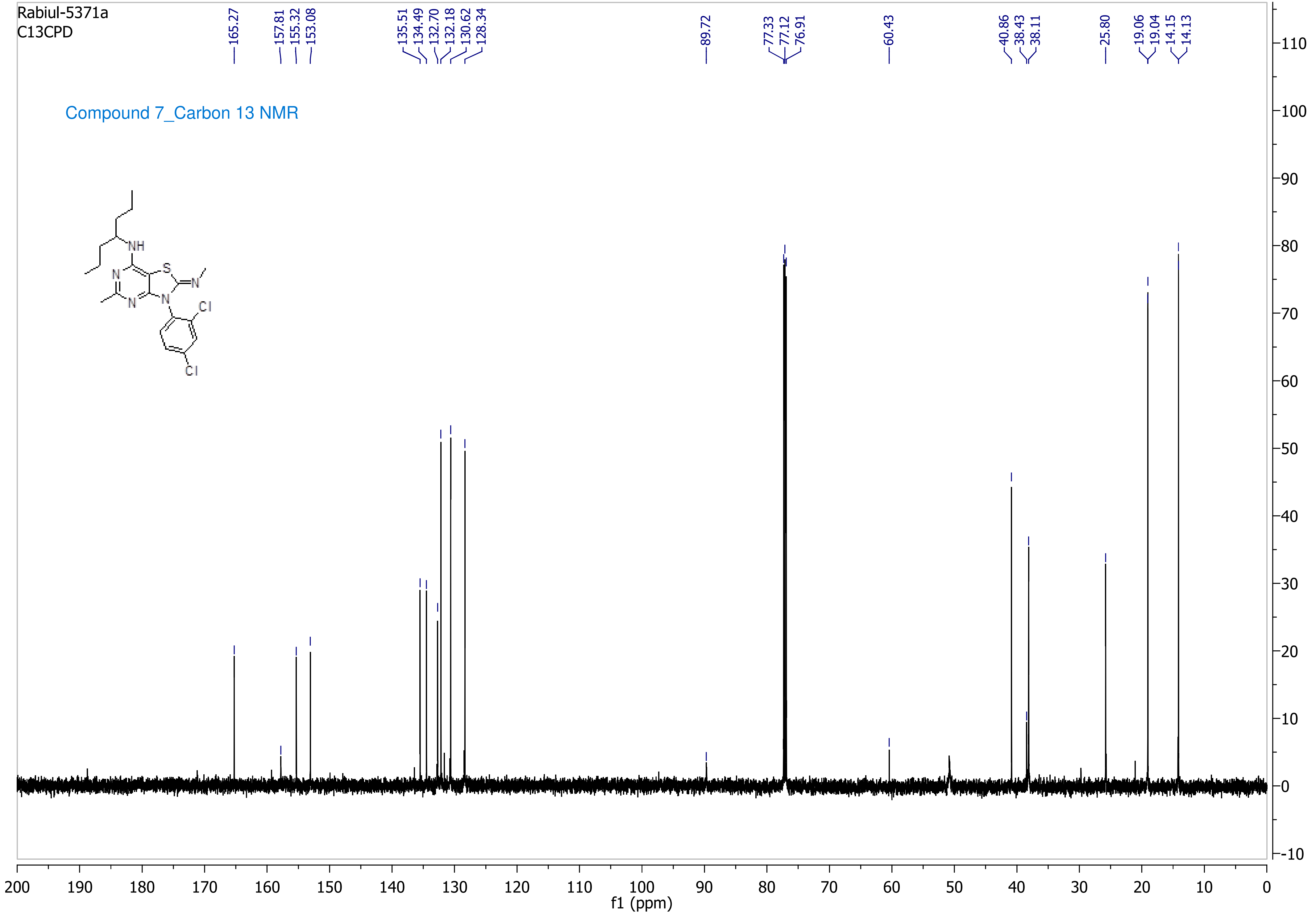
Rabiul-5371a
C13CPD
Compound 7_Carbon 13 NMR
165.27
157.81
155.32
153.08
135.51
134.49
132.70
132.18
130.62
128.34
89.72
77.33
77.12
76.91
60.43
40.86
38.43
38.11
25.80
19.06
19.04
14.15
14.13
NH
S
N
N
N
N
Cl
Cl
f1 (ppm)

Compound 7_MS

UB Mass Spectrometry Facility - SmartFormula Report

Analysis Info

Analysis Name	D:\Data\IC_3-21-19\5371a_000001.d	Acquisition Date	3/21/2019 6:54:32 PM
Method	Bruker_11052015	Operator	
Sample Name	5371a	Instrument	solariX
Comment			

Acquisition Parameters

Acquisition Mode	Single MS	Nebulizer gas flow rate	1.2 bar
Polarity	Positive	Drying Gas Temperature	180.0 °C
Broadband Low Mass	147.4 m/z	Drying Gas Flow Rate	4.2 L/min
Broadband High Mass	1500.0 m/z	Calibration Date	Thu Sep 27 10:56:48 2018
Time of Flight to Detector	0.001 sec	Data Acquisition Size	1048576
Ion Accumulation Time	0.100 sec	Data Processing Size (SI)	2097152
Acquired Scans	20	Apodization	Full-Sine
Capillary	6000.0 V		

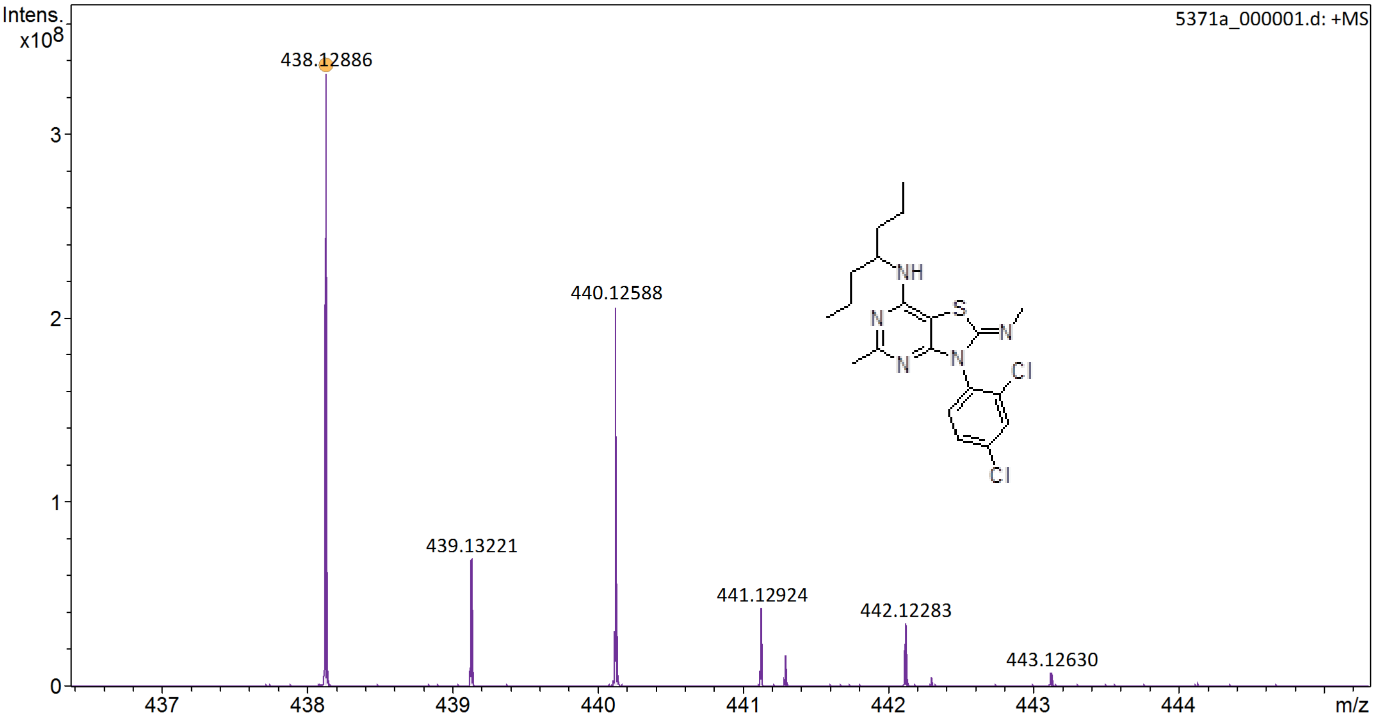

Adduct	Meas. m/z	#	Ion Formula	Score	m/z	err [ppm]	Mean err [ppm]	mSigma	rdb	e⁻ Conf	N-Rule
M+H	438.128862	1	C20H26Cl2N5S	100.00	438.128049	-1.9	-1.9	33.2	9.5	even	ok
M+Na	460.110633	1	C20H25Cl2N5NaS	100.00	460.109993	-1.4	-1.4	17.1	9.5	even	ok

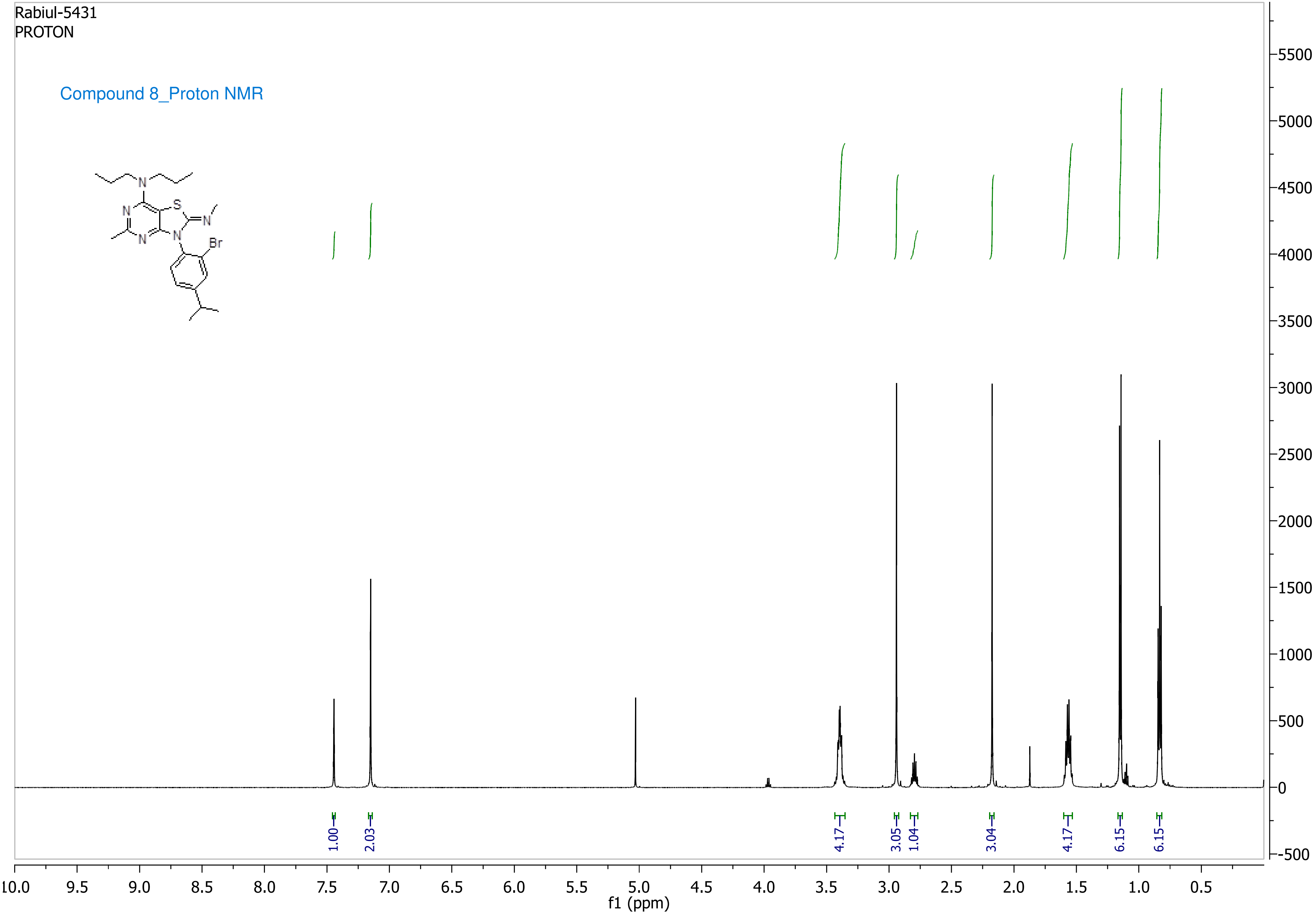
Rabiul-5431
PROTON
Compound 8_Proton NMR
N
S
N
N
N
N
Br
1.00
2.03
4.17
3.05
1.04
3.04
4.17
6.15
6.15
10.0 9.5 9.0 8.5 8.0 7.5 7.0 6.5 6.0 5.5 5.0 4.5 4.0 3.5 3.0 2.5 2.0 1.5 1.0 0.5
f1 (ppm)
5500 5000 4500 4000 3500 3000 2500 2000 1500 1000 500 0 -500

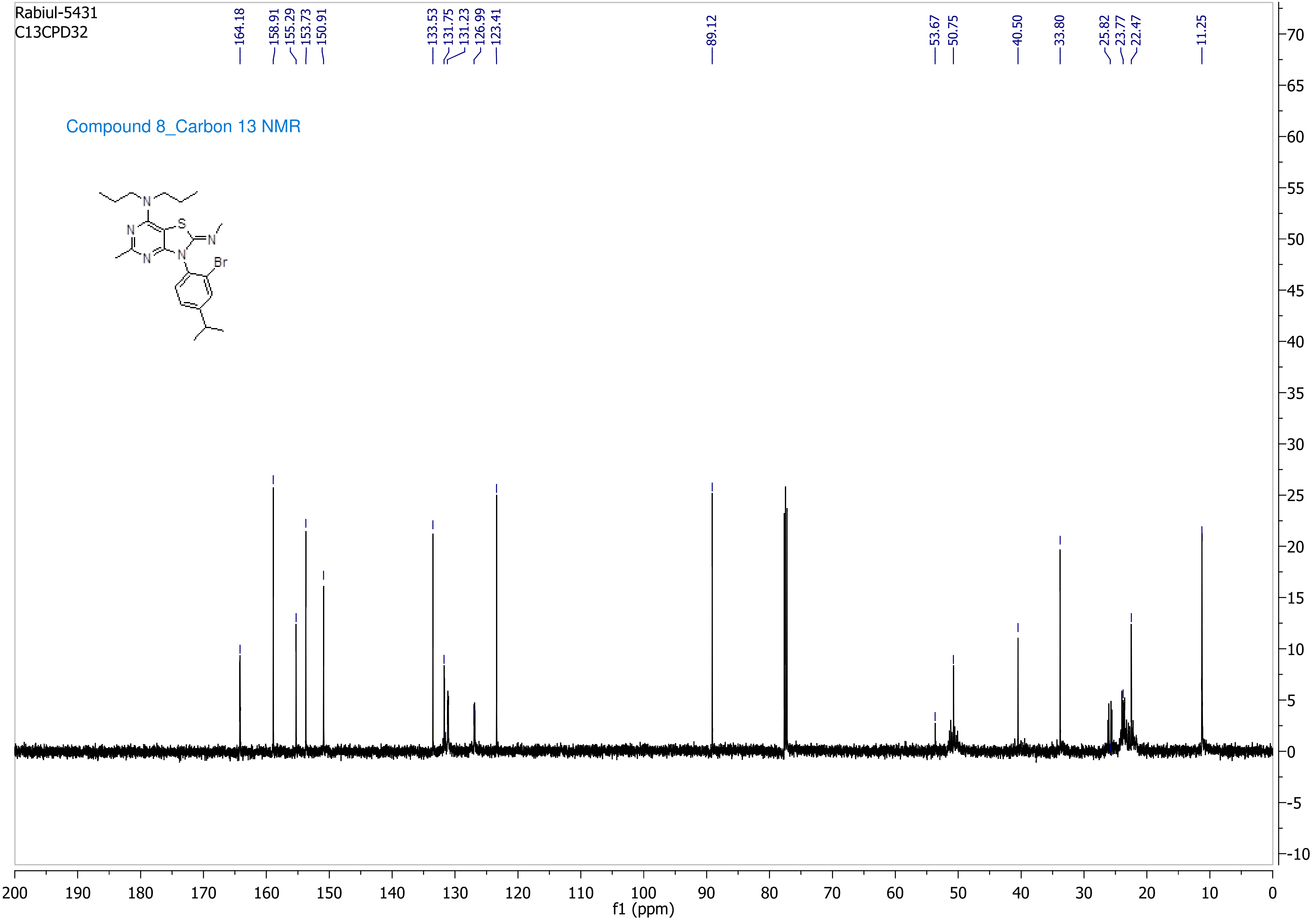
Rabiul-5431
C13CPD32
Compound 8_Carbon 13 NMR
164.18
158.91
155.29
153.73
150.91
133.53
131.75
131.23
126.99
123.41
89.12
53.67
50.75
40.50
33.80
25.82
23.77
22.47
11.25
N
S
N
N
N
Br
f1 (ppm)

UB Mass Spectrometry Facility - SmartFormula Report

Analysis Info

Analysis Name	D:\Data\IC_3-21-19\5431_000001.d	Acquisition Date	3/21/2019 5:58:22 PM
Method	Bruker_11052015	Operator	
Sample Name	5431	Instrument	solariX
Comment			

Acquisition Parameters

Acquisition Mode	Single MS	Nebulizer gas flow rate	1.2 bar
Polarity	Positive	Drying Gas Temperature	180.0 °C
Broadband Low Mass	147.4 m/z	Drying Gas Flow Rate	4.2 L/min
Broadband High Mass	1500.0 m/z	Calibration Date	Thu Sep 27 10:56:48 2018
Time of Flight to Detector	0.001 sec	Data Acquisition Size	1048576
Ion Accumulation Time	0.100 sec	Data Processing Size (SI)	2097152
Acquired Scans	20	Apodization	Full-Sine
Capillary	6000.0 V		

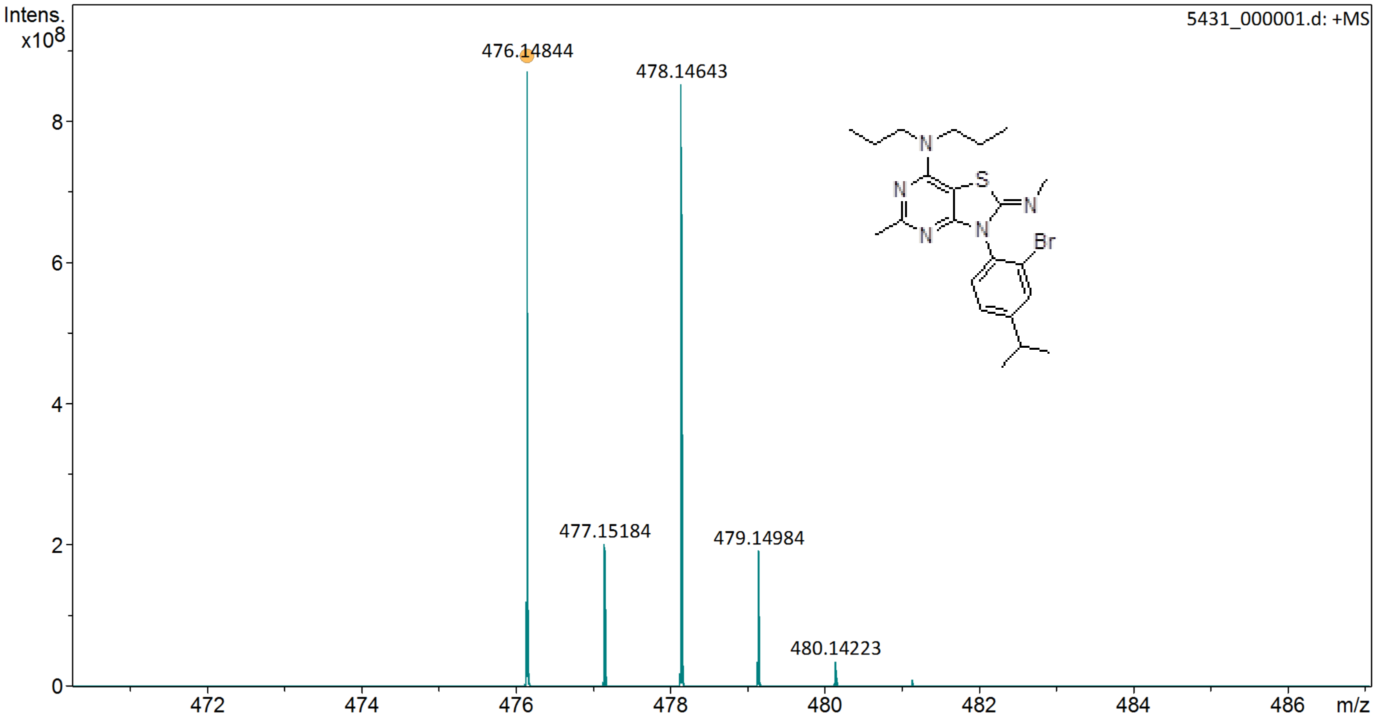

Adduct	Meas. m/z	#	Ion Formula	Score	m/z	err [ppm]	Mean err [ppm]	mSigma	rdb	e⁻ Conf	N-Rule
M+H	476.148442	1	C22H31BrN5S	100.00	476.147806	-1.3	-1.4	21.3	9.5	even	ok
M+Na	498.130223	1	C22H30BrN5NaS	100.00	498.129750	-0.9	-0.9	17.4	9.5	even	ok

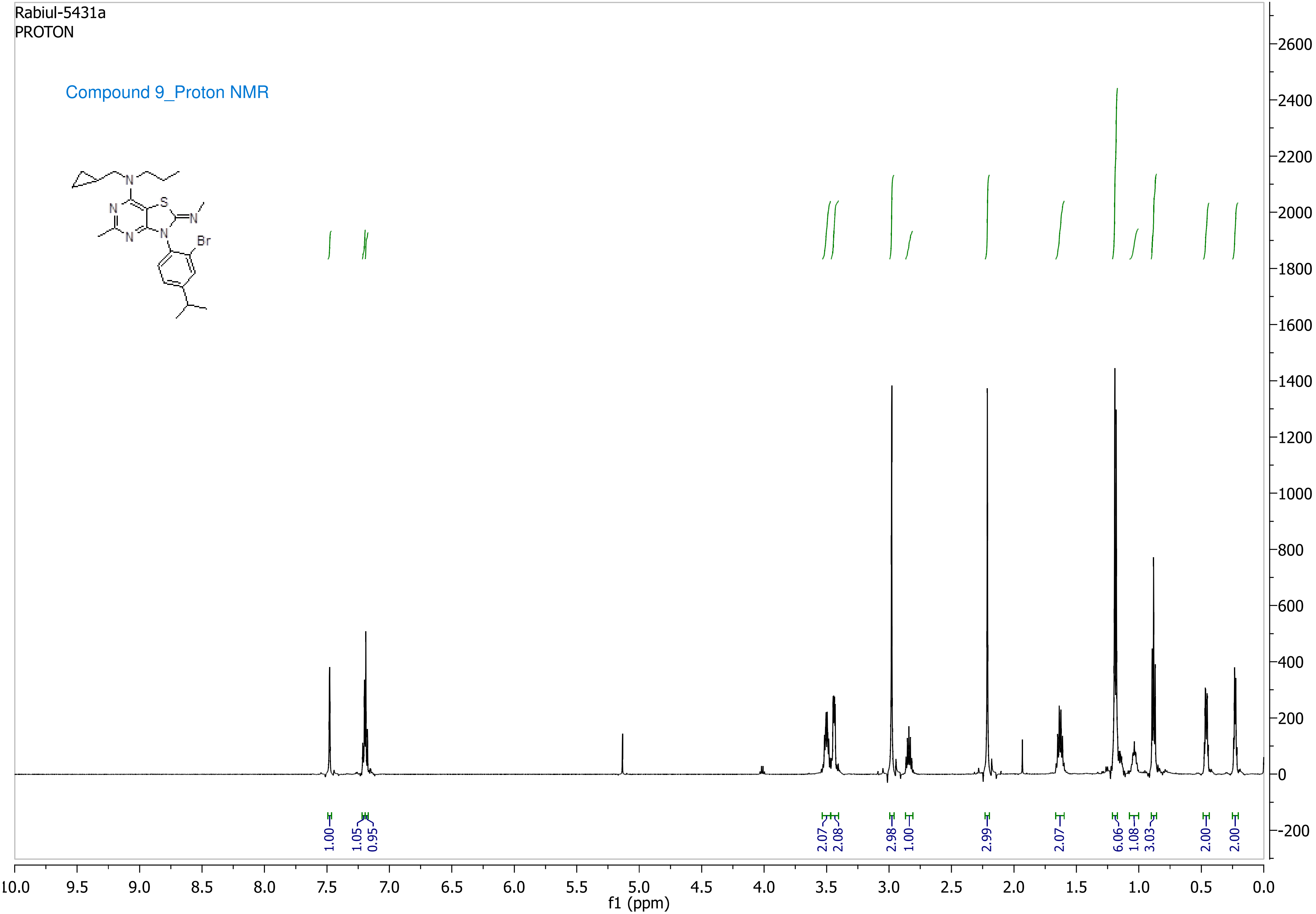
Rabiul-5431a
PROTON
Compound 9_Proton NMR
N
S
N
N
N
N
Br
1.00
1.05
0.95
2.07
2.08
2.98
1.00
2.99
2.07
6.06
1.08
3.03
2.00
2.00
10.0 9.5 9.0 8.5 8.0 7.5 7.0 6.5 6.0 5.5 5.0 4.5 4.0 3.5 3.0 2.5 2.0 1.5 1.0 0.5 0.0
f1 (ppm)
2600 2400 2200 2000 1800 1600 1400 1200 1000 800 600 400 200 0 -200

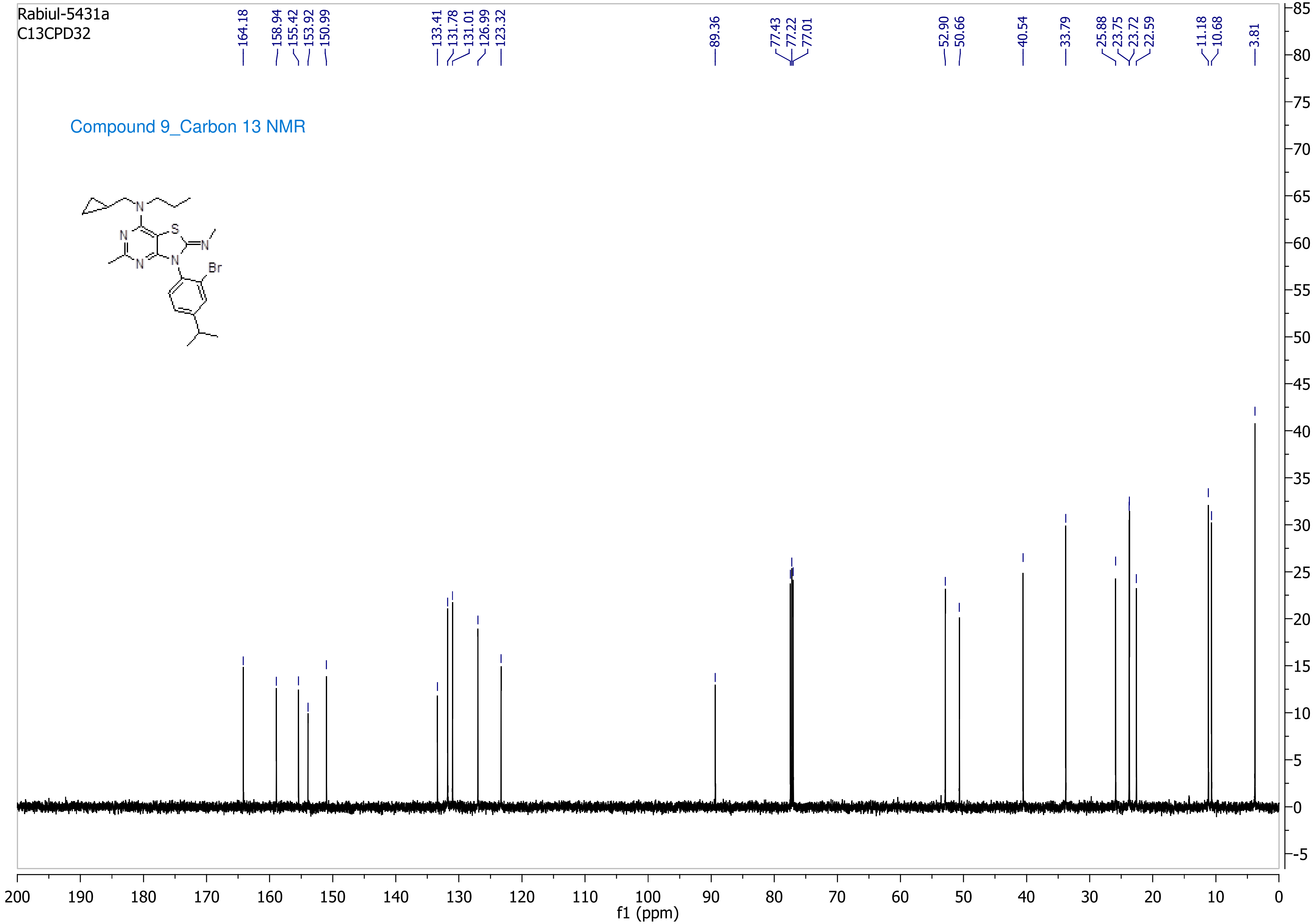
Rabiul-5431a
C13CPD32
Compound 9_Carbon 13 NMR
164.18
158.94
155.42
153.92
150.99
133.41
131.78
131.01
126.99
123.32
89.36
77.43
77.22
77.01
52.90
50.66
40.54
33.79
25.88
23.75
23.72
22.59
11.18
10.68
3.81
N
S
N
N
N
Br
f1 (ppm)

Compound 9_MS

UB Mass Spectrometry Facility - SmartFormula Report

Analysis Info

Analysis Name	D:\Data\IC_3-21-19\5431a_000001.d	Acquisition Date	3/21/2019 6:49:00 PM
Method	Bruker_11052015	Operator	
Sample Name	5431a	Instrument	solariX
Comment			

Acquisition Parameters

Acquisition Mode	Single MS	Nebulizer gas flow rate	1.2 bar
Polarity	Positive	Drying Gas Temperature	180.0 °C
Broadband Low Mass	147.4 m/z	Drying Gas Flow Rate	4.2 L/min
Broadband High Mass	1500.0 m/z	Calibration Date	Thu Sep 27 10:56:48 2018
Time of Flight to Detector	0.001 sec	Data Acquisition Size	1048576
Ion Accumulation Time	0.100 sec	Data Processing Size (SI)	2097152
Acquired Scans	20	Apodization	Full-Sine
Capillary	6000.0 V		

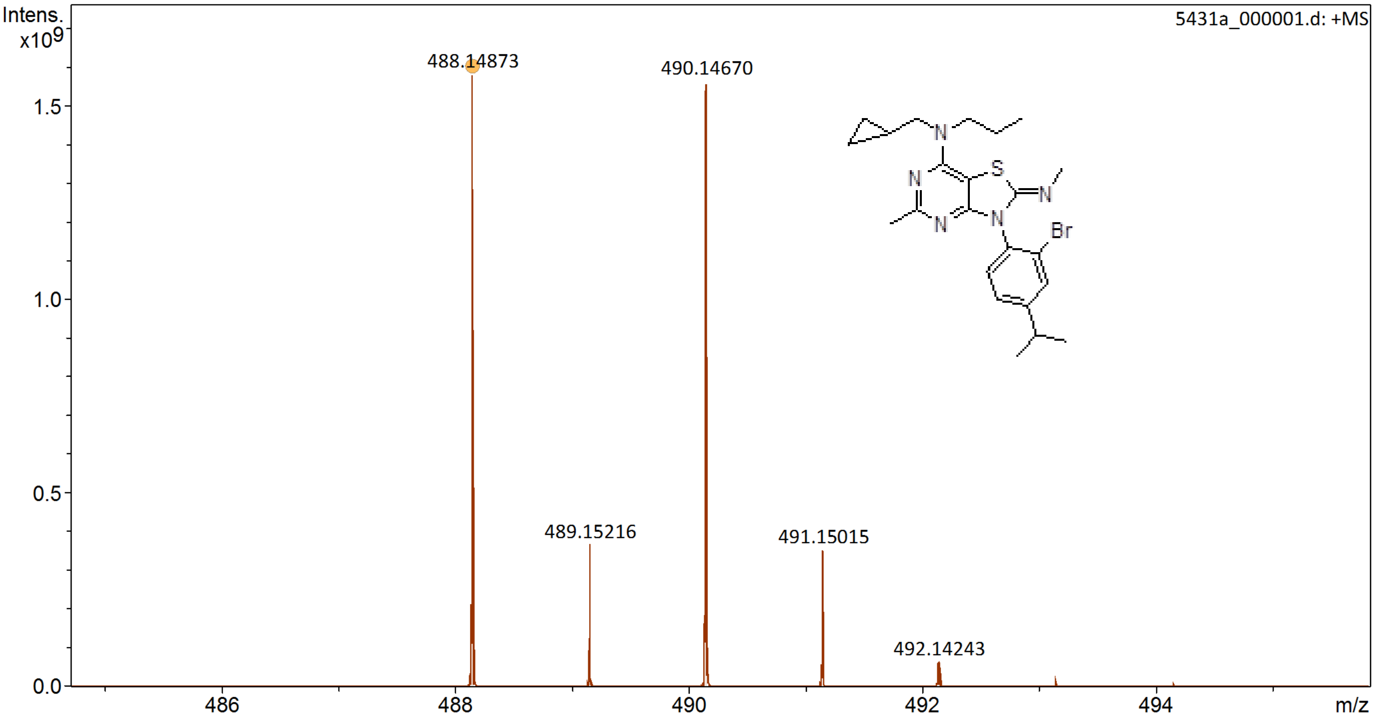

Adduct	Meas. m/z	#	Ion Formula	Score	m/z	err [ppm]	Mean err [ppm]	mSigma	rdb	e⁻ Conf	N-Rule
M+H	488.148727	1	C23H31BrN5S	100.00	488.147806	-1.9	-1.9	18.7	10.5	even	ok
M+Na	510.130636	1	C23H30BrN5NaS	100.00	510.129750	-1.7	-1.7	23.1	10.5	even	ok

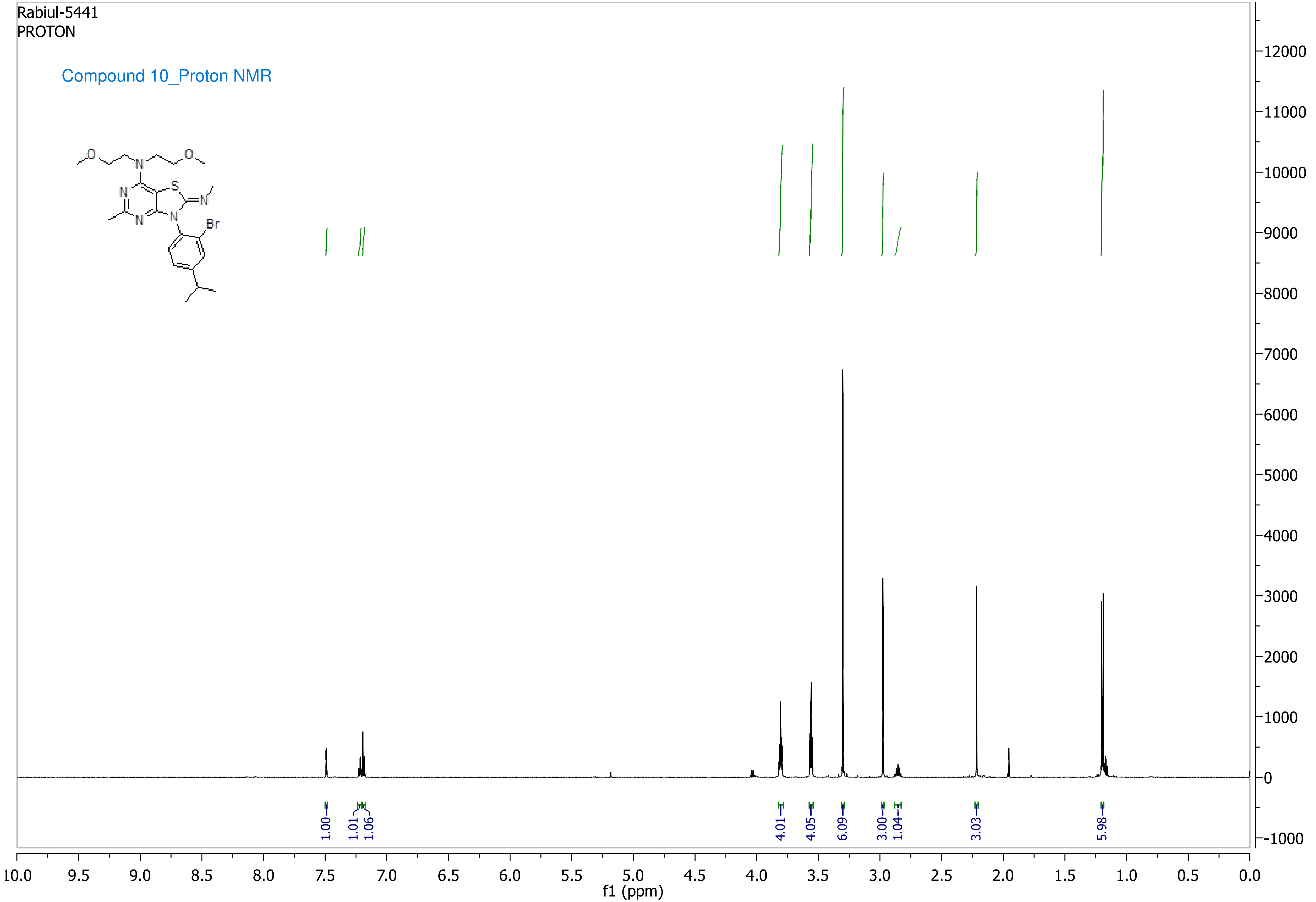
Rabiul-5441
PROTON
Compound 10_Proton NMR
Br
S
N
1.00
1.01
1.06
4.01
4.05
6.09
3.00
1.04
3.03
5.98
12000
11000
10000
9000
8000
7000
6000
5000
4000
3000
2000
1000
0
-1000
10.0 9.5 9.0 8.5 8.0 7.5 7.0 6.5 6.0 5.5 5.0 4.5 4.0 3.5 3.0 2.5 2.0 1.5 1.0 0.5 0.0
f1 (ppm)

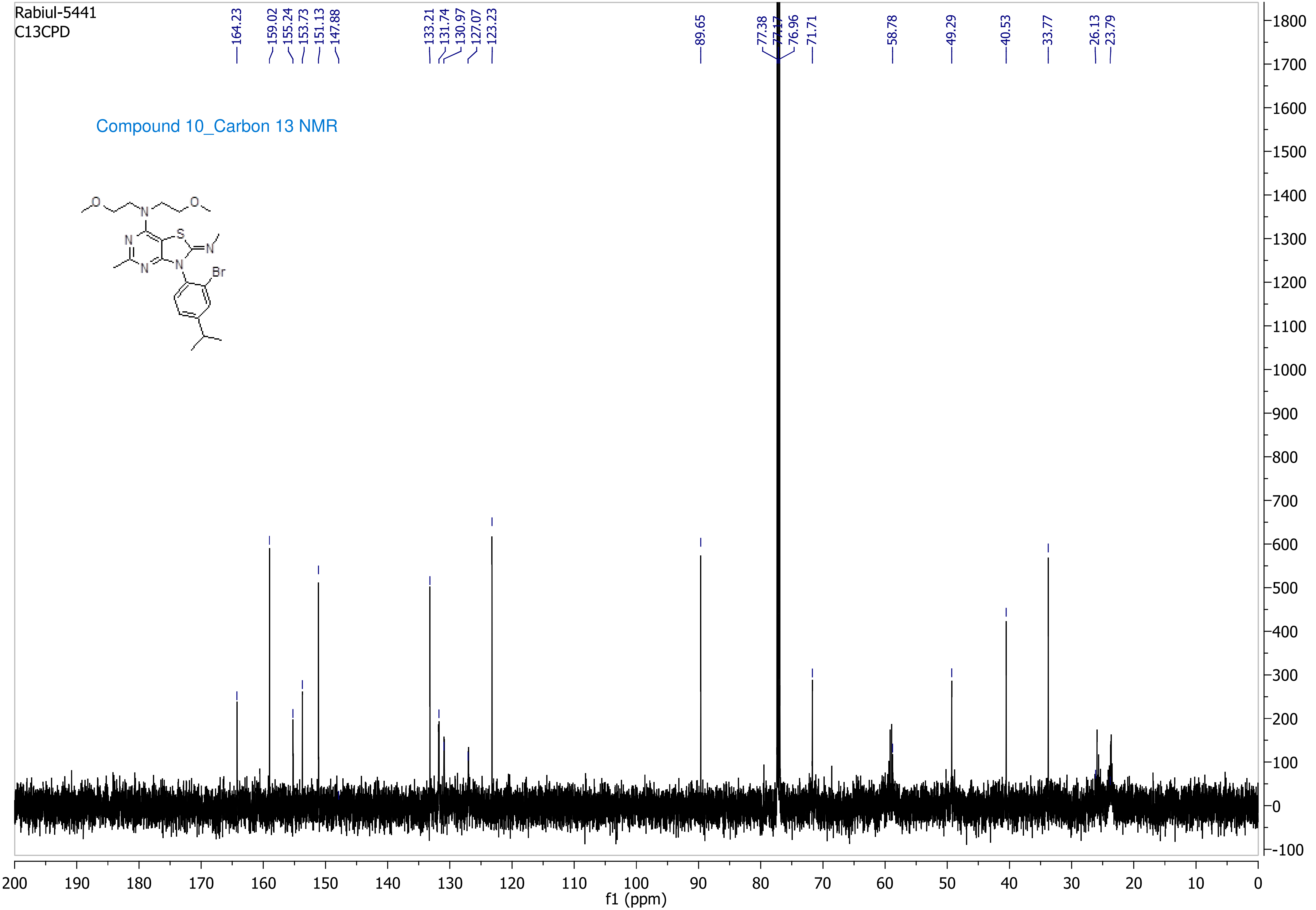
Rabiul-5441
C13CPD
Compound 10_Carbon 13 NMR
164.23
159.02
155.24
153.73
151.13
147.88
133.21
131.74
130.97
127.07
123.23
89.65
77.38
77.17
76.96
71.71
58.78
49.29
40.53
33.77
26.13
23.79
O
N
O
S
N
N
N
N
Br
f1 (ppm)

Compound 10_MS

UB Mass Spectrometry Facility - SmartFormula Report

Analysis Info

Analysis Name	D:\Data\IC_3-21-19\5441_000001.d	Acquisition Date	3/21/2019 6:51:37 PM
Method	Bruker_11052015	Operator	
Sample Name	5441	Instrument	solariX
Comment			

Acquisition Parameters

Acquisition Mode	Single MS	Nebulizer gas flow rate	1.2 bar
Polarity	Positive	Drying Gas Temperature	180.0 °C
Broadband Low Mass	147.4 m/z	Drying Gas Flow Rate	4.2 L/min
Broadband High Mass	1500.0 m/z	Calibration Date	Thu Sep 27 10:56:48 2018
Time of Flight to Detector	0.001 sec	Data Acquisition Size	1048576
Ion Accumulation Time	0.100 sec	Data Processing Size (SI)	2097152
Acquired Scans	20	Apodization	Full-Sine
Capillary	6000.0 V		

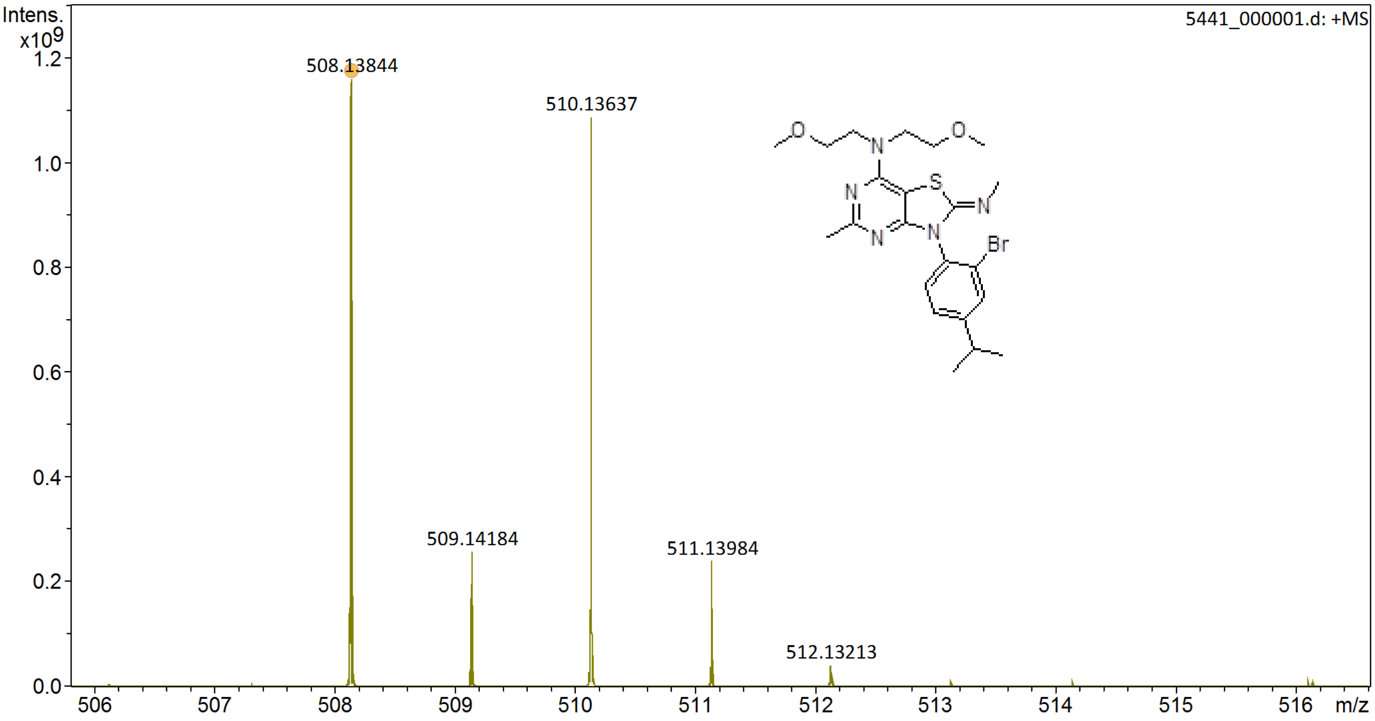

Adduct	Meas. m/z	#	Ion Formula	Score	m/z	err [ppm]	Mean err [ppm]	mSigma	rdb	e⁻ Conf	N-Rule
M+H	508.138436	1	C22H31BrN5O2S	100.00	508.137635	-1.6	-1.8	32.7	9.5	even	ok
M+Na	530.120311	1	C22H30BrN5NaO2S	100.00	530.119579	-1.4	-1.8	41.8	9.5	even	ok

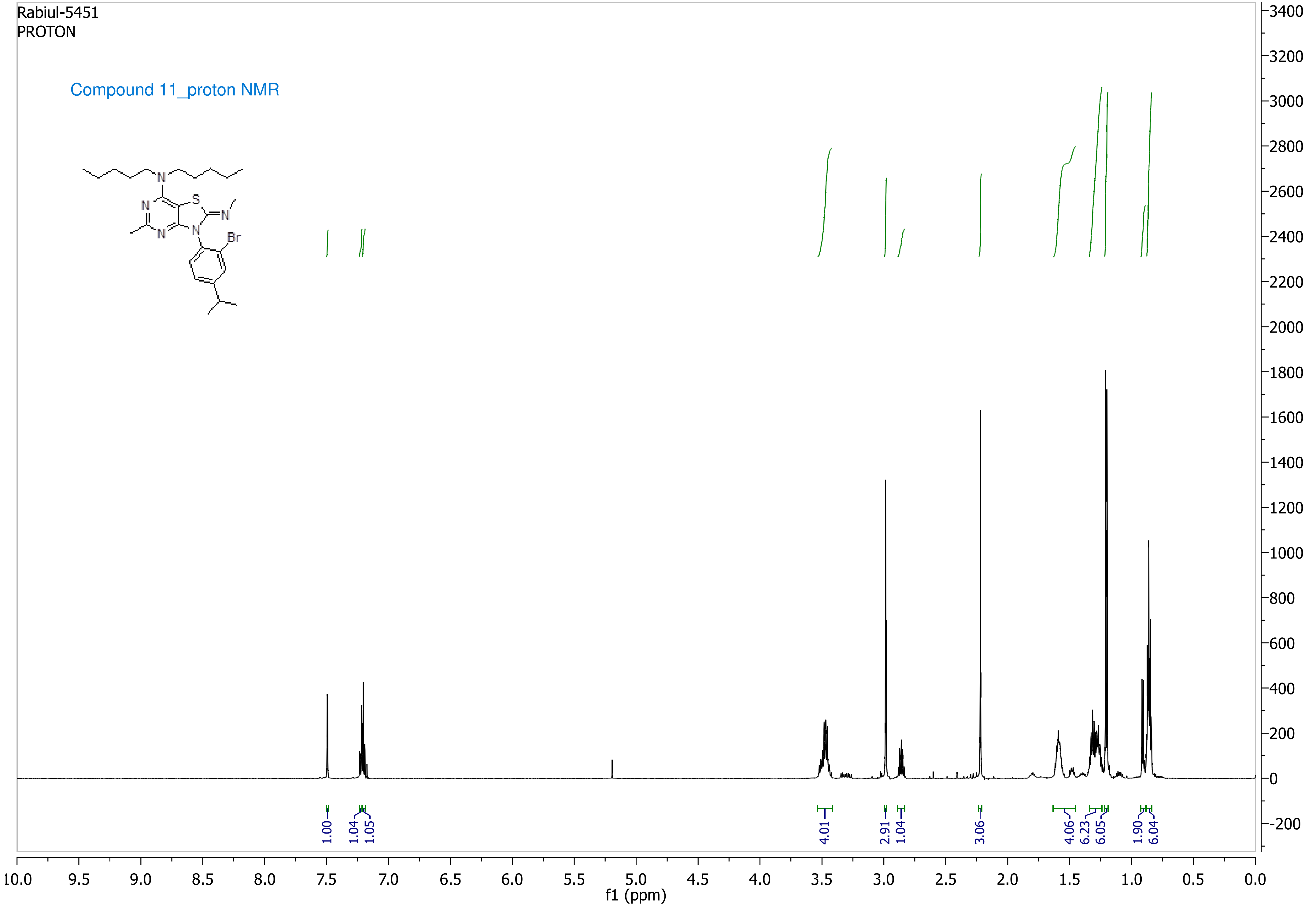

Rabiul-5451
PROTON
Compound 11_proton NMR
N
S
N
N
N
N
Br
1.00
1.04
1.05
4.01
2.91
1.04
3.06
4.06
6.23
6.05
1.90
6.04
3400
3200
3000
2800
2600
2400
2200
2000
1800
1600
1400
1200
1000
800
600
400
200
0
-200
10.0
9.5
9.0
8.5
8.0
7.5
7.0
6.5
6.0
5.5
5.0
4.5
4.0
3.5
3.0
2.5
2.0
1.5
1.0
0.5
0.0
f1 (ppm)

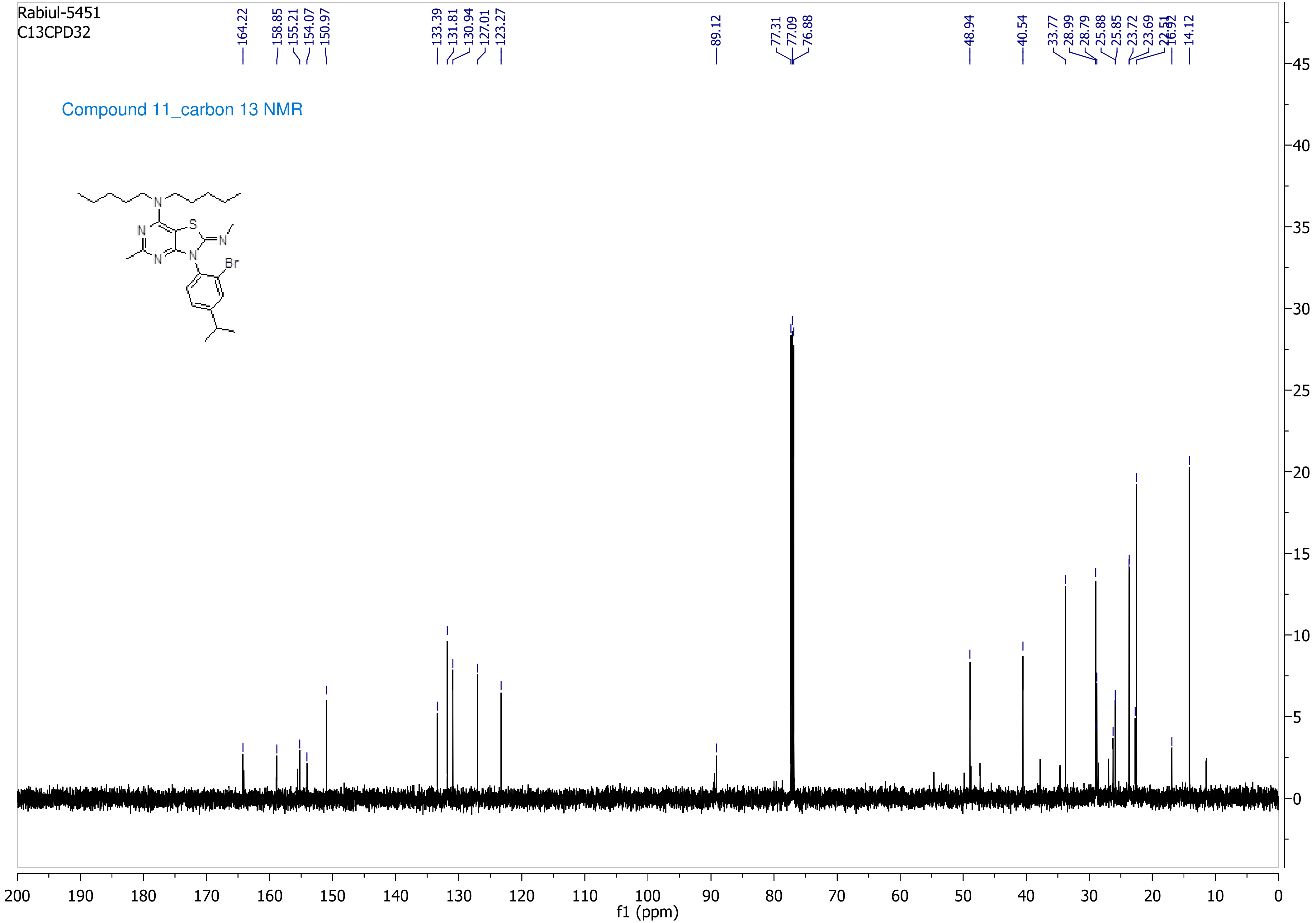

Rabiul-5451
C13CPD32
Compound 11_carbon 13 NMR
164.22
158.85
155.21
154.07
150.97
133.39
131.81
130.94
127.01
123.27
89.12
77.31
77.09
76.88
48.94
40.54
33.77
28.99
28.79
25.88
25.85
23.72
23.69
22.51
16.92
14.12
N
S
N
N
N
Br
f1 (ppm)

UB Mass Spectrometry Facility - SmartFormula Report

Analysis Info

Analysis Name	D:\Data\IC_3-21-19\5451_000001.d	Acquisition Date	3/21/2019 6:41:40 PM
Method	Bruker_11052015	Operator	
Sample Name	5451	Instrument	solariX
Comment			

Acquisition Parameters

Acquisition Mode	Single MS	Nebulizer gas flow rate	1.2 bar
Polarity	Positive	Drying Gas Temperature	180.0 °C
Broadband Low Mass	147.4 m/z	Drying Gas Flow Rate	4.2 L/min
Broadband High Mass	1500.0 m/z	Calibration Date	Thu Sep 27 10:56:48 2018
Time of Flight to Detector	0.001 sec	Data Acquisition Size	1048576
Ion Accumulation Time	0.100 sec	Data Processing Size (SI)	2097152
Acquired Scans	20	Apodization	Full-Sine
Capillary	6000.0 V		

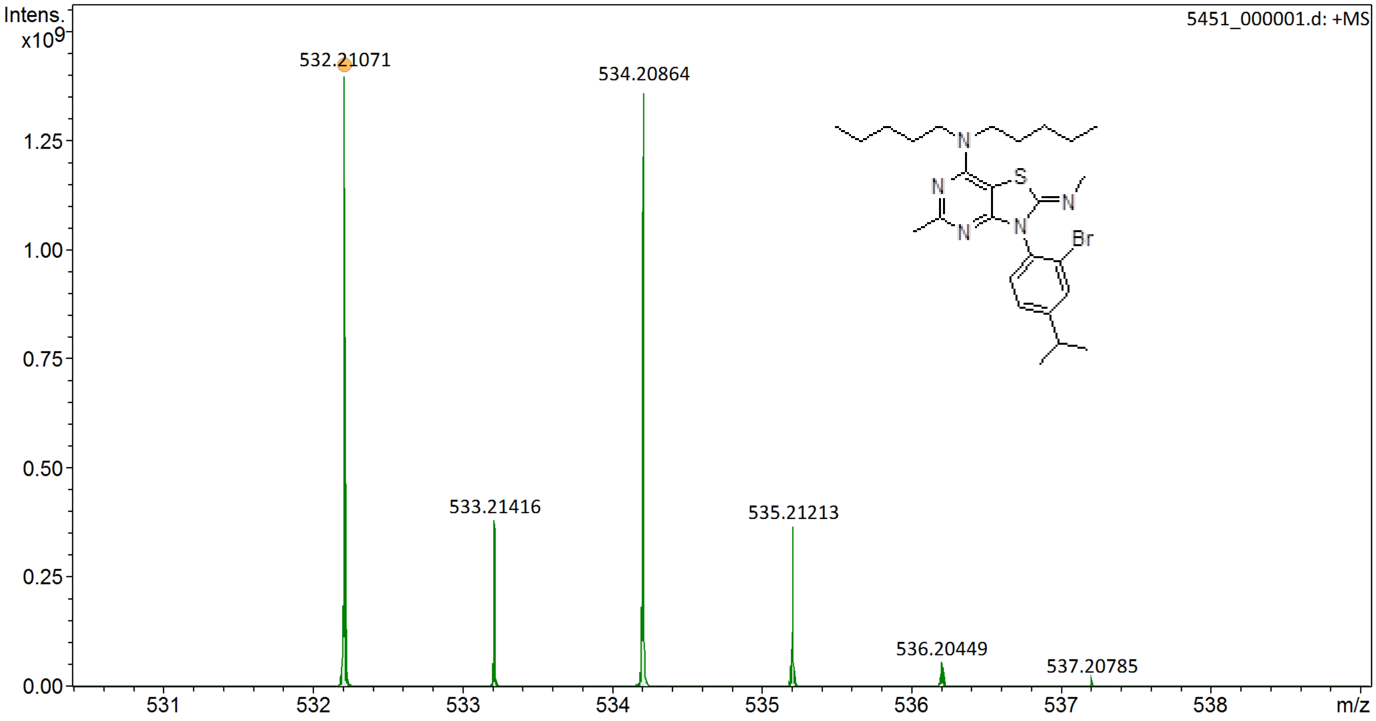

Adduct	Meas. m/z	#	Ion Formula	Score	m/z	err [ppm]	Mean err [ppm]	mSigma	rdb	e⁻ Conf	N-Rule
M+H	532.210706	1	C26H39BrN5S	100.00	532.210406	-0.6	-0.8	29.7	9.5	even	ok
M+Na	554.192640	1	C26H38BrN5NaS	100.00	554.192350	-0.5	-0.7	33.6	9.5	even	ok

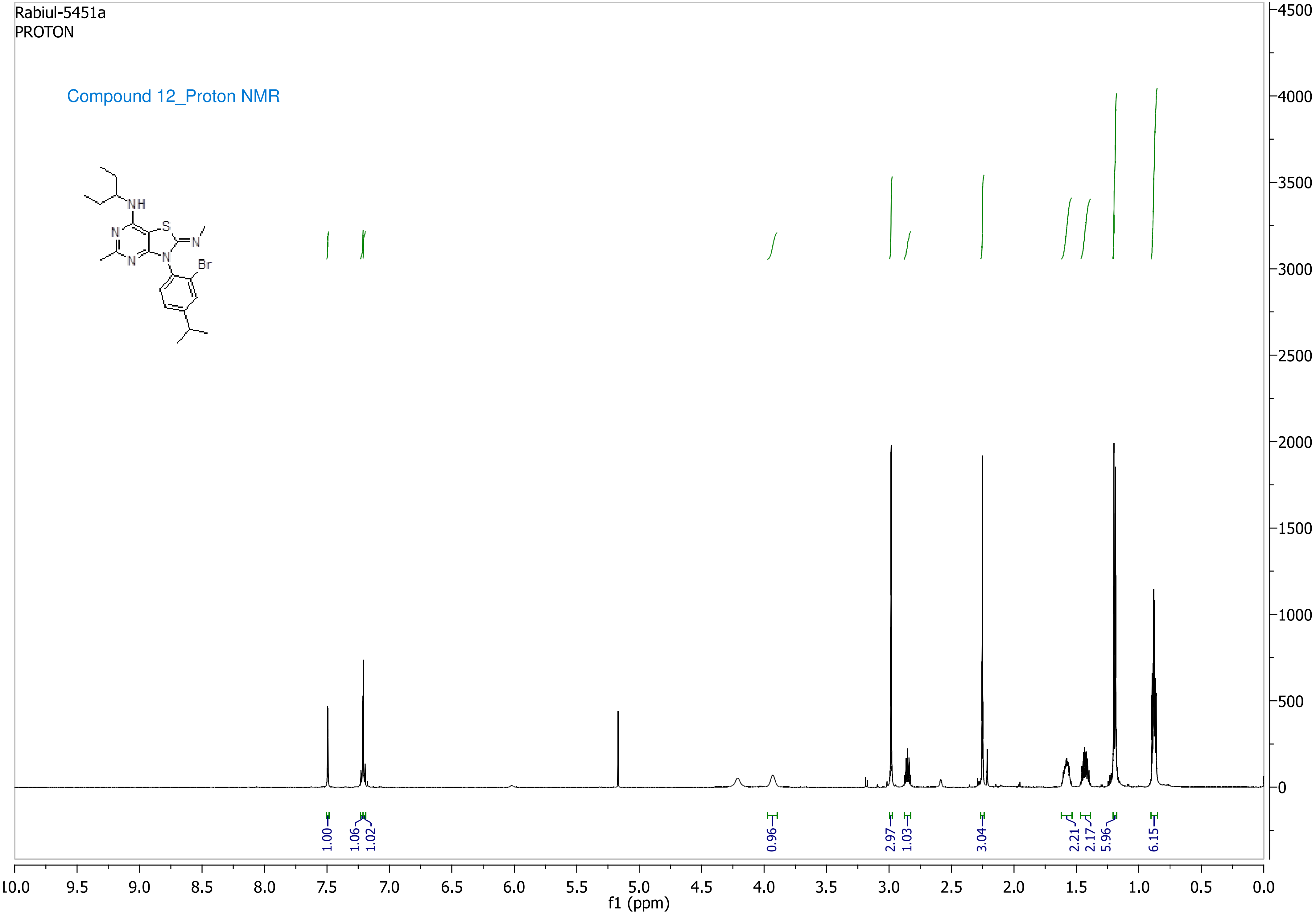
Rabiul-5451a
PROTON
Compound 12_Proton NMR
NH
N
S
N
N
N
Br
1.00
1.06
1.02
0.96
2.97
1.03
3.04
2.21
2.17
5.96
6.15
4500
4000
3500
3000
2500
2000
1500
1000
500
0
10.0
9.5
9.0
8.5
8.0
7.5
7.0
6.5
6.0
5.5
5.0
4.5
4.0
3.5
3.0
2.5
2.0
1.5
1.0
0.5
0.0
f1 (ppm)

Rabiul-5451a
C13CPD32

Compound 12_Carbon 13 NMR

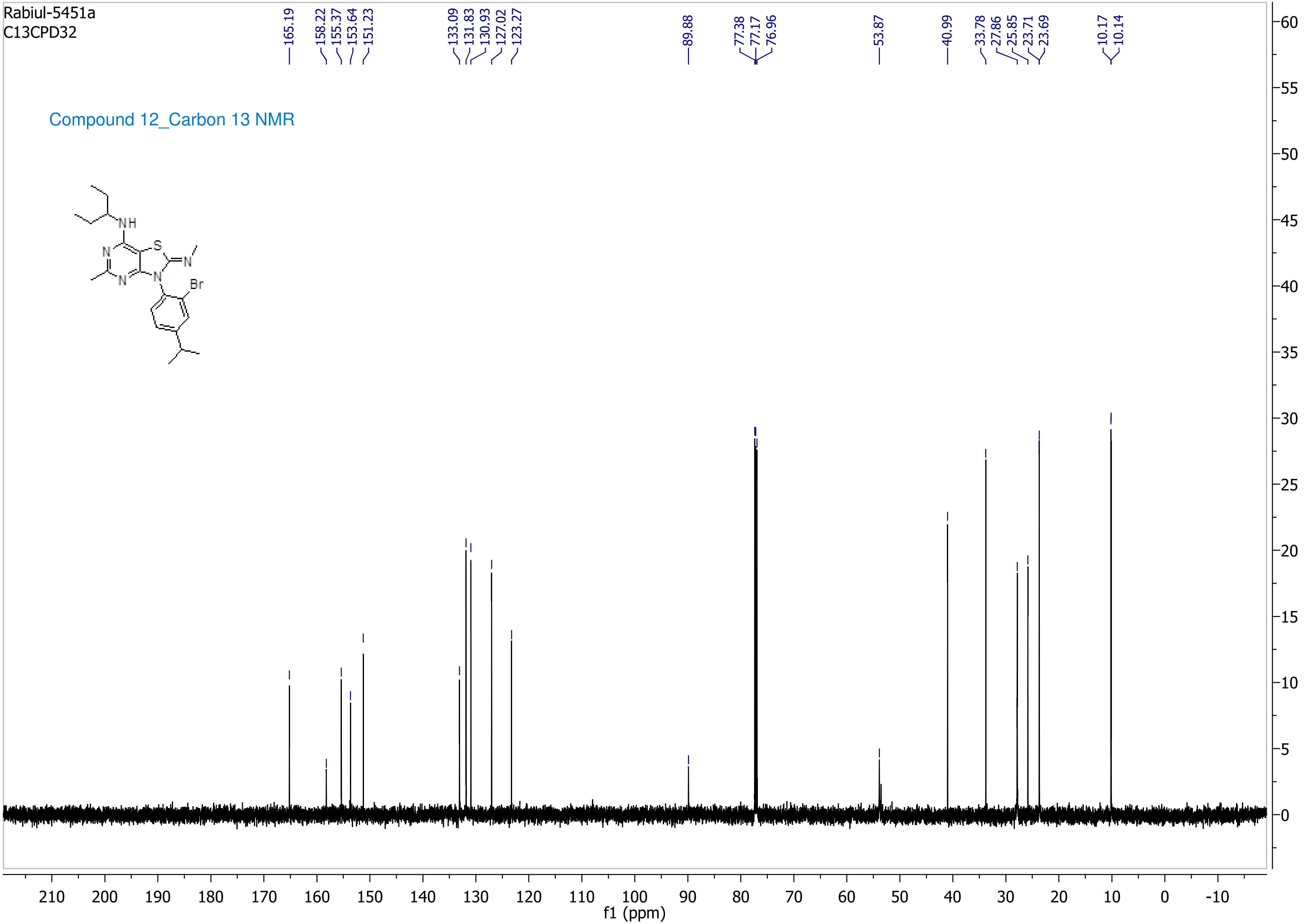

UB Mass Spectrometry Facility - SmartFormula Report

Analysis Info

Analysis Name	D:\Data\IC_3-21-19\5451a_000001.d	Acquisition Date	3/21/2019 6:27:21 PM
Method	Bruker_11052015	Operator	
Sample Name	5451a	Instrument	solariX
Comment			

Acquisition Parameters

Acquisition Mode	Single MS	Nebulizer gas flow rate	1.2 bar
Polarity	Positive	Drying Gas Temperature	180.0 °C
Broadband Low Mass	147.4 m/z	Drying Gas Flow Rate	4.2 L/min
Broadband High Mass	1500.0 m/z	Calibration Date	Thu Sep 27 10:56:48 2018
Time of Flight to Detector	0.001 sec	Data Acquisition Size	1048576
Ion Accumulation Time	0.100 sec	Data Processing Size (SI)	2097152
Acquired Scans	20	Apodization	Full-Sine
Capillary	6000.0 V		

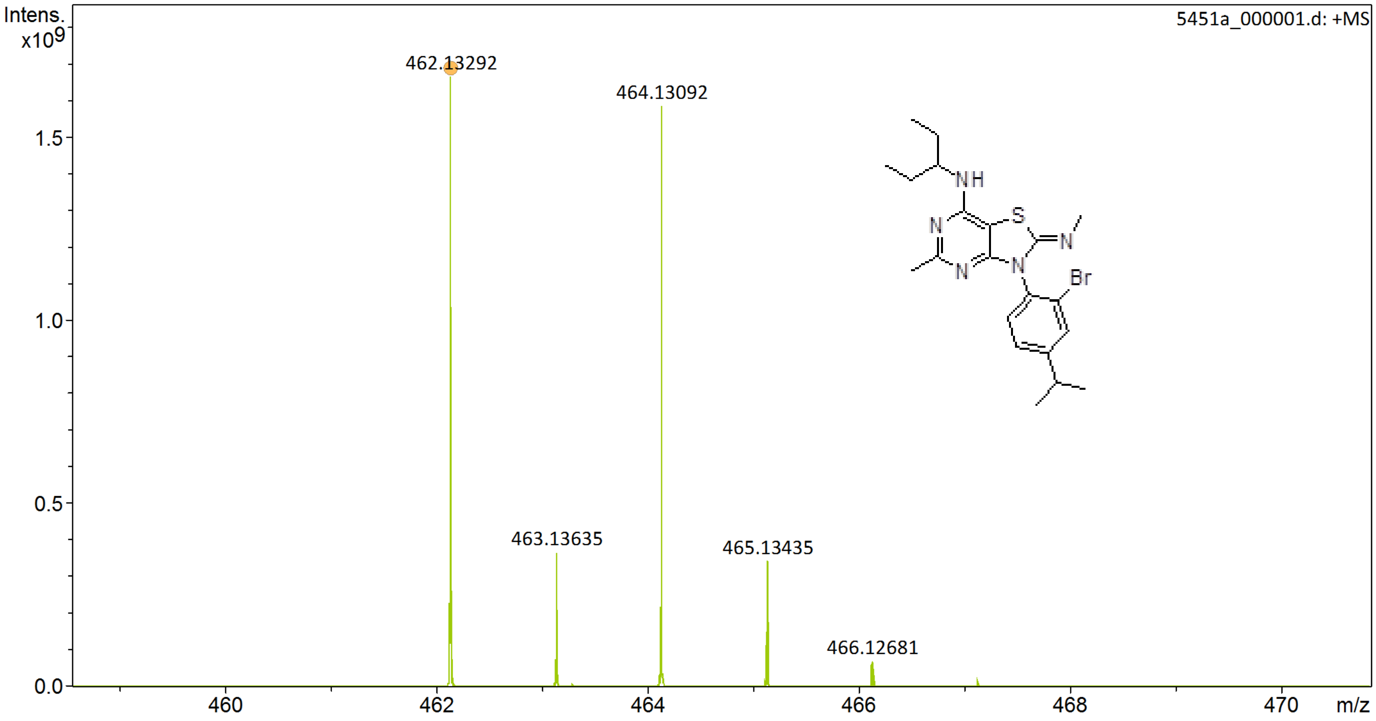

Adduct	Meas. m/z	#	Ion Formula	Score	m/z	err [ppm]	Mean err [ppm]	mSigma	rdb	e⁻ Conf	N-Rule
M+H	462.132924	1	C21H29BrN5S	100.00	462.132155	-1.7	-1.7	26.1	9.5	even	ok
M+Na	484.114796	1	C21H28BrN5NaS	100.00	484.114100	-1.4	-1.4	17.3	9.5	even	ok

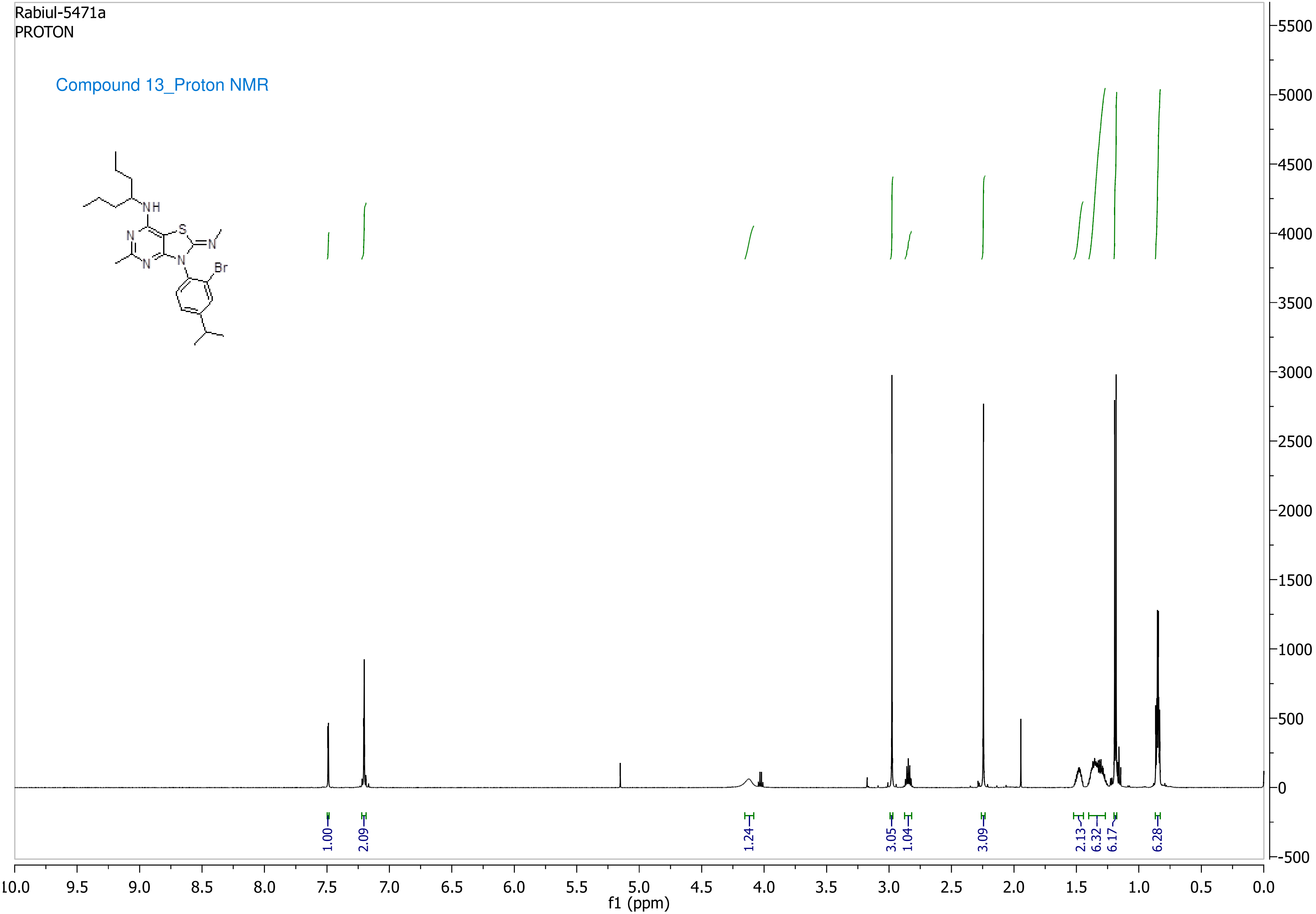
Rabiul-5471a
PROTON
Compound 13_Proton NMR
NH
N
S
N
N
N
Br
1.00
2.09
1.24
3.05
1.04
3.09
2.13
6.32
6.17
6.28
10.0 9.5 9.0 8.5 8.0 7.5 7.0 6.5 6.0 5.5 5.0 4.5 4.0 3.5 3.0 2.5 2.0 1.5 1.0 0.5 0.0
f1 (ppm)
5500 5000 4500 4000 3500 3000 2500 2000 1500 1000 500 0 -500

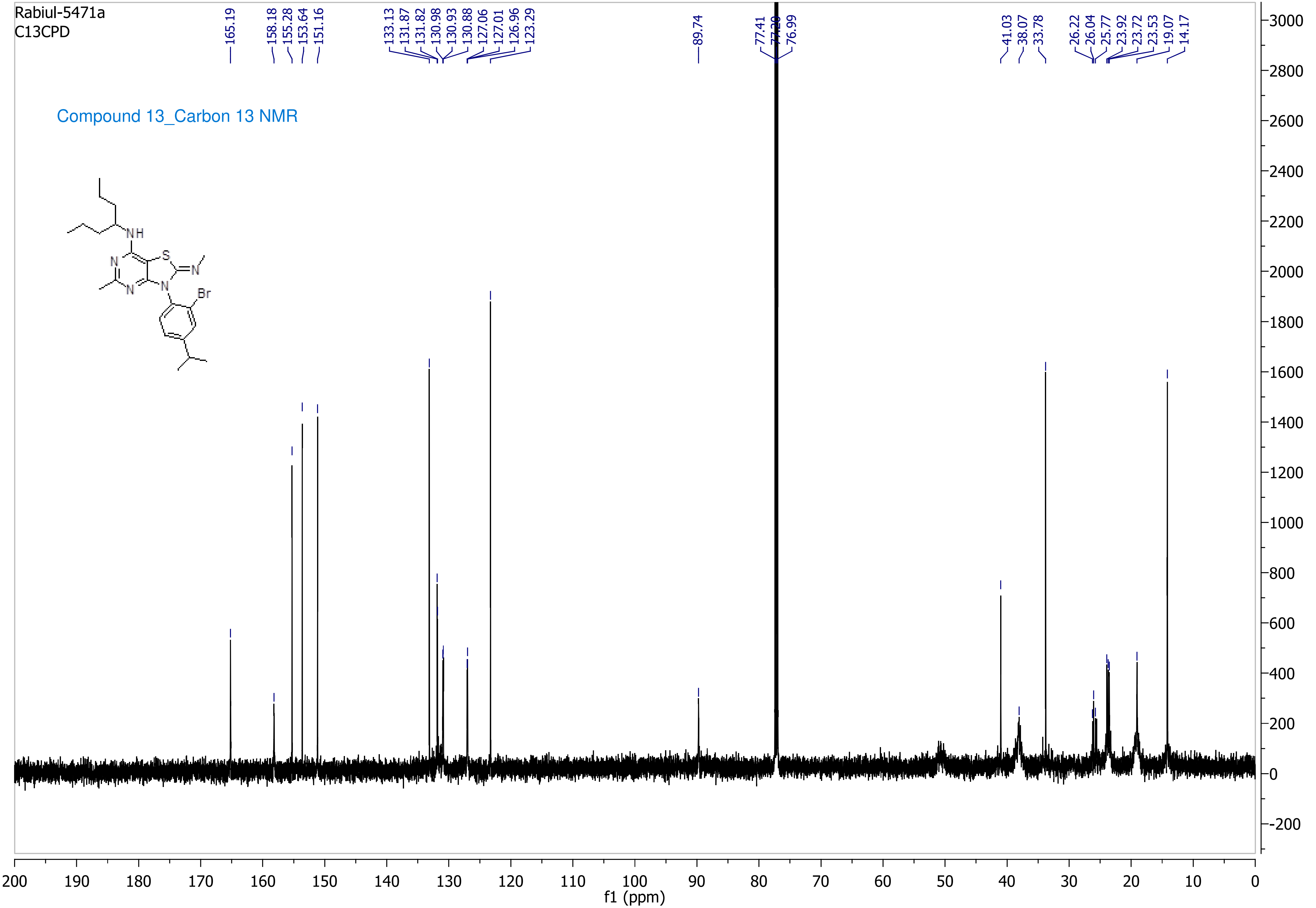
Rabiul-5471a
C13CPD
Compound 13_Carbon 13 NMR
165.19
158.18
155.28
153.64
151.16
133.13
131.87
131.82
130.98
130.93
130.88
127.06
127.01
126.96
123.29
89.74
77.41
76.99
41.03
38.07
33.78
26.22
26.04
25.77
23.92
23.72
23.53
19.07
14.17
NH
S
N
N
N
N
Br
f1 (ppm)

Compound 13_MS

UB Mass Spectrometry Facility - SmartFormula Report

Analysis Info

Analysis Name	D:\Data\IC_3-21-19\5471a_000001.d	Acquisition Date	3/21/2019 6:44:55 PM
Method	Bruker_11052015	Operator	
Sample Name	5471a	Instrument	solariX
Comment			

Acquisition Parameters

Acquisition Mode	Single MS	Nebulizer gas flow rate	1.2 bar
Polarity	Positive	Drying Gas Temperature	180.0 °C
Broadband Low Mass	147.4 m/z	Drying Gas Flow Rate	4.2 L/min
Broadband High Mass	1500.0 m/z	Calibration Date	Thu Sep 27 10:56:48 2018
Time of Flight to Detector	0.001 sec	Data Acquisition Size	1048576
Ion Accumulation Time	0.100 sec	Data Processing Size (SI)	2097152
Acquired Scans	20	Apodization	Full-Sine
Capillary	6000.0 V		

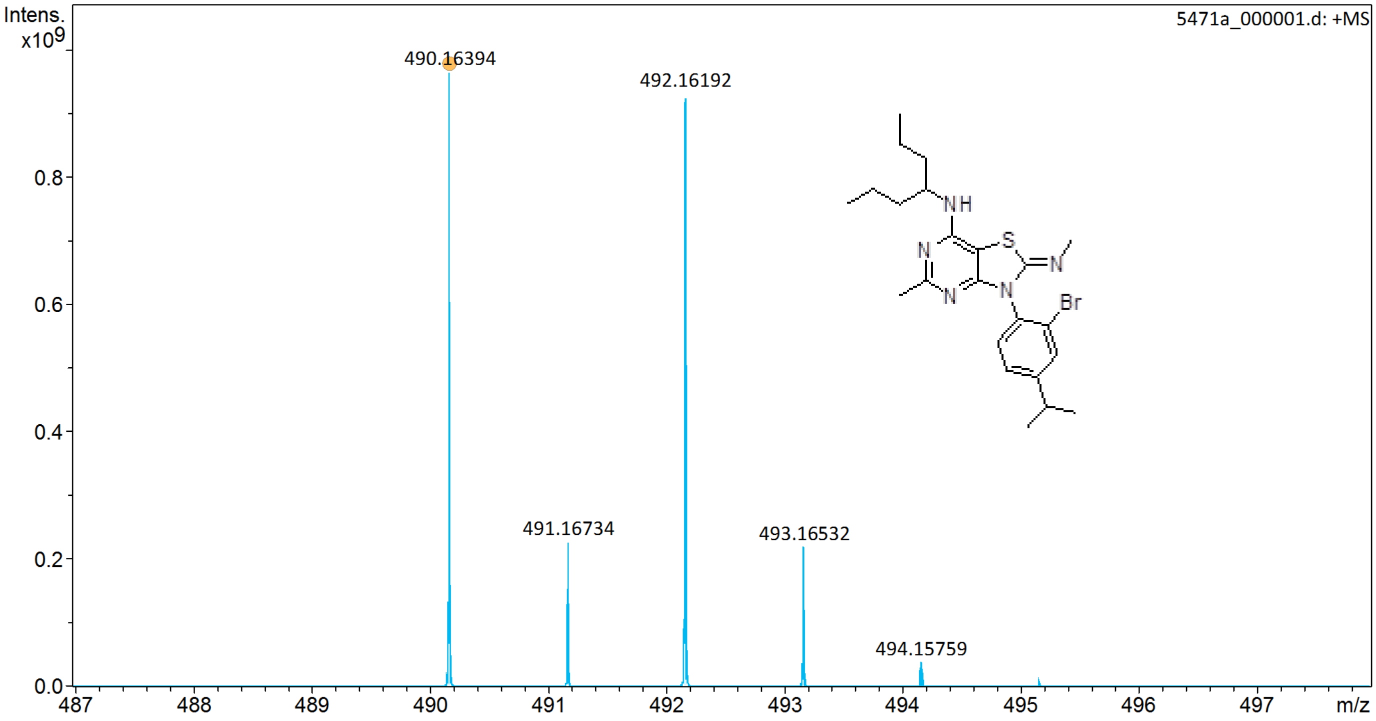

Adduct	Meas. m/z	#	Ion Formula	Score	m/z	err [ppm]	Mean err [ppm]	mSigma	rdb	e¯ Conf	N-Rule
M+H	490.163944	1	C23H33BrN5S	100.00	490.163456	-1.0	-1.0	22.6	9.5	even	ok
M+Na	512.145744	1	C23H32BrN5NaS	100.00	512.145400	-0.7	-1.1	32.7	9.5	even	ok

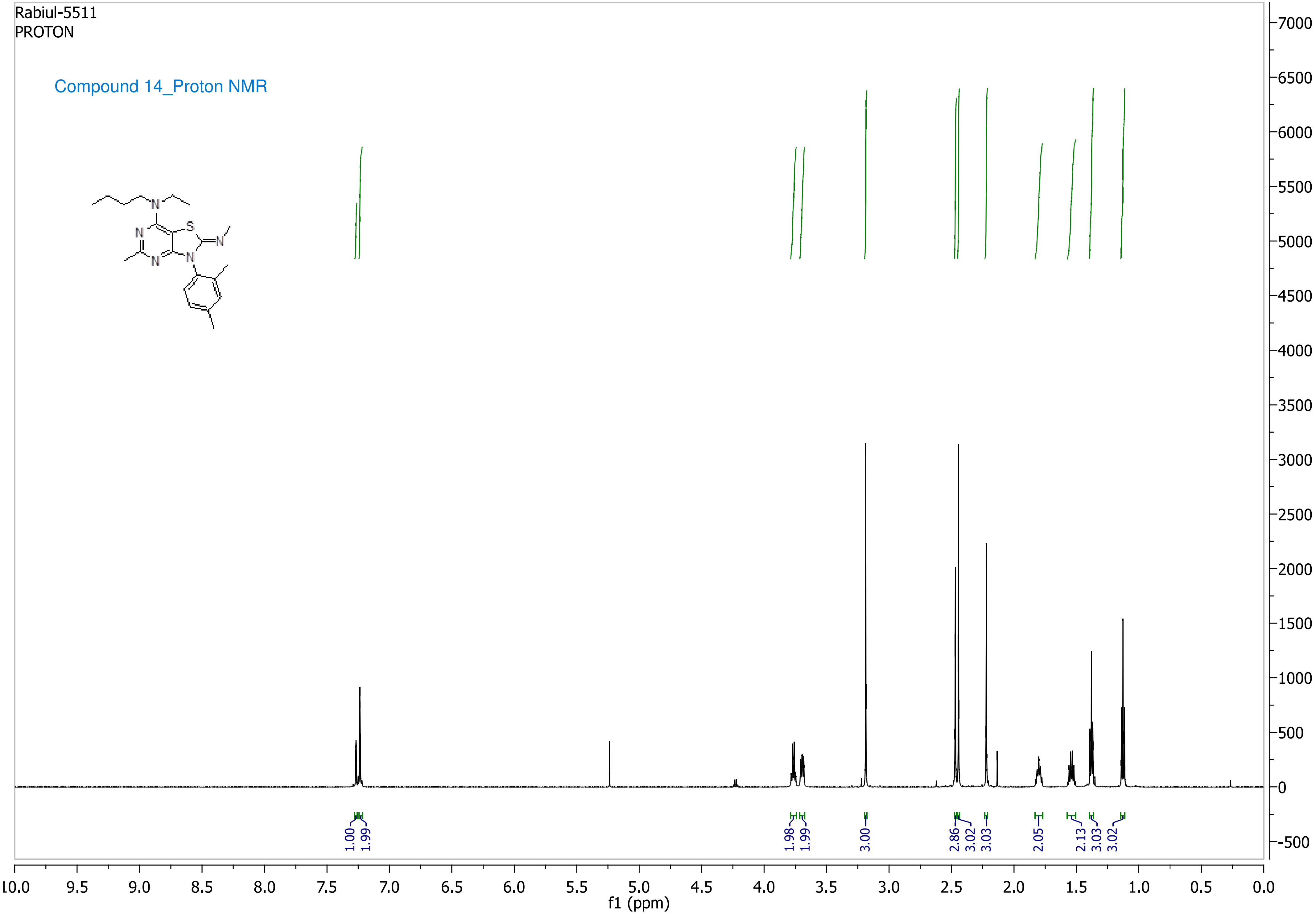
Rabiul-5511
PROTON
Compound 14_Proton NMR
N
S
N
N
N
N
1.00
1.99
1.98
1.99
3.00
2.86
3.02
3.03
2.05
2.13
3.03
3.02
10.0 9.5 9.0 8.5 8.0 7.5 7.0 6.5 6.0 5.5 5.0 4.5 4.0 3.5 3.0 2.5 2.0 1.5 1.0 0.5 0.0
f1 (ppm)
7000 6500 6000 5500 5000 4500 4000 3500 3000 2500 2000 1500 1000 500 0 -500

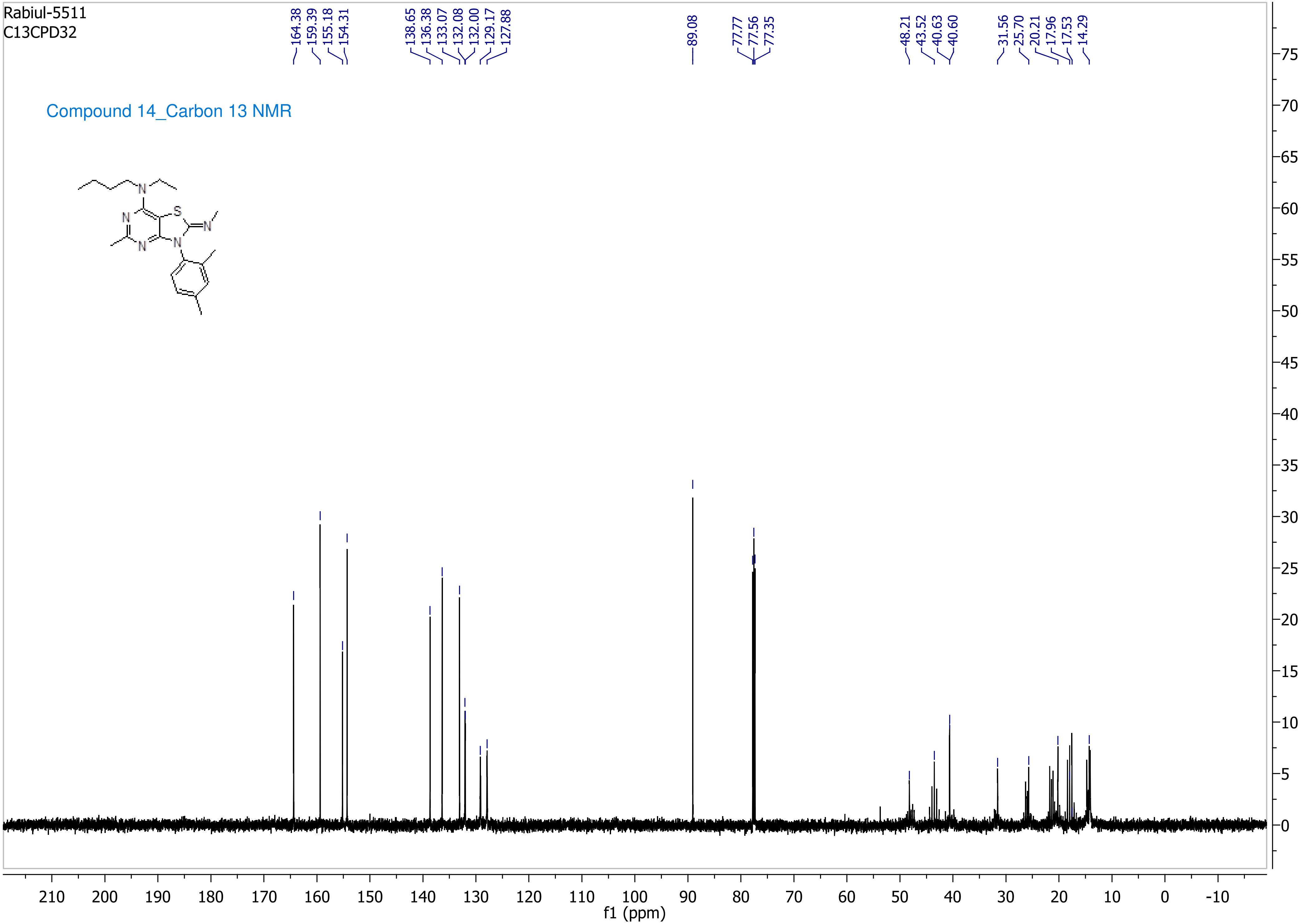
Rabiul-5511
C13CPD32
Compound 14_Carbon 13 NMR
164.38
159.39
155.18
154.31
138.65
136.38
133.07
132.08
132.00
129.17
127.88
89.08
77.77
77.56
77.35
48.21
43.52
40.63
40.60
31.56
25.70
20.21
17.96
17.53
14.29
S
N
f1 (ppm)

Compound 14_MS

UB Mass Spectrometry Facility - SmartFormula Report

Analysis Info

Analysis Name	D:\Data\IC_3-21-19\5511_000003.d	Acquisition Date	3/21/2019 6:11:07 PM
Method	Bruker_11052015	Operator	
Sample Name	5511	Instrument	solariX
Comment			

Acquisition Parameters

Acquisition Mode	Single MS	Nebulizer gas flow rate	1.2 bar
Polarity	Positive	Drying Gas Temperature	180.0 °C
Broadband Low Mass	147.4 m/z	Drying Gas Flow Rate	4.2 L/min
Broadband High Mass	1500.0 m/z	Calibration Date	Thu Sep 27 10:56:48 2018
Time of Flight to Detector	0.001 sec	Data Acquisition Size	1048576
Ion Accumulation Time	0.100 sec	Data Processing Size (SI)	2097152
Acquired Scans	20	Apodization	Full-Sine
Capillary	6000.0 V		

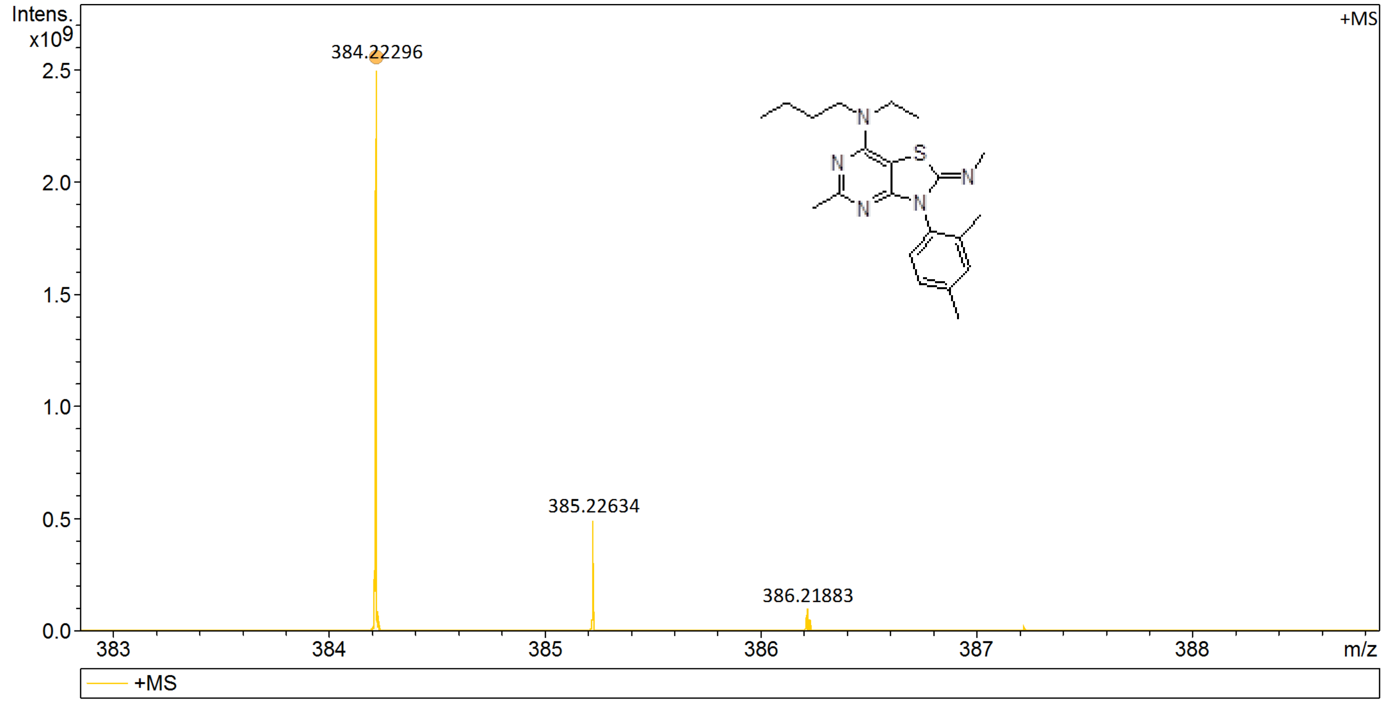

Adduct	Meas. m/z	#	Ion Formula	Score	m/z	err [ppm]	Mean err [ppm]	mSigma	rdb	e⁻ Conf	N-Rule
M+H	384.222957	1	C21H30N5S	100.00	384.221643	-3.4	438.7	21.0	9.5	even	ok
M+Na	406.204814	1	C21H29N5NaS	100.00	406.203588	-3.0	415.1	15.7	9.5	even	ok

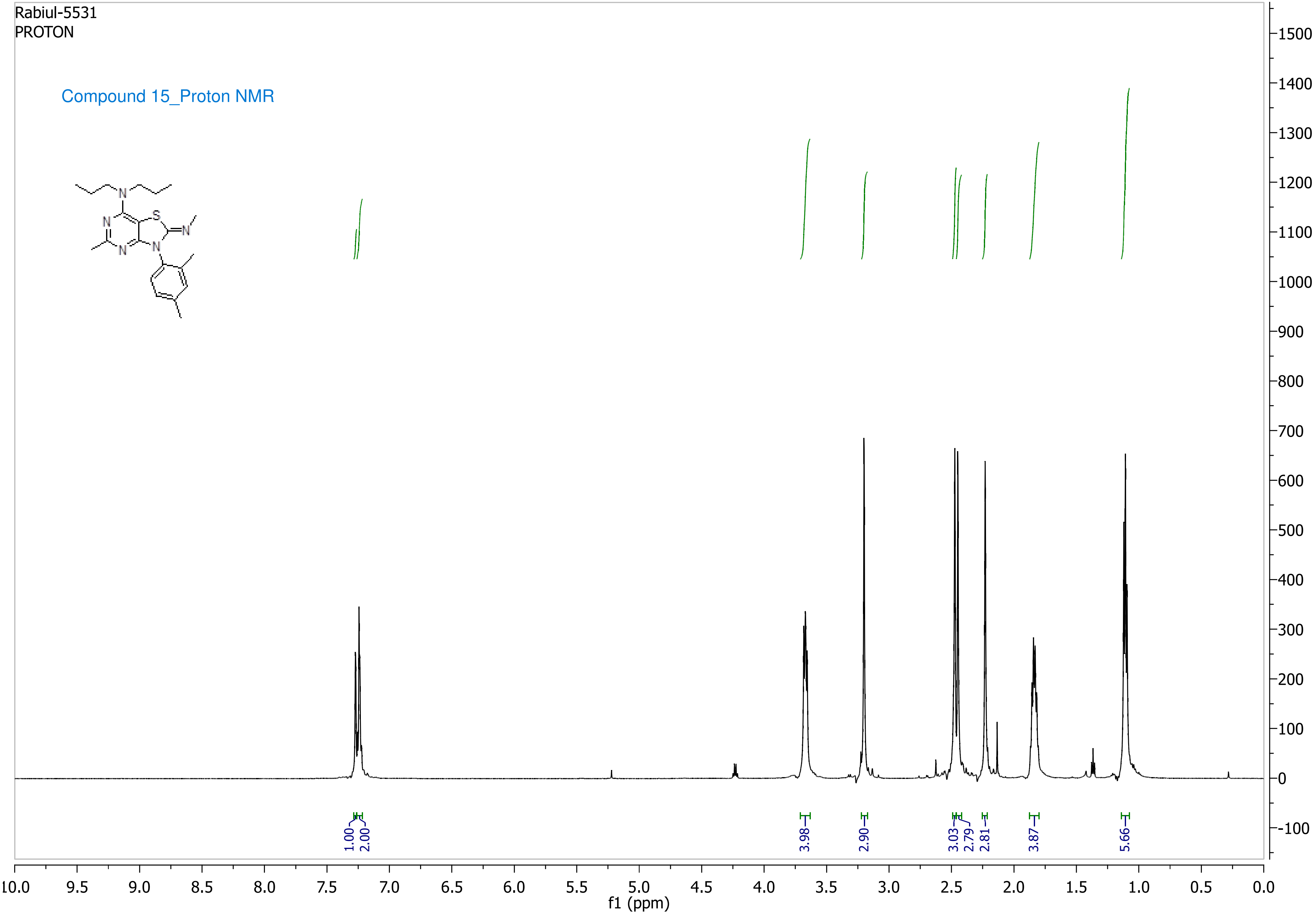
Rabiul-5531
PROTON
Compound 15_Proton NMR
1.00
2.00
3.98
2.90
3.03
2.79
2.81
3.87
5.66
f1 (ppm)

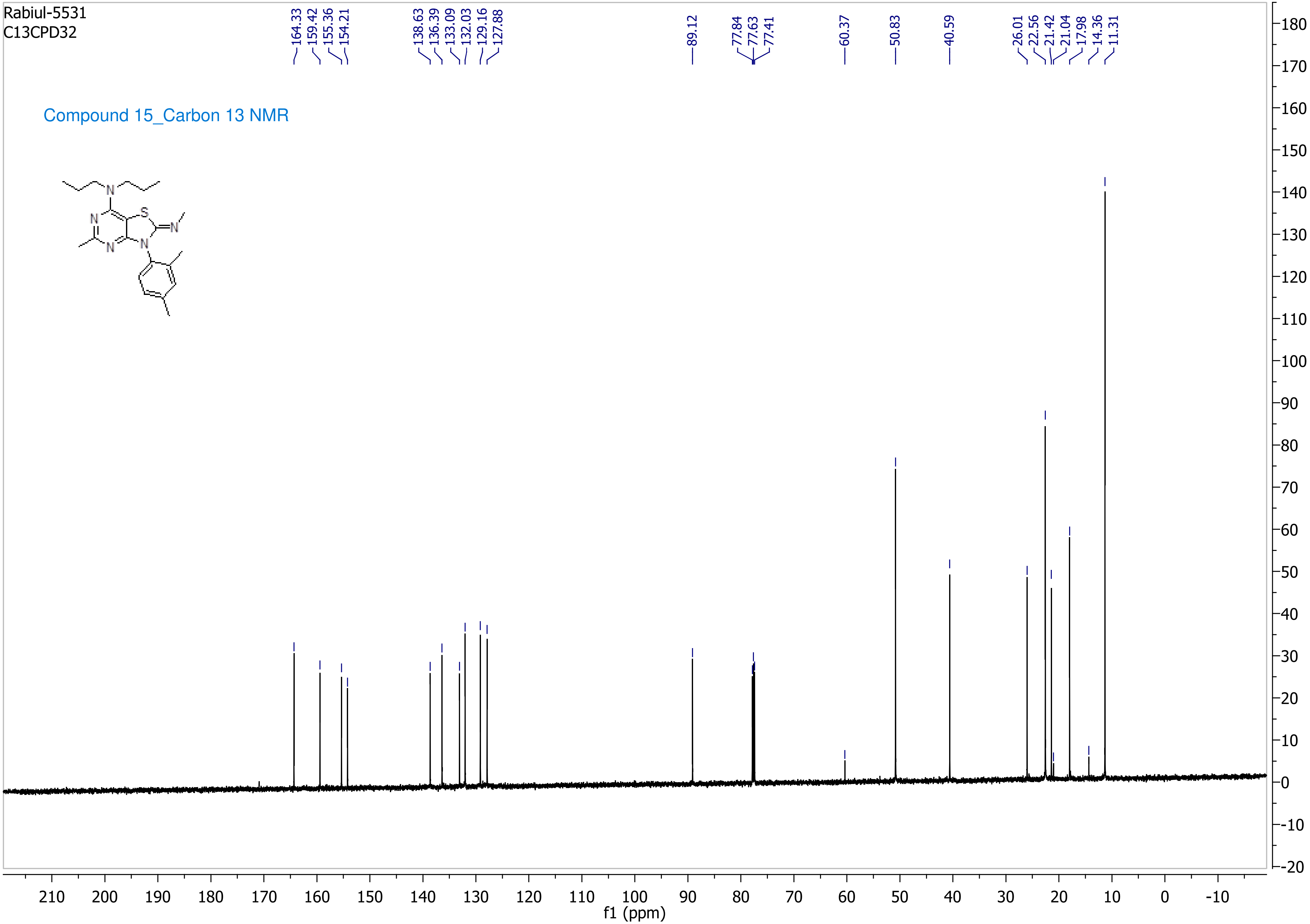
Rabiul-5531
C13CPD32
Compound 15_Carbon 13 NMR
164.33
159.42
155.36
154.21
138.63
136.39
133.09
132.03
129.16
127.88
89.12
77.84
77.63
77.41
60.37
50.83
40.59
26.01
22.56
21.42
21.04
17.98
14.36
11.31
f1 (ppm)

UB Mass Spectrometry Facility - SmartFormula Report

Analysis Info

Analysis Name	D:\Data\IC\20191112\South Dakota\5531_000001.d	Acquisition Date	11/12/2019 2:39:57 PM
Method	Bruker_11052015	Operator	
Sample Name	5531	Instrument	solariX
Comment			

Acquisition Parameters

Acquisition Mode	Single MS	Nebulizer gas flow rate	1.2 bar
n/a	n/a	Drying Gas Temperature	180.0 °C
Broadband Low Mass	147.4 m/z	Drying Gas Flow Rate	4.2 L/min
Broadband High Mass	3000.0 m/z	Calibration Date	Wed Jan 27 11:18:58 2016
Time of Flight to Detector	0.001 sec	Data Acquisition Size	1048576
Ion Accumulation Time	0.100 sec	Data Processing Size (SI)	2097152
Acquired Scans	20	Apodization	Full-Sine
Capillary	4000.0 V		

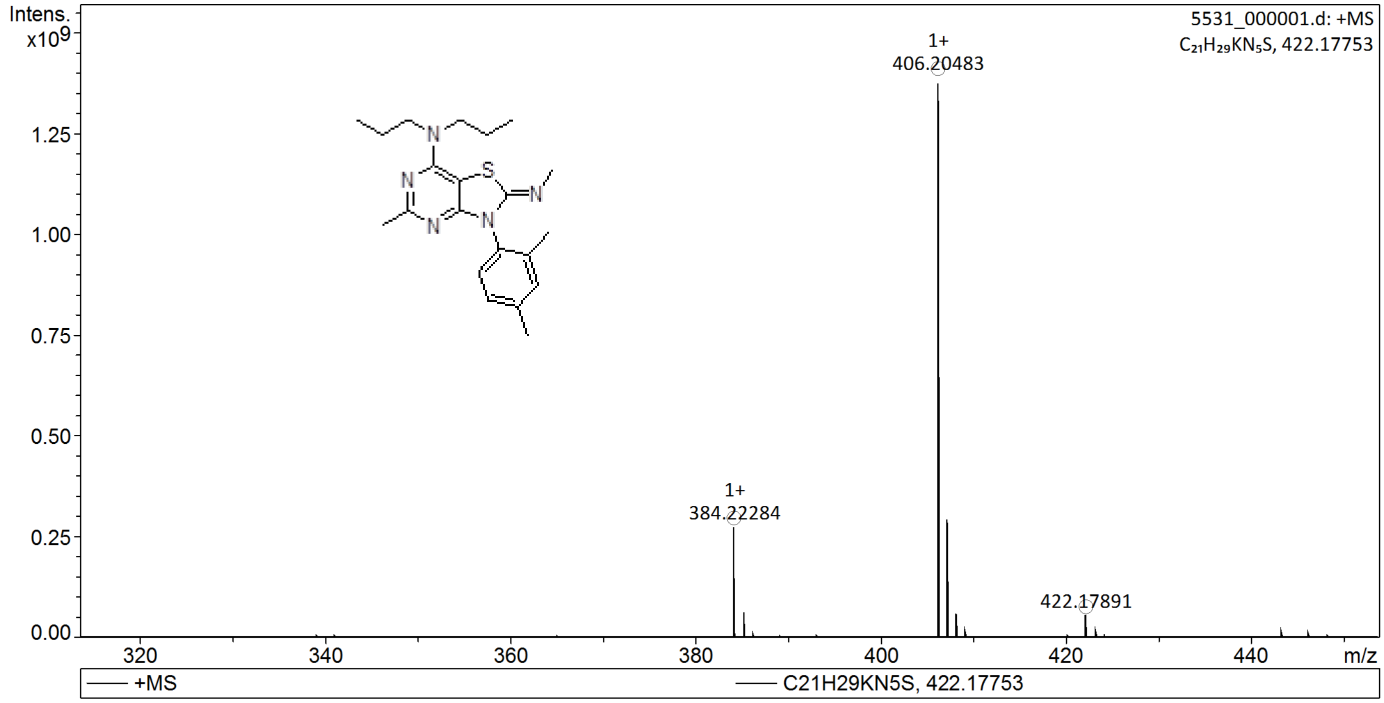

Meas. m/z	#	Ion Formula	Score	m/z	err [ppm]	Mean err [ppm]	mSigma	rdb	e⁻ Conf	N-Rule
384.222841	1	C21H30N5S	100.00	384.221644	-3.1	-2.7	11.6	12.0	even	ok
406.204828	1	C21H29N5NaS	100.00	406.203588	-3.1	-2.7	13.4	12.0	even	ok
422.178908	1	C21H29KN5S	100.00	422.177525	-3.3	-2.7	31.0	12.0	even	ok

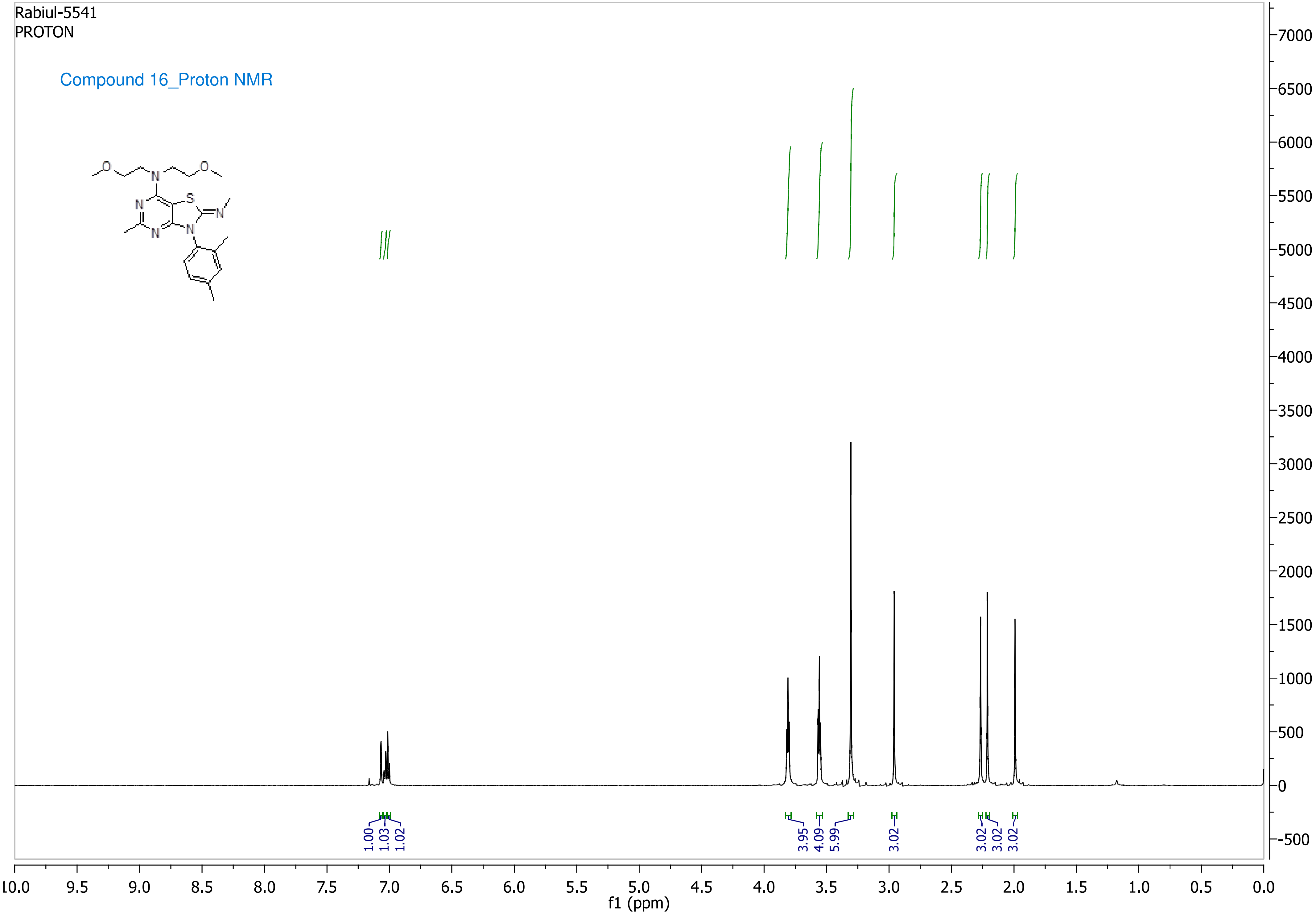

Rabiul-5541
PROTON
Compound 16_Proton NMR
N
S
N
N
N
O
O
1.00
1.03
1.02
3.95
4.09
5.99
3.02
3.02
3.02
3.02
f1 (ppm)

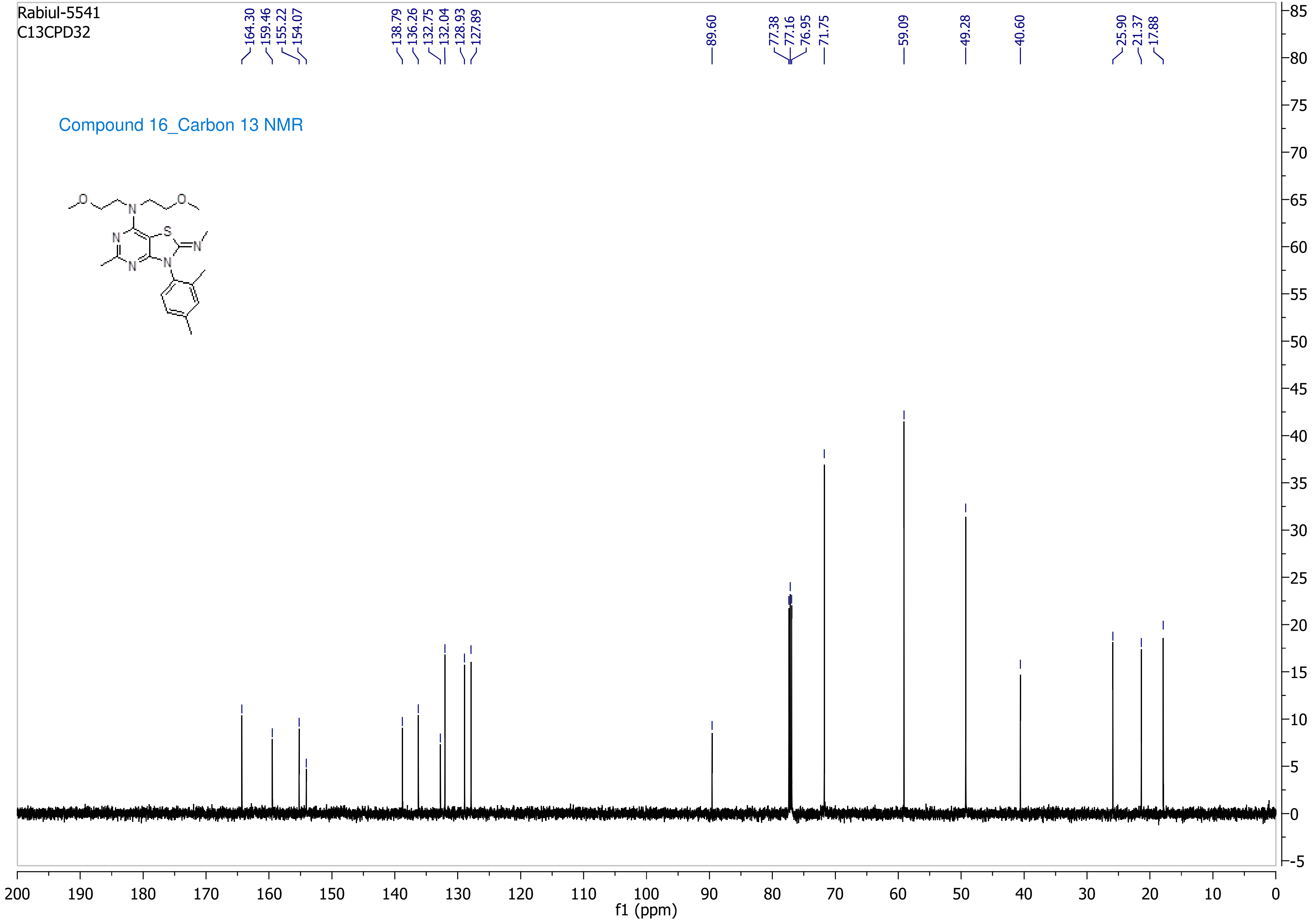
Rabiul-5541
C13CPD32
Compound 16_Carbon 13 NMR
164.30
159.46
155.22
154.07
138.79
136.26
132.75
132.04
128.93
127.89
89.60
77.38
77.16
76.95
71.75
59.09
49.28
40.60
25.90
21.37
17.88
f1 (ppm)

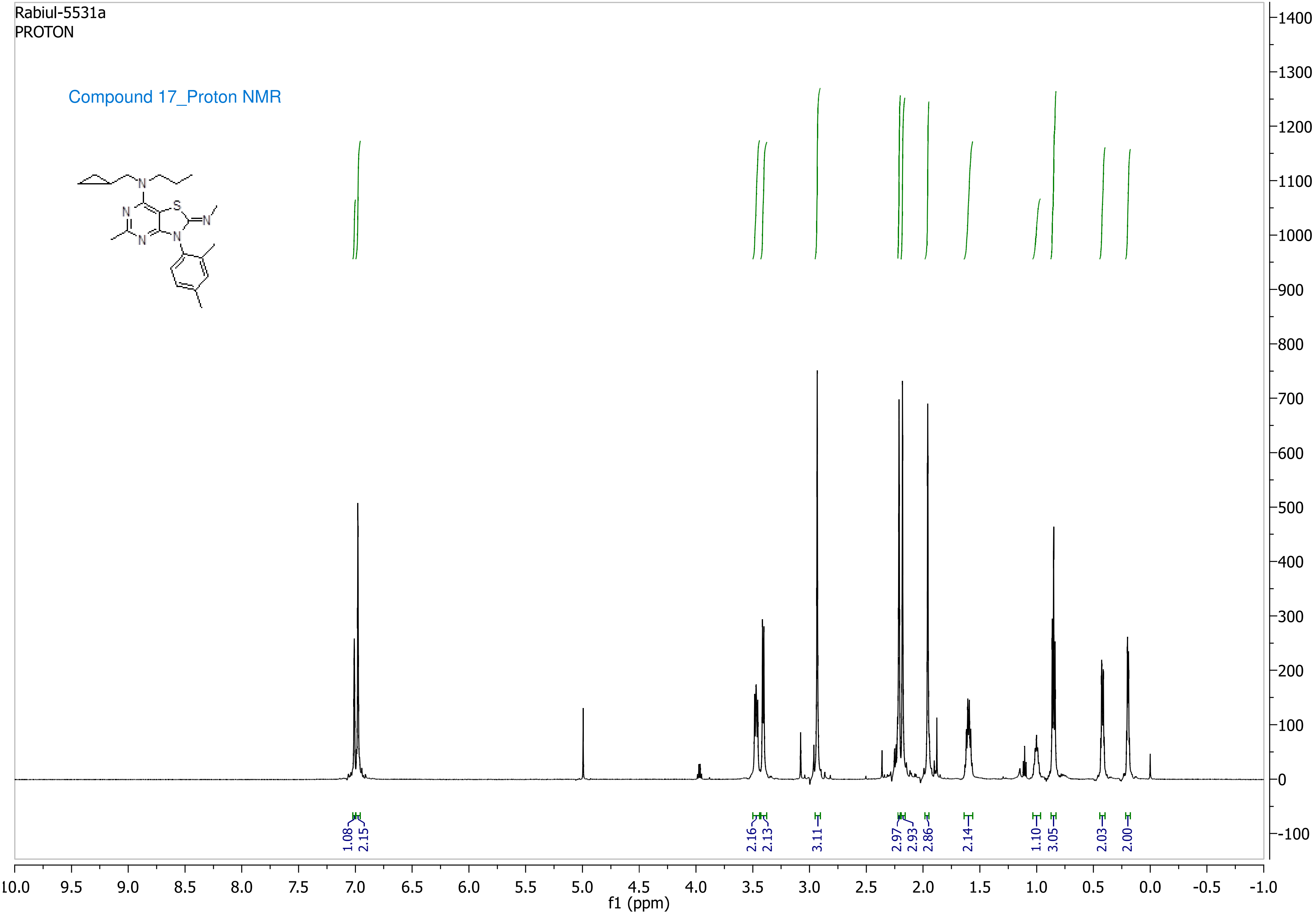

Rabiul-5531a
PROTON
Compound 17_Proton NMR
N
S
N
N
N
N
1.08
2.15
2.16
2.13
3.11
2.97
2.93
2.86
2.14
1.10
3.05
2.03
2.00
10.0
9.5
9.0
8.5
8.0
7.5
7.0
6.5
6.0
5.5
5.0
4.5
4.0
3.5
3.0
2.5
2.0
1.5
1.0
0.5
0.0
-0.5
-1.0
f1 (ppm)
1400
1300
1200
1100
1000
900
800
700
600
500
400
300
200
100
0
-100

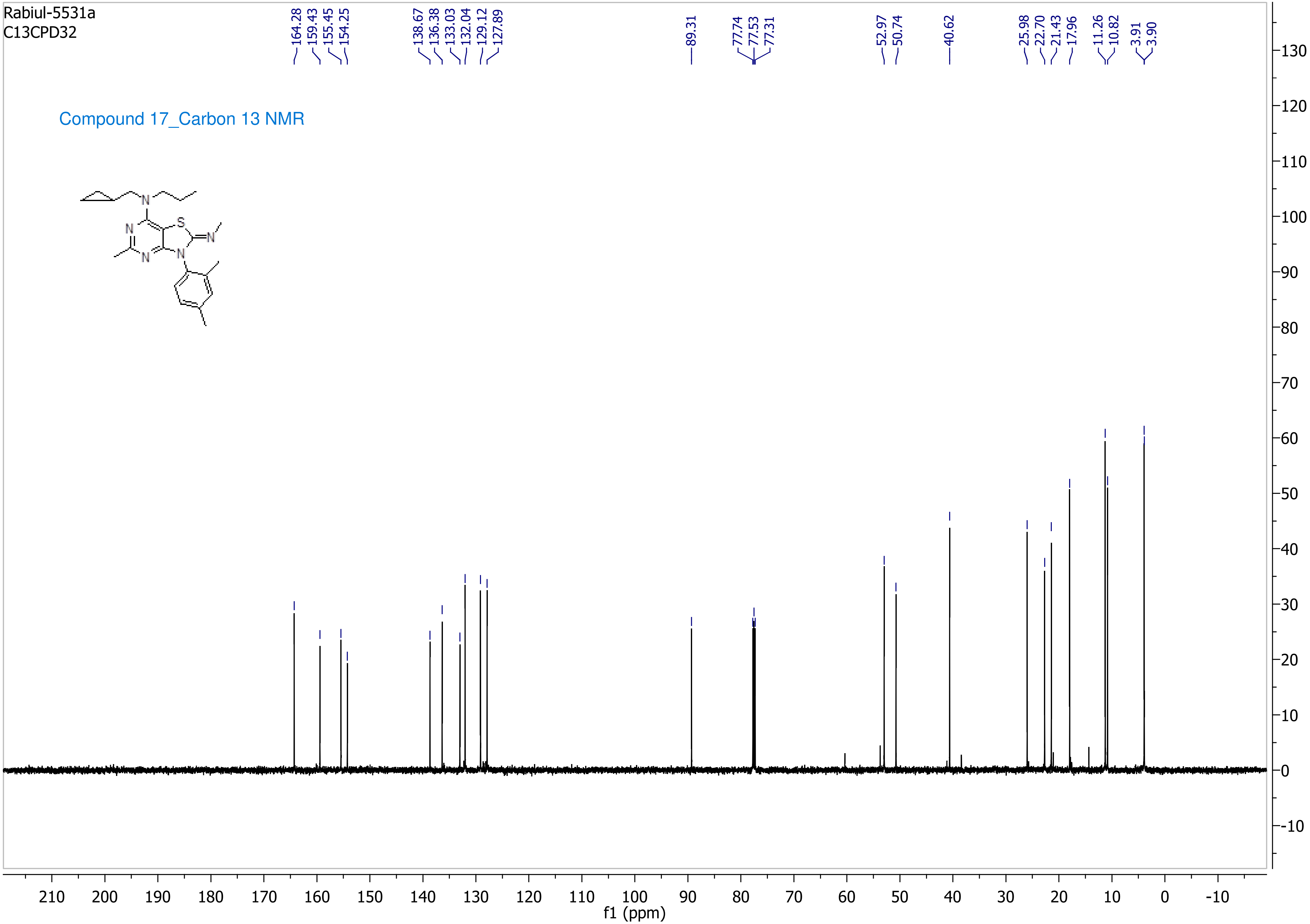
Rabiul-5531a
C13CPD32
Compound 17_Carbon 13 NMR
164.28
159.43
155.45
154.25
138.67
136.38
133.03
132.04
129.12
127.89
89.31
77.74
77.53
77.31
52.97
50.74
40.62
25.98
22.70
21.43
17.96
11.26
10.82
3.91
3.90
f1 (ppm)

UB Mass Spectrometry Facility - SmartFormula Report

Analysis Info

Analysis Name	D:\Data\IC_3-21-19\5531a_000001.d	Acquisition Date	3/21/2019 7:43:27 PM
Method	Bruker_11052015	Operator	
Sample Name	5531a	Instrument	solariX
Comment			

Acquisition Parameters

Acquisition Mode	Single MS	Nebulizer gas flow rate	1.2 bar
Polarity	Positive	Drying Gas Temperature	180.0 °C
Broadband Low Mass	147.4 m/z	Drying Gas Flow Rate	4.2 L/min
Broadband High Mass	1500.0 m/z	Calibration Date	Thu Sep 27 10:56:48 2018
Time of Flight to Detector	0.001 sec	Data Acquisition Size	1048576
Ion Accumulation Time	0.100 sec	Data Processing Size (SI)	2097152
Acquired Scans	20	Apodization	Full-Sine
Capillary	6000.0 V		

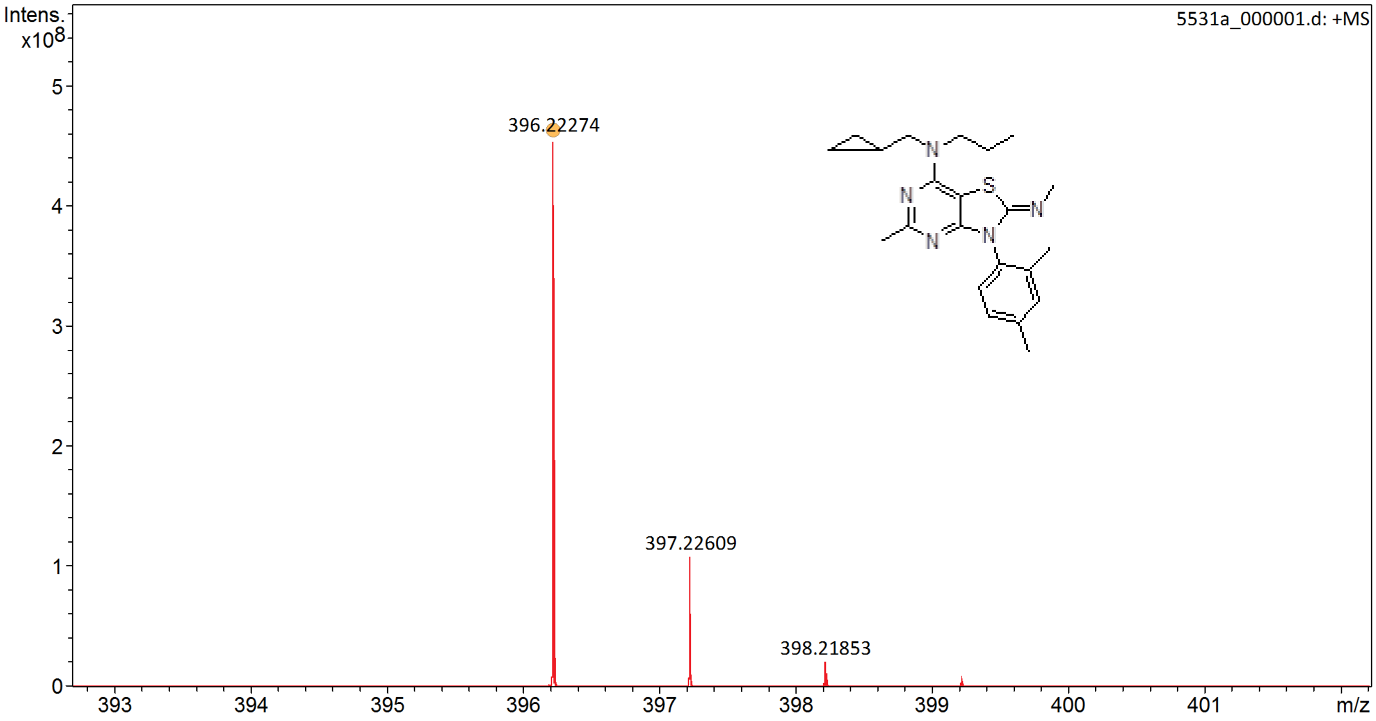

Adduct	Meas. m/z	#	Ion Formula	Score	m/z	err [ppm]	Mean err [ppm]	mSigma	rdb	e⁻ Conf	N-Rule
M+H	396.222739	1	C22H30N5S	100.00	396.221643	-2.8	-2.7	2.1	10.5	even	ok
M+Na	418.204475	1	C22H29N5NaS	100.00	418.203588	-2.1	-2.1	3.7	10.5	even	ok

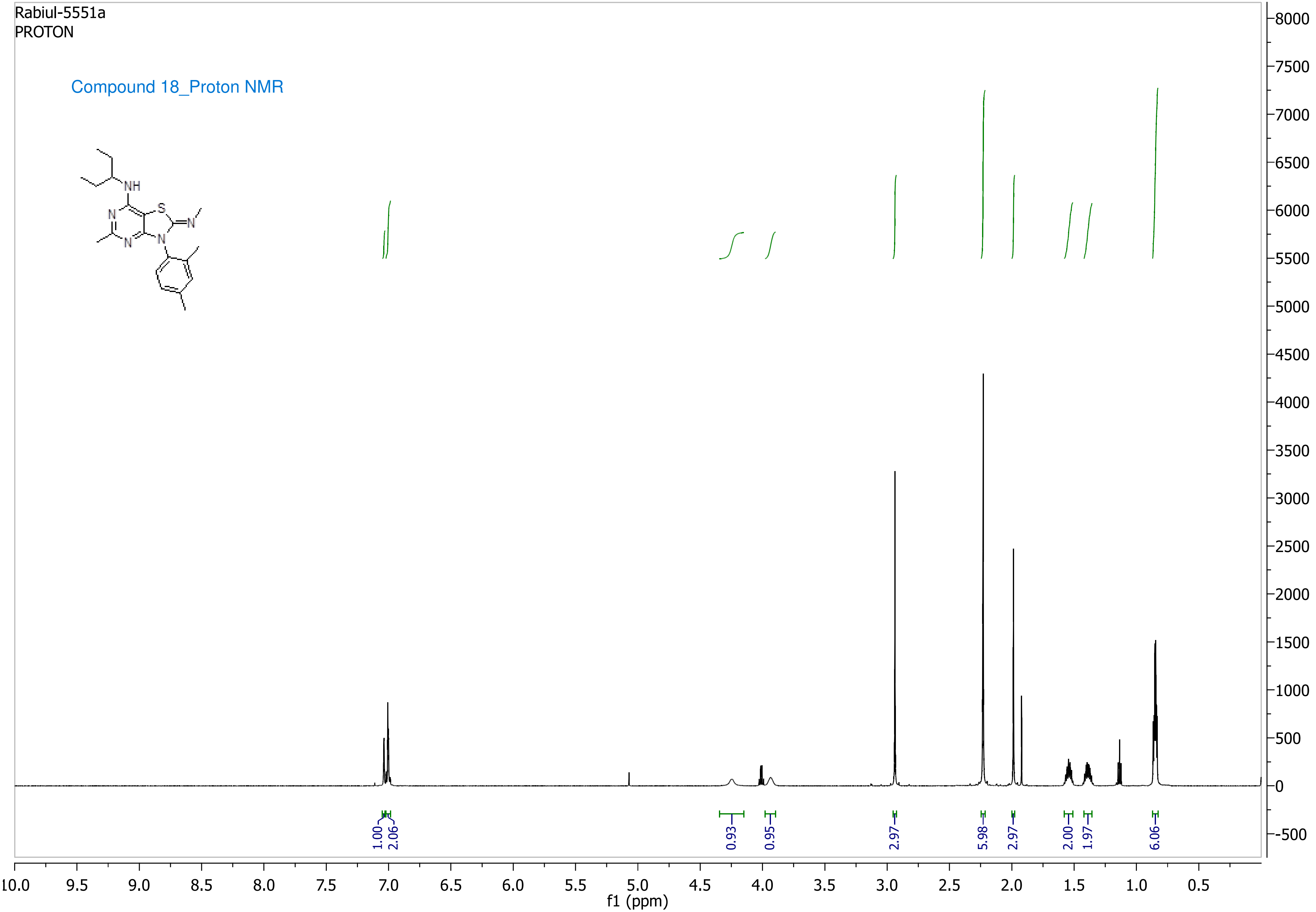
Rabiul-5551a
PROTON
Compound 18_Proton NMR
NH
S
N
N
N
N
1.00
2.06
0.93
0.95
2.97
5.98
2.97
2.00
1.97
6.06
f1 (ppm)

Compound 18_MS

UB Mass Spectrometry Facility - SmartFormula Report

Analysis Info

Analysis Name	D:\Data\IC_3-21-19\5551a_000001.d	Acquisition Date	3/21/2019 7:40:43 PM
Method	Bruker_11052015	Operator	
Sample Name	5551a	Instrument	solariX
Comment			

Acquisition Parameters

Acquisition Mode	Single MS	Nebulizer gas flow rate	1.2 bar
Polarity	Positive	Drying Gas Temperature	180.0 °C
Broadband Low Mass	147.4 m/z	Drying Gas Flow Rate	4.2 L/min
Broadband High Mass	1500.0 m/z	Calibration Date	Thu Sep 27 10:56:48 2018
Time of Flight to Detector	0.001 sec	Data Acquisition Size	1048576
Ion Accumulation Time	0.100 sec	Data Processing Size (SI)	2097152
Acquired Scans	20	Apodization	Full-Sine
Capillary	6000.0 V		

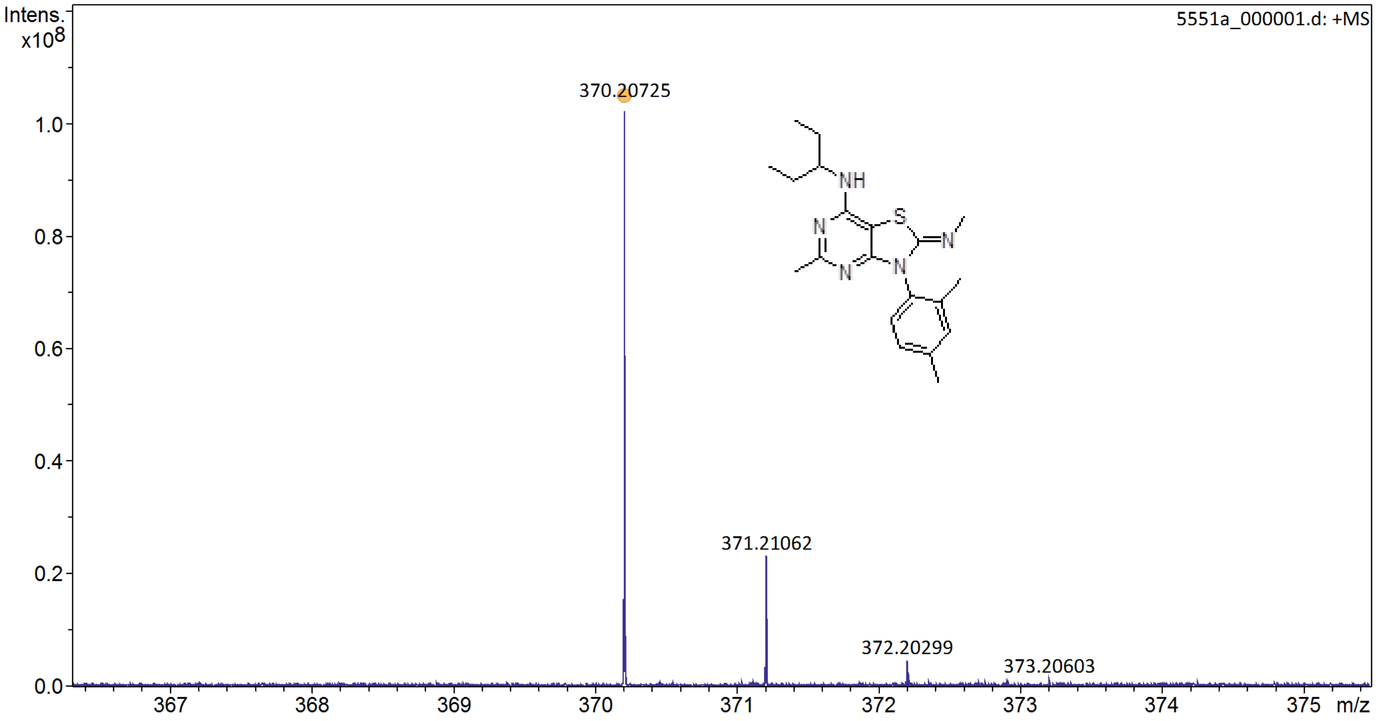

Adduct	Meas. m/z	#	Ion Formula	Score	m/z	err [ppm]	Mean err [ppm]	mSigma	rdb	e¯ Conf	N-Rule
M+H	370.207246	1	C20H28N5S	100.00	370.205993	-3.4	-3.5	4.7	9.5	even	ok
M+Na	392.189038	1	C20H27N5NaS	100.00	392.187938	-2.8	431.3	13.1	9.5	even	ok

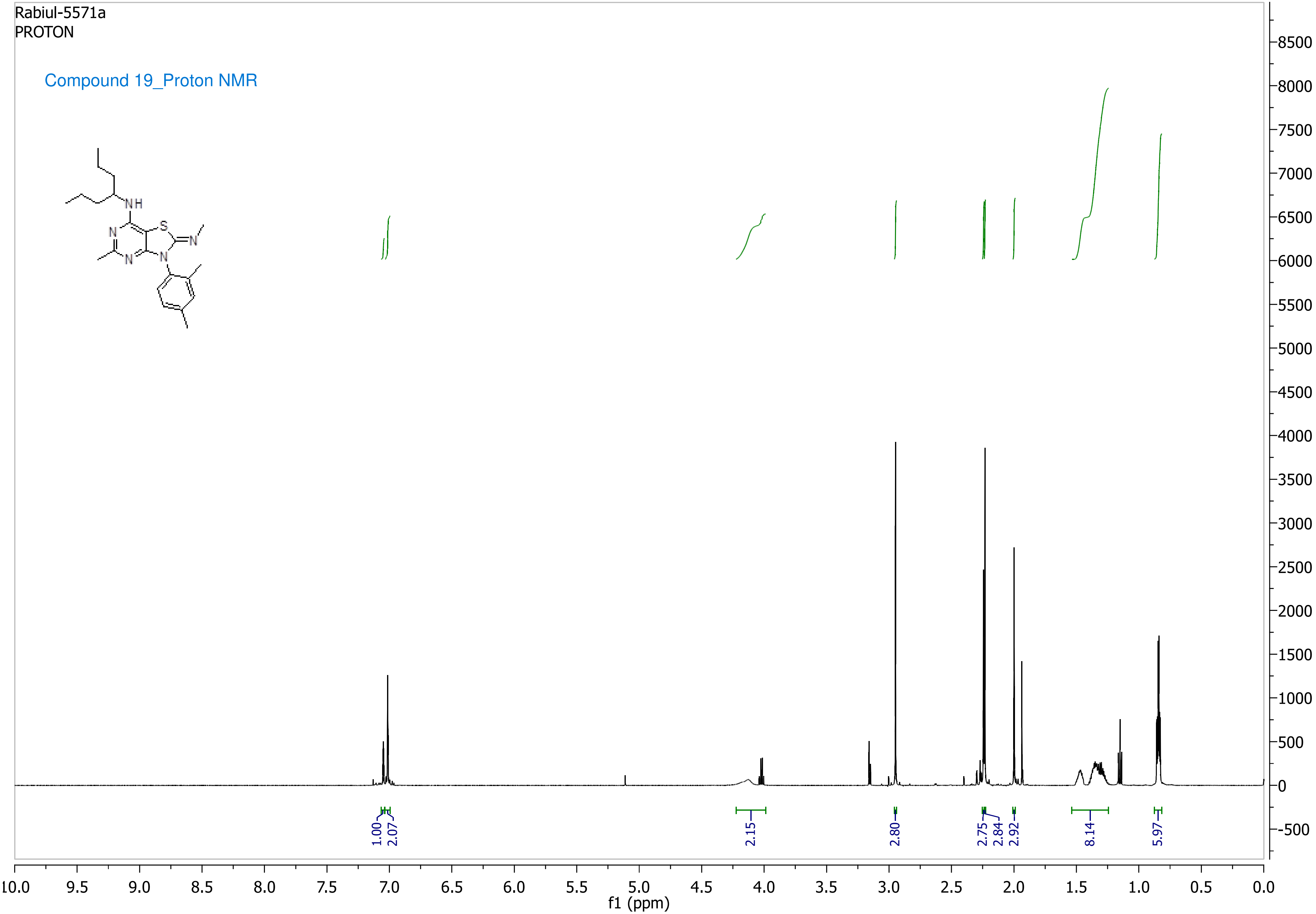
Rabiul-5571a
PROTON
Compound 19_Proton NMR
NH
N
S
N
N
N
1.00
2.07
2.15
2.80
2.75
2.84
2.92
8.14
5.97
10.0
9.5
9.0
8.5
8.0
7.5
7.0
6.5
6.0
5.5
5.0
4.5
4.0
3.5
3.0
2.5
2.0
1.5
1.0
0.5
0.0
f1 (ppm)
8500
8000
7500
7000
6500
6000
5500
5000
4500
4000
3500
3000
2500
2000
1500
1000
500
0
-500

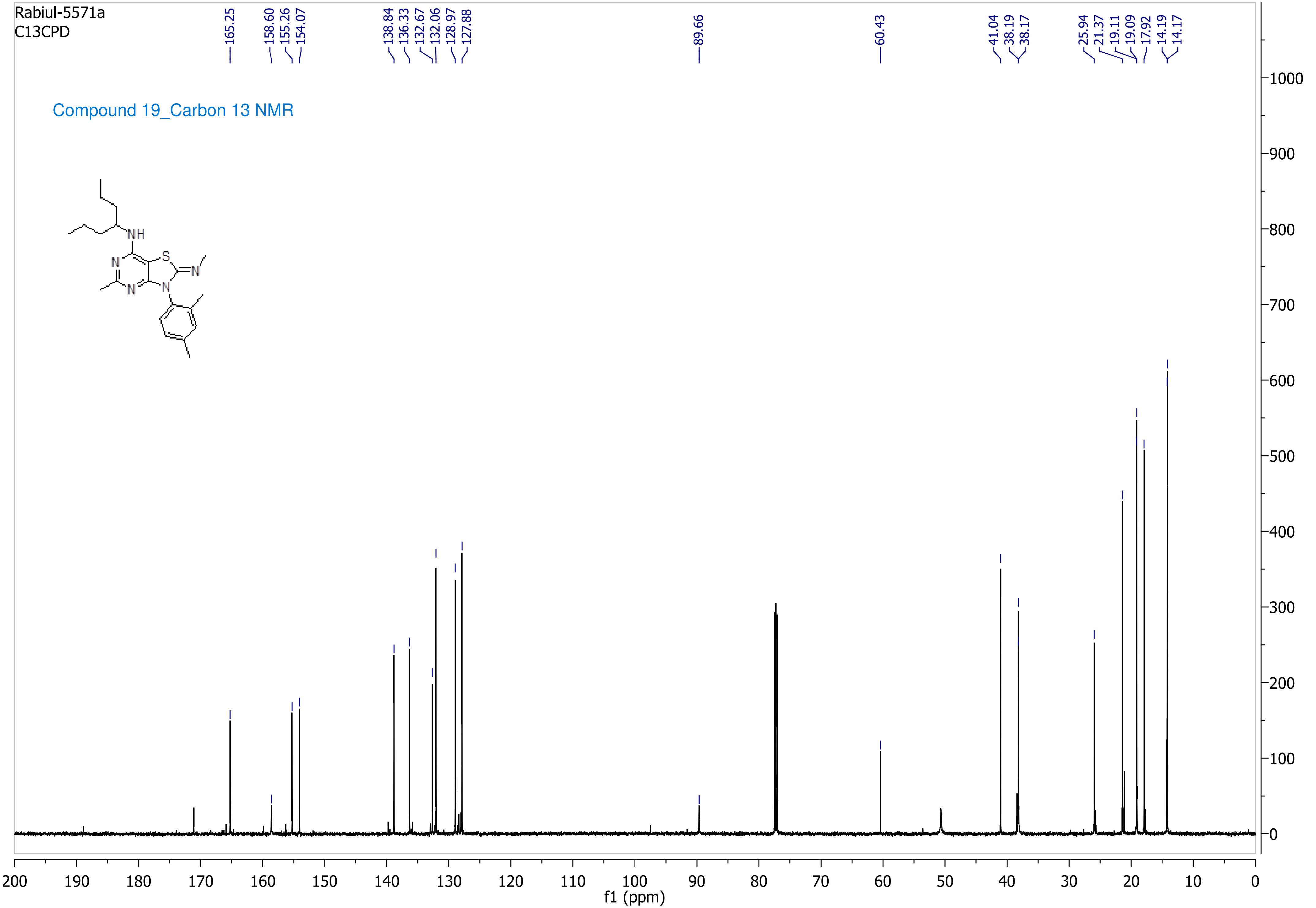

Rabiul-5571a
C13CPD
Compound 19_Carbon 13 NMR
165.25
158.60
155.26
154.07
138.84
136.33
132.67
132.06
128.97
127.88
89.66
60.43
41.04
38.19
38.17
25.94
21.37
19.11
19.09
17.92
14.19
14.17
NH
N
S
N
N
N
f1 (ppm)

Compound 19_MS

UB Mass Spectrometry Facility - SmartFormula Report

Analysis Info

Analysis Name	D:\Data\IC_3-21-19\5571a_000001.d	Acquisition Date	3/21/2019 7:34:51 PM
Method	Bruker_11052015	Operator	
Sample Name	5571a	Instrument	solariX
Comment			

Acquisition Parameters

Acquisition Mode	Single MS	Nebulizer gas flow rate	1.2 bar
Polarity	Positive	Drying Gas Temperature	180.0 °C
Broadband Low Mass	147.4 m/z	Drying Gas Flow Rate	4.2 L/min
Broadband High Mass	1500.0 m/z	Calibration Date	Thu Sep 27 10:56:48 2018
Time of Flight to Detector	0.001 sec	Data Acquisition Size	1048576
Ion Accumulation Time	0.100 sec	Data Processing Size (SI)	2097152
Acquired Scans	20	Apodization	Full-Sine
Capillary	6000.0 V		

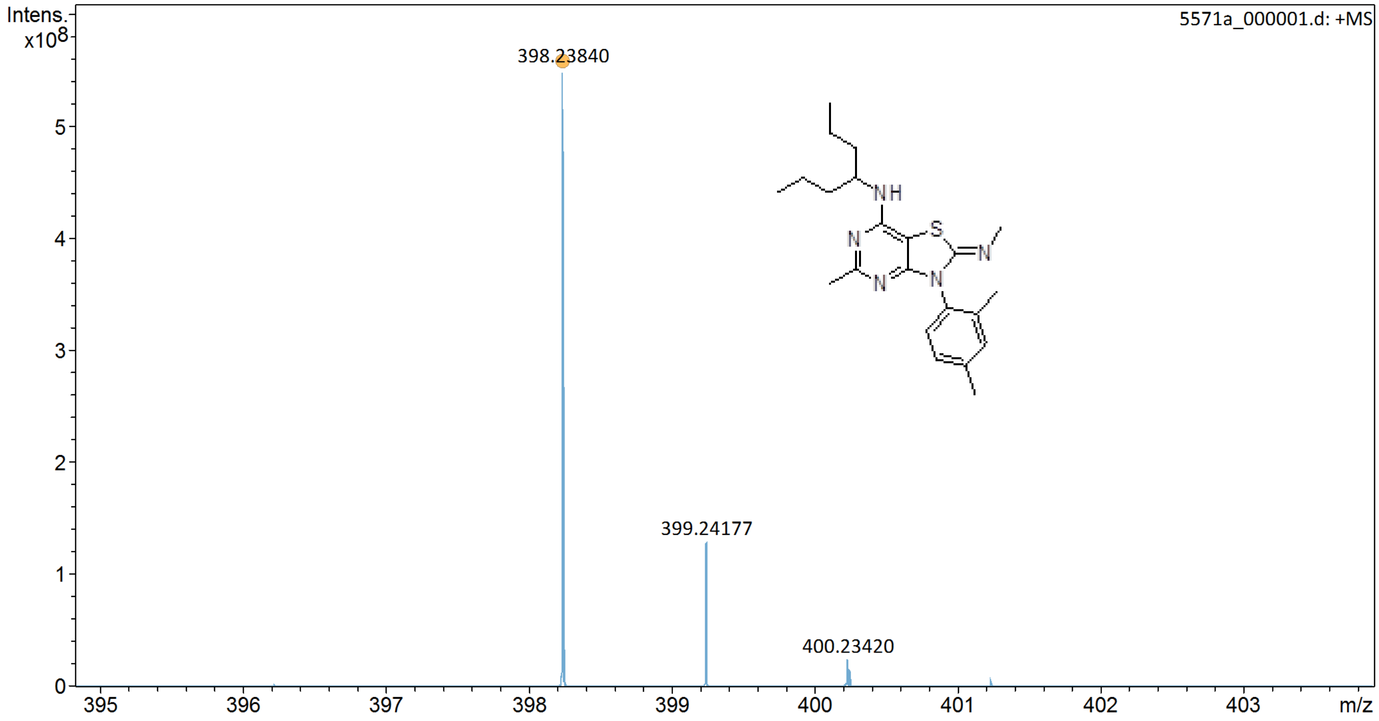

Adduct	Meas. m/z	#	Ion Formula	Score	m/z	err [ppm]	Mean err [ppm]	mSigma	rdb	e⁻ Conf	N-Rule
M+H	398.238398	1	C22H32N5S	100.00	398.237293	-2.8	1274.1	14.1	9.5	even	ok
M+Na	420.220155	1	C22H31N5NaS	100.00	420.219238	-2.2	1207.9	14.9	9.5	even	ok

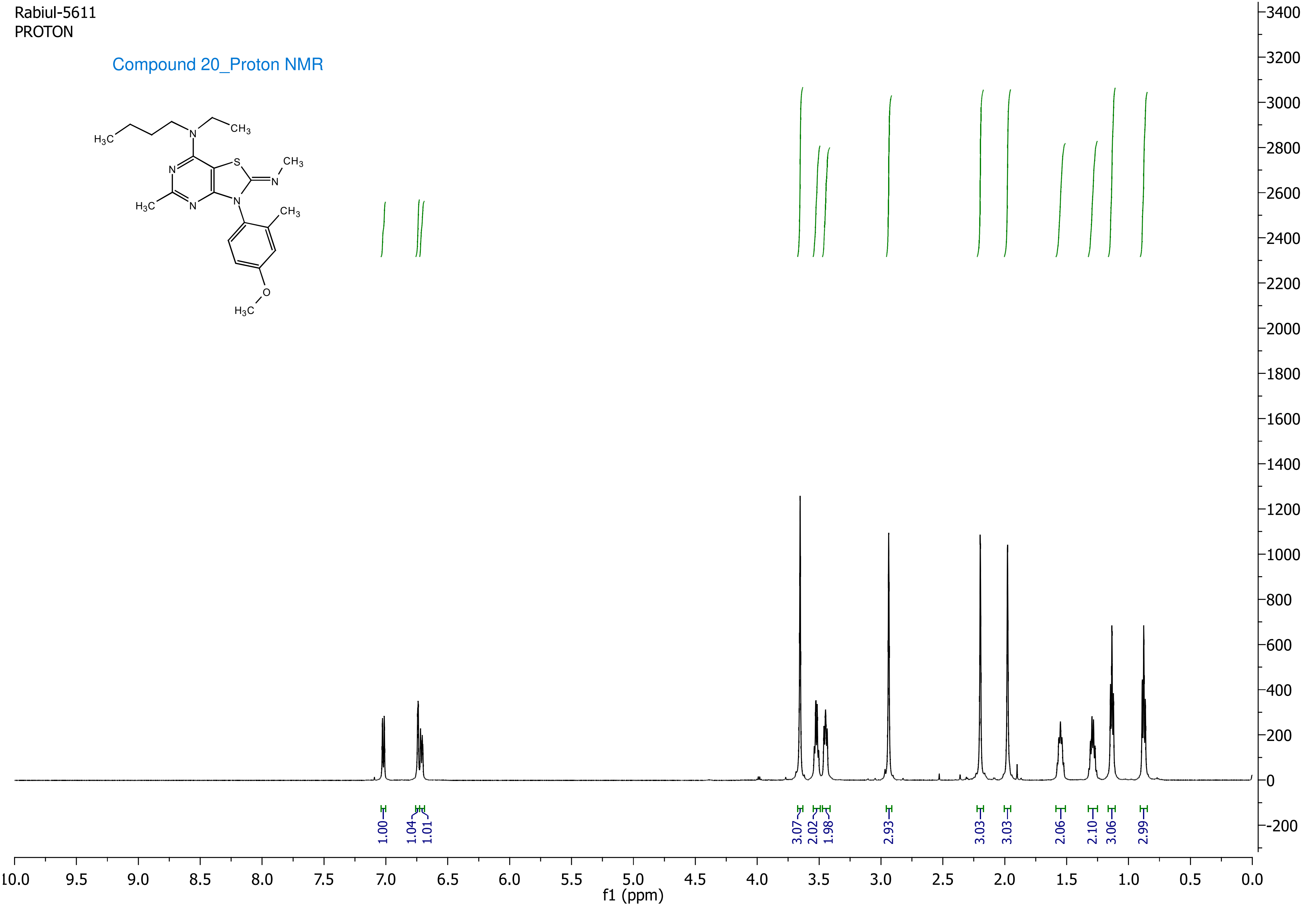
Rabiul-5611
PROTON
Compound 20_Proton NMR
H3C
N
CH3
N
S
CH3
N
H3C
N
N
CH3
O
H3C
1.00
1.04
1.01
3.07
2.02
1.98
2.93
3.03
3.03
2.06
2.10
3.06
2.99
10.0 9.5 9.0 8.5 8.0 7.5 7.0 6.5 6.0 5.5 5.0 4.5 4.0 3.5 3.0 2.5 2.0 1.5 1.0 0.5 0.0
f1 (ppm)
3400 3200 3000 2800 2600 2400 2200 2000 1800 1600 1400 1200 1000 800 600 400 200 0 -200

Rabiul-5611
C13CPD32

Compound 20_Carbon 13 NMR

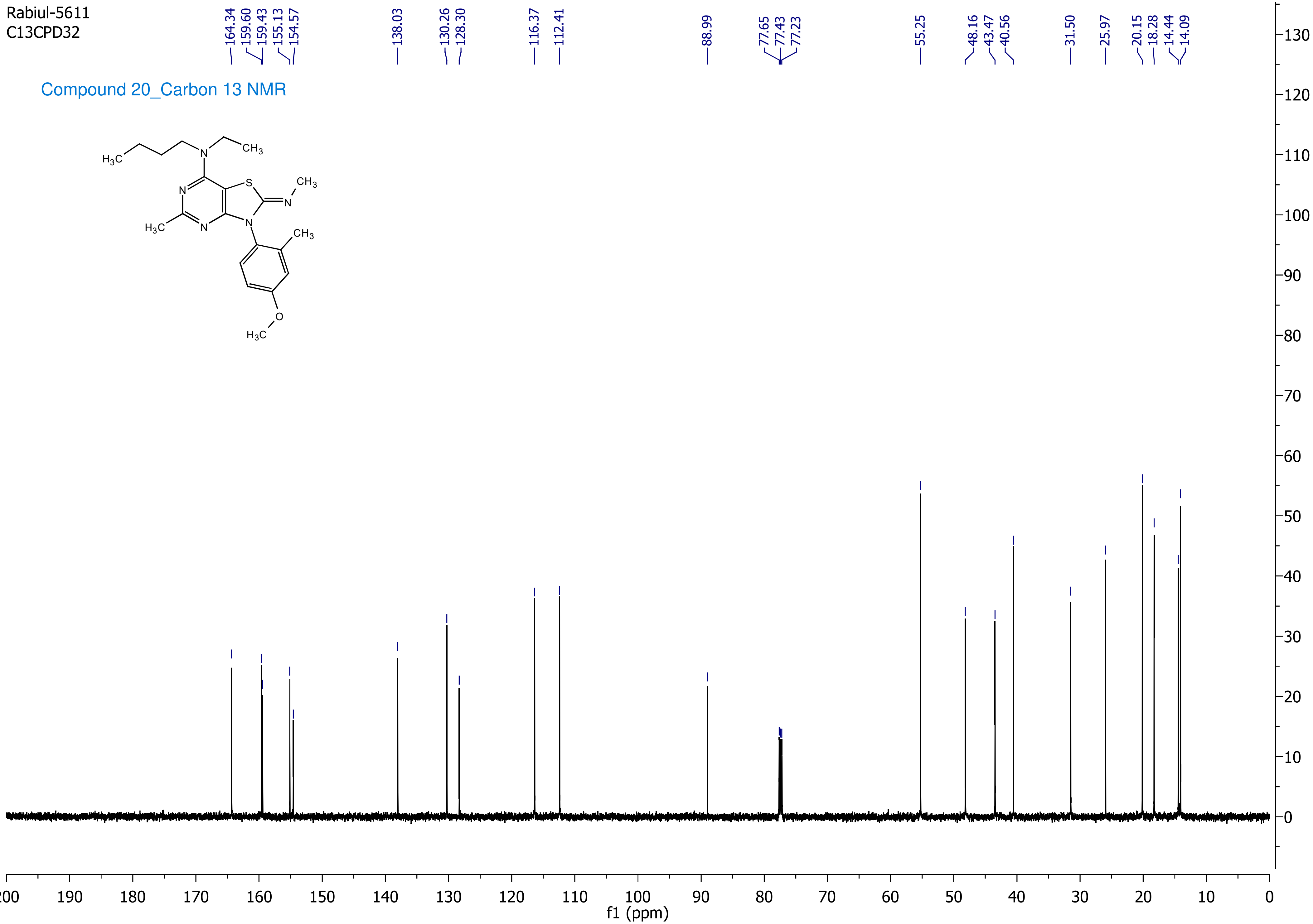

UB Mass Spectrometry Facility - SmartFormula Report

Analysis Info

Analysis Name	D:\Data\IC_3-21-19\5611_000001.d	Acquisition Date	3/21/2019 7:29:44 PM
Method	Bruker_11052015	Operator	
Sample Name	5611	Instrument	solariX
Comment			

Acquisition Parameters

Acquisition Mode	Single MS	Nebulizer gas flow rate	1.2 bar
Polarity	Positive	Drying Gas Temperature	180.0 °C
Broadband Low Mass	147.4 m/z	Drying Gas Flow Rate	4.2 L/min
Broadband High Mass	1500.0 m/z	Calibration Date	Thu Sep 27 10:56:48 2018
Time of Flight to Detector	0.001 sec	Data Acquisition Size	1048576
Ion Accumulation Time	0.100 sec	Data Processing Size (SI)	2097152
Acquired Scans	20	Apodization	Full-Sine
Capillary	6000.0 V		

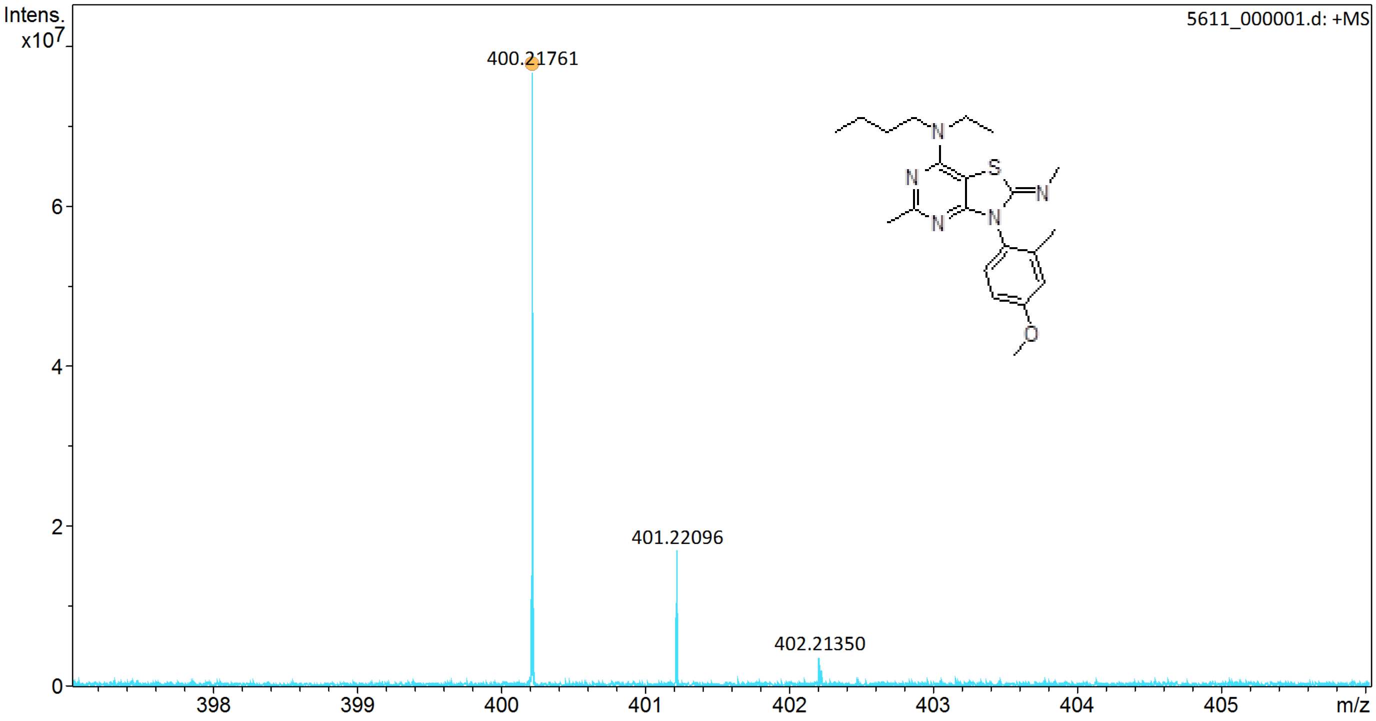

Adduct	Meas. m/z	#	Ion Formula	Score	m/z	err [ppm]	Mean err [ppm]	mSigma	rdb	e⁻ Conf	N-Rule
M+H	400.217609	1	C21H30N5OS	100.00	400.216558	-2.6	-2.7	4.8	9.5	even	ok
M+Na	422.199350	1	C21H29N5NaOS	100.00	422.198502	-2.0	-2.1	2.9	9.5	even	ok

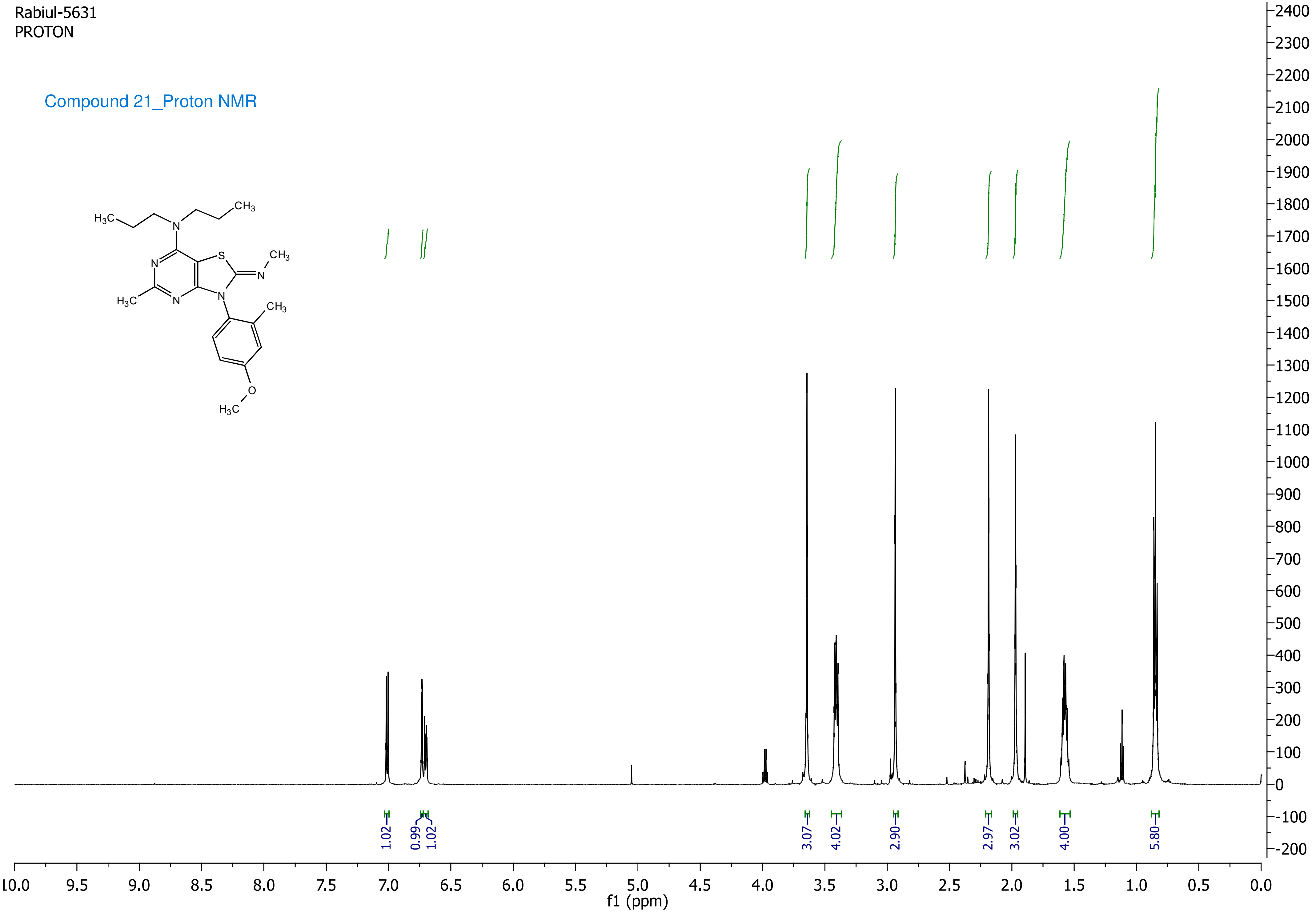

Rabiul-5631
PROTON
Compound 21_Proton NMR
H_3C
CH_3
N
S
CH_3
N
N
N
N
H_3C
CH_3
O
H_3C
1.02
0.99
1.02
3.07
4.02
2.90
2.97
3.02
4.00
5.80
10.0
9.5
9.0
8.5
8.0
7.5
7.0
6.5
6.0
5.5
5.0
4.5
4.0
3.5
3.0
2.5
2.0
1.5
1.0
0.5
0.0
f1 (ppm)
2400
2300
2200
2100
2000
1900
1800
1700
1600
1500
1400
1300
1200
1100
1000
900
800
700
600
500
400
300
200
100
0
-100
-200

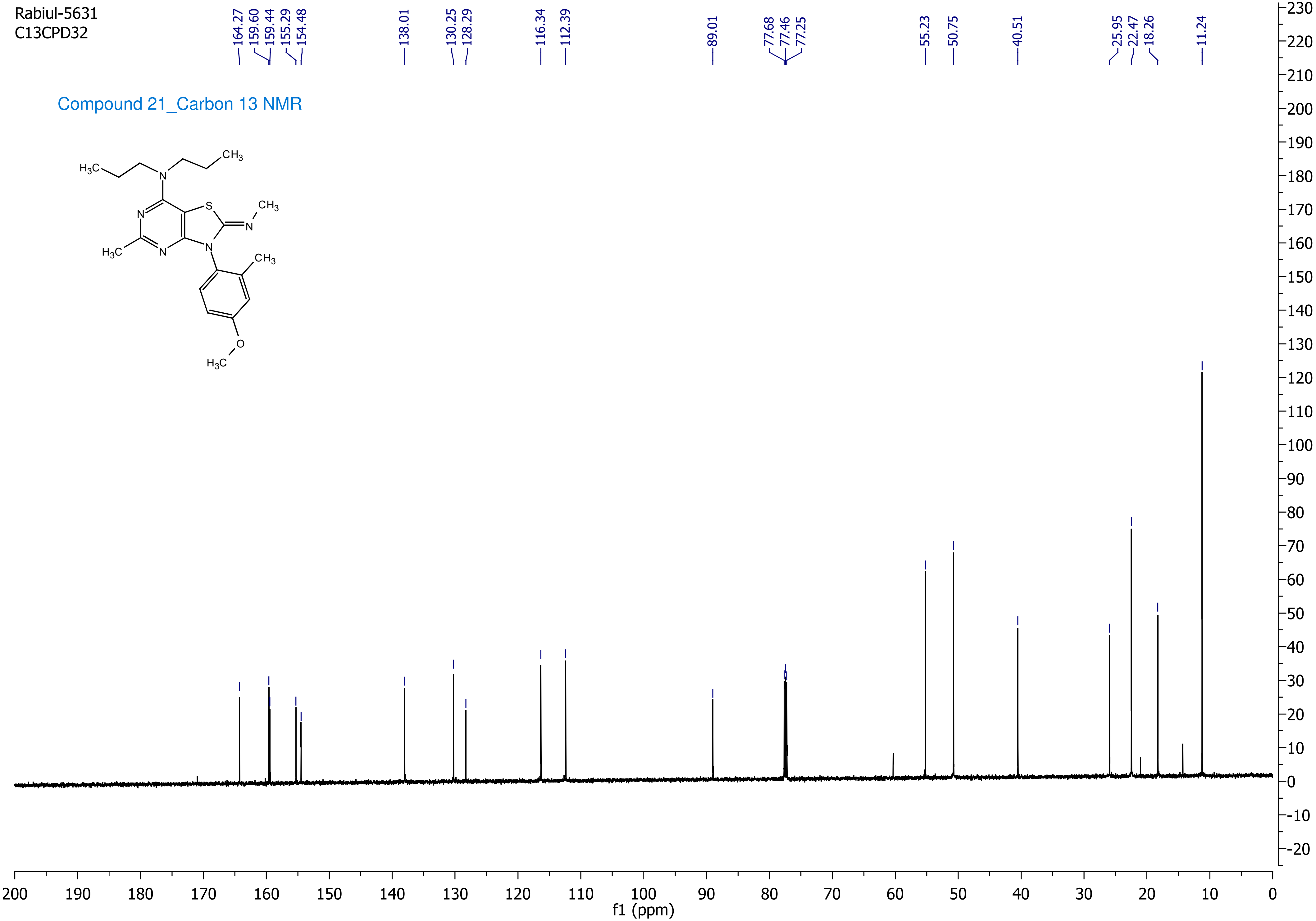

Compound 21_Carbon 13 NMR

UB Mass Spectrometry Facility - SmartFormula Report

Analysis Info

Analysis Name	D:\Data\IC_3-21-19\5631_000001.d	Acquisition Date	3/21/2019 7:24:09 PM
Method	Bruker_11052015	Operator	
Sample Name	5631	Instrument	solariX
Comment			

Acquisition Parameters

Acquisition Mode	Single MS	Nebulizer gas flow rate	1.2 bar
Polarity	Positive	Drying Gas Temperature	180.0 °C
Broadband Low Mass	147.4 m/z	Drying Gas Flow Rate	4.2 L/min
Broadband High Mass	1500.0 m/z	Calibration Date	Thu Sep 27 10:56:48 2018
Time of Flight to Detector	0.001 sec	Data Acquisition Size	1048576
Ion Accumulation Time	0.100 sec	Data Processing Size (SI)	2097152
Acquired Scans	20	Apodization	Full-Sine
Capillary	6000.0 V		

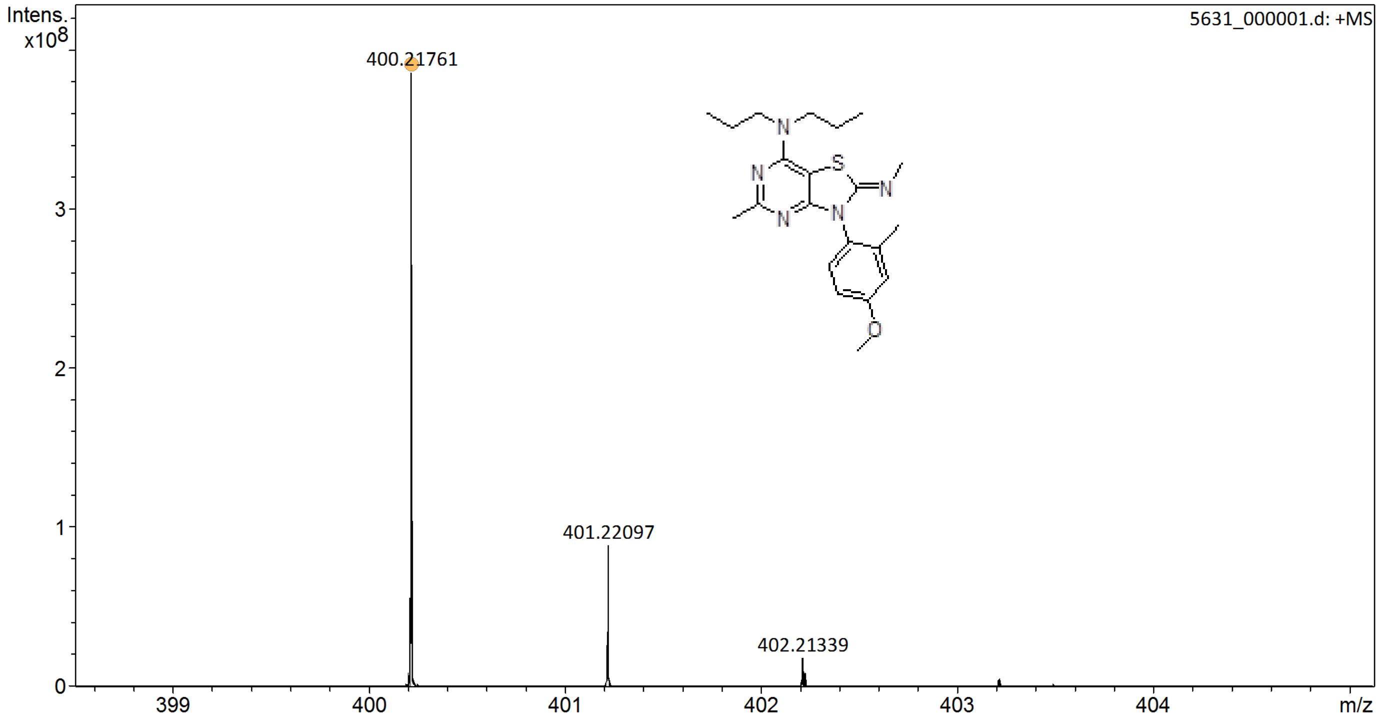

Adduct	Meas. m/z	#	Ion Formula	Score	m/z	err [ppm]	Mean err [ppm]	mSigma	rdb	e⁻ Conf	N-Rule
M+H	400.217611	1	C21H30N5OS	100.00	400.216558	-2.6	-2.7	2.3	9.5	even	ok
M+Na	422.199362	1	C21H29N5NaOS	100.00	422.198502	-2.0	-2.1	2.9	9.5	even	ok

Rabiul-5631a
PROTON

Compound 22_Proton NMR

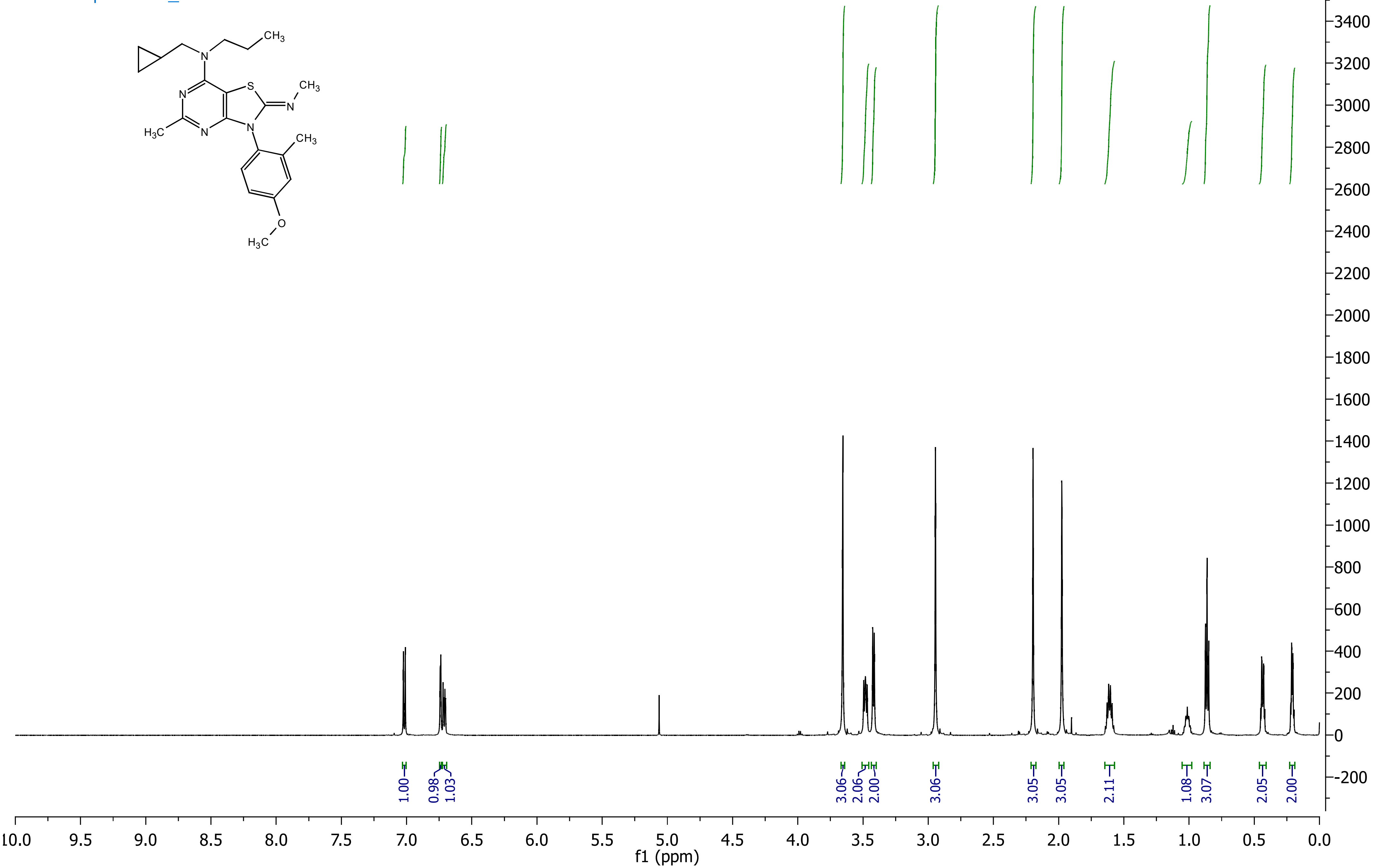

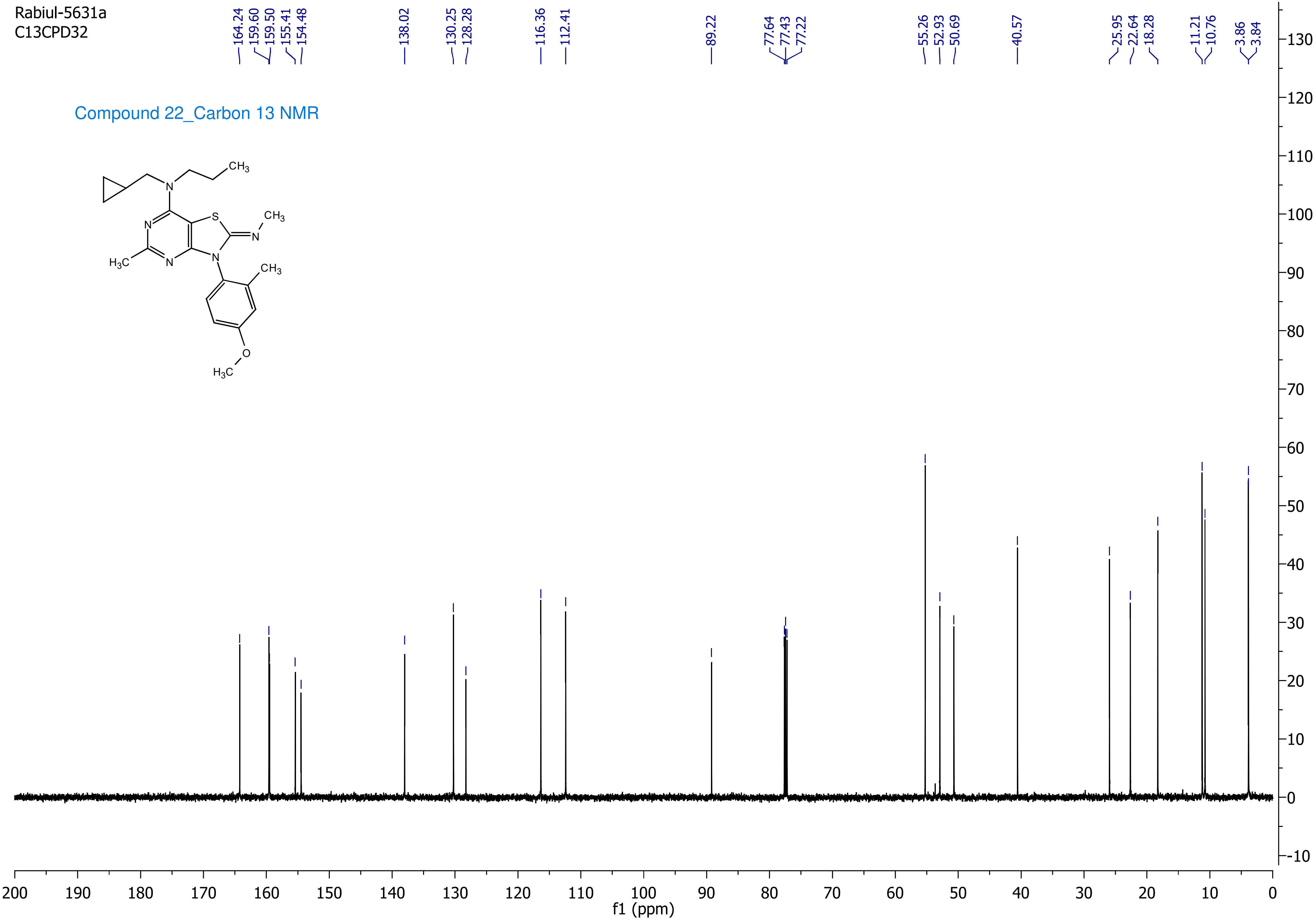
Rabiul-5631a
C13CPD32
Compound 22_Carbon 13 NMR
164.24
159.60
159.50
155.41
154.48
138.02
130.25
128.28
116.36
112.41
89.22
77.64
77.43
77.22
55.26
52.93
50.69
40.57
25.95
22.64
18.28
11.21
10.76
3.86
3.84
f1 (ppm)

Compound 22_MS

UB Mass Spectrometry Facility - SmartFormula Report

Analysis Info

Analysis Name	D:\Data\IC_3-21-19\5631a_000001.d	Acquisition Date	3/21/2019 7:20:02 PM
Method	Bruker_11052015	Operator	
Sample Name	5631a	Instrument	solariX
Comment			

Acquisition Parameters

Acquisition Mode	Single MS	Nebulizer gas flow rate	1.2 bar
Polarity	Positive	Drying Gas Temperature	180.0 °C
Broadband Low Mass	147.4 m/z	Drying Gas Flow Rate	4.2 L/min
Broadband High Mass	1500.0 m/z	Calibration Date	Thu Sep 27 10:56:48 2018
Time of Flight to Detector	0.001 sec	Data Acquisition Size	1048576
Ion Accumulation Time	0.100 sec	Data Processing Size (SI)	2097152
Acquired Scans	20	Apodization	Full-Sine
Capillary	6000.0 V		

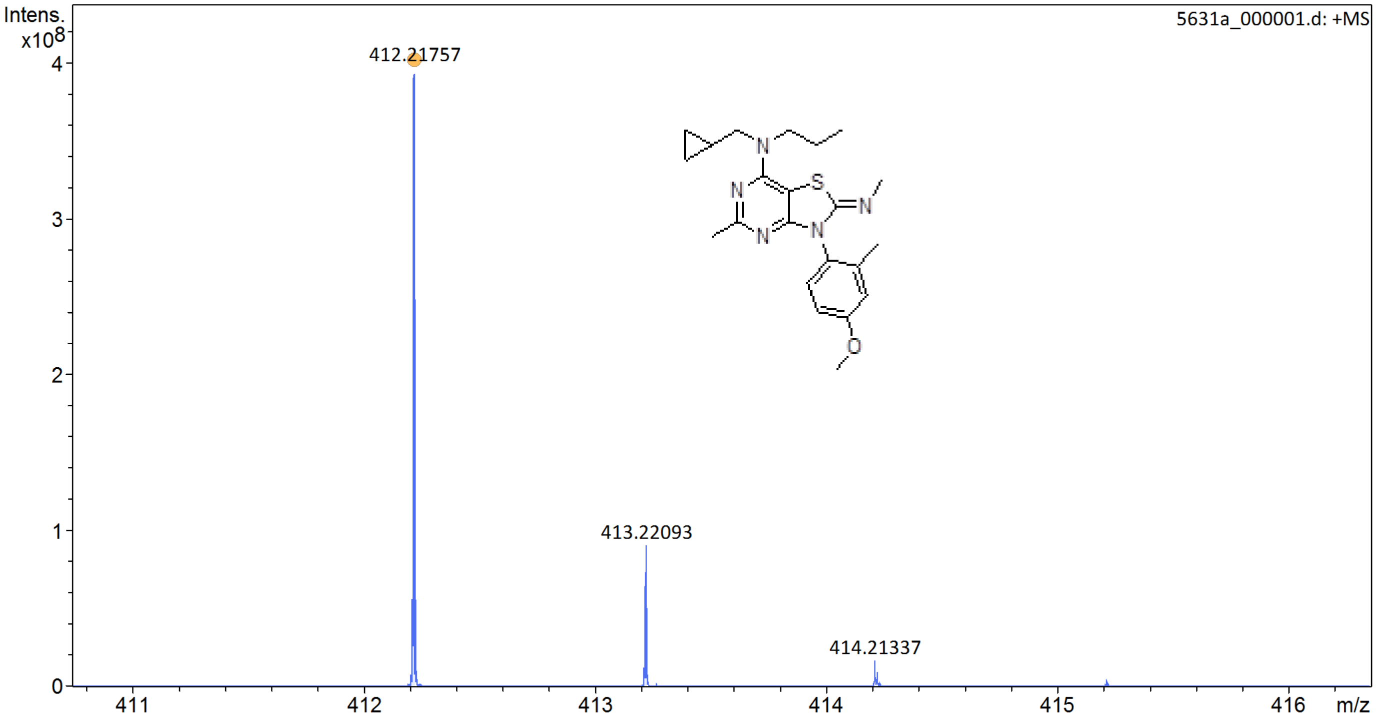

Adduct	Meas. m/z	#	Ion Formula	Score	m/z	err [ppm]	Mean err [ppm]	mSigma	rdb	e¯ Conf	N-Rule
M+H	412.217566	1	C22H30N5OS	100.00	412.216558	-2.4	-2.5	7.1	10.5	even	ok
M+Na	434.199303	1	C22H29N5NaOS	100.00	434.198502	-1.8	-1.9	6.0	10.5	even	ok

Rabiul-5651a
PROTON

Compound 23_Proton NMR

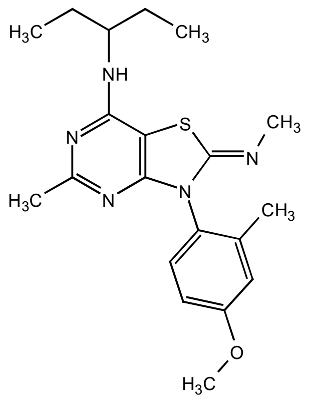

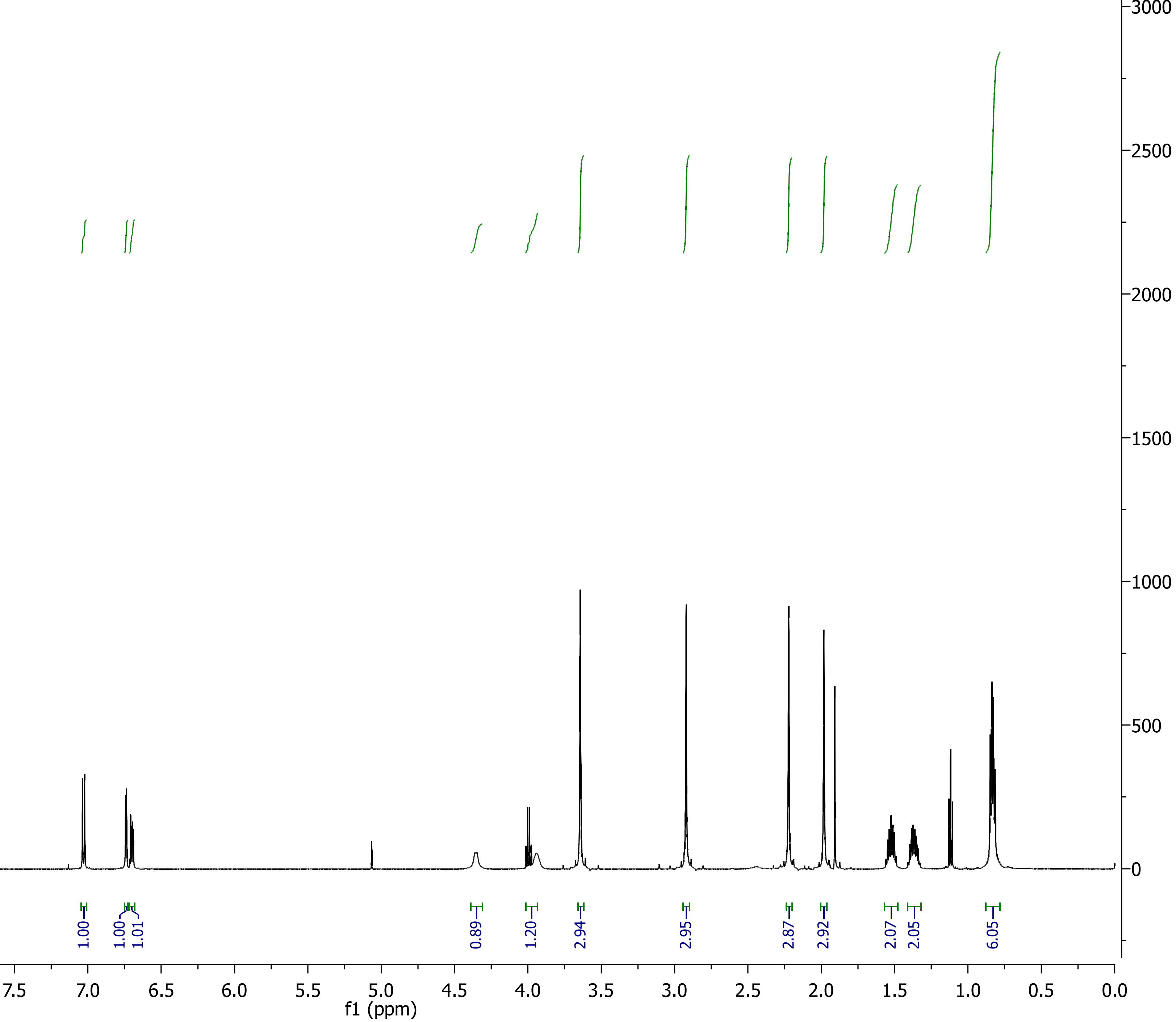

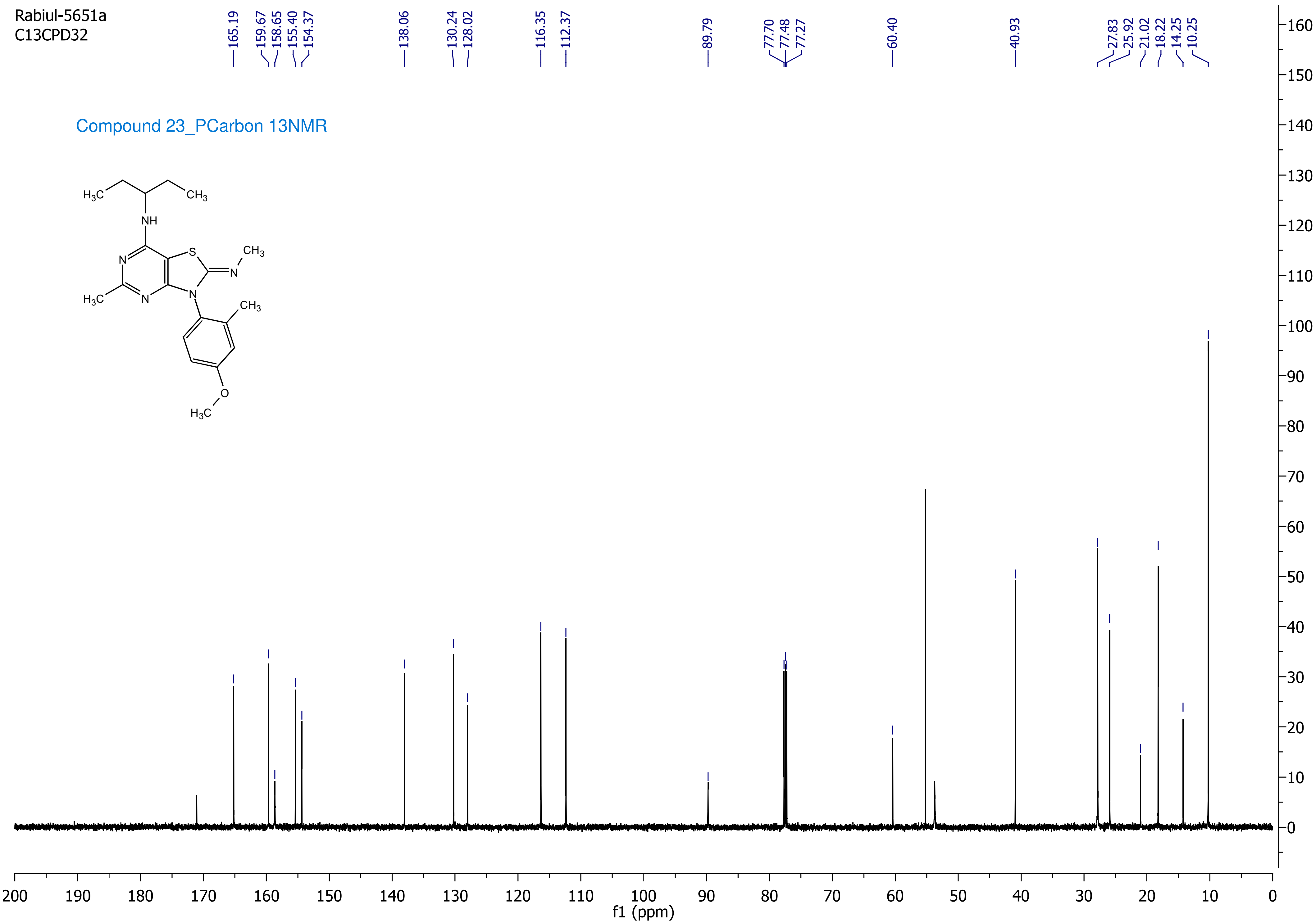
Rabiul-5651a
C13CPD32
Compound 23_PCarbon 13NMR
165.19
159.67
158.65
155.40
154.37
138.06
130.24
128.02
116.35
112.37
89.79
77.70
77.48
77.27
60.40
40.93
27.83
25.92
21.02
18.22
14.25
10.25
H3C
CH3
NH
N
S
CH3
N
H3C
N
N
CH3
O
H3C
f1 (ppm)

Compound 23_MS

UB Mass Spectrometry Facility - SmartFormula Report

Analysis Info

Analysis Name	D:\Data\IC_3-21-19\5651a_000001.d	Acquisition Date	3/21/2019 7:16:36 PM
Method	Bruker_11052015	Operator	
Sample Name	5651a	Instrument	solariX
Comment			

Acquisition Parameters

Acquisition Mode	Single MS	Nebulizer gas flow rate	1.2 bar
Polarity	Positive	Drying Gas Temperature	180.0 °C
Broadband Low Mass	147.4 m/z	Drying Gas Flow Rate	4.2 L/min
Broadband High Mass	1500.0 m/z	Calibration Date	Thu Sep 27 10:56:48 2018
Time of Flight to Detector	0.001 sec	Data Acquisition Size	1048576
Ion Accumulation Time	0.100 sec	Data Processing Size (SI)	2097152
Acquired Scans	20	Apodization	Full-Sine
Capillary	6000.0 V		

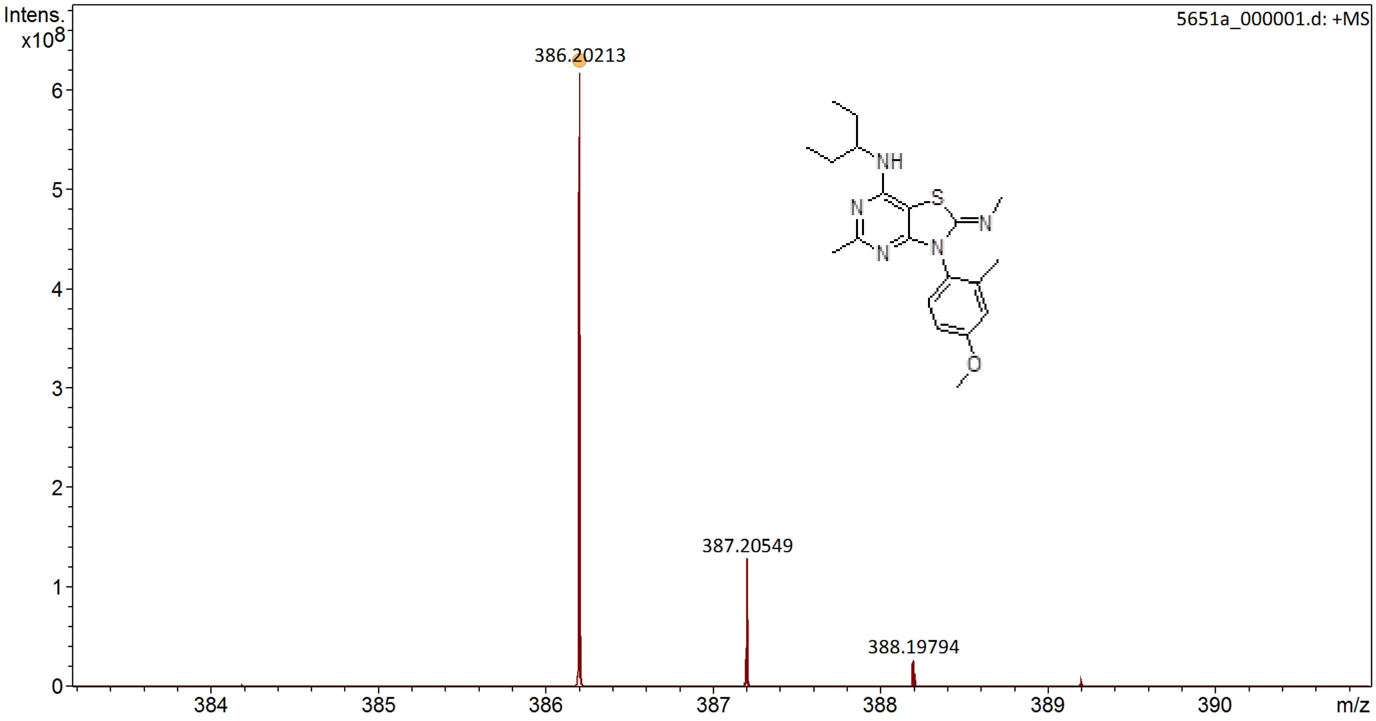

Adduct	Meas. m/z	#	Ion Formula	Score	m/z	err [ppm]	Mean err [ppm]	mSigma	rdb	e¯ Conf	N-Rule
M+H	386.202134	1	C20H28N5OS	100.00	386.200908	-3.2	437.7	14.4	9.5	even	ok
M+Na	408.183871	1	C20H27N5NaOS	100.00	408.182852	-2.5	414.5	14.4	9.5	even	ok
